# Supplementary material for: Assessing the effect of closed-loop insulin delivery from onset of type 1 diabetes in youth on residual beta-cell function compared to standard insulin therapy (CLOuD study): a randomised parallel study protocol
Source: BMJ Open. 2020 Mar 12;10(3):e033500. doi: 10.1136/bmjopen-2019-033500 (PMC7069267; doi:10.1136/bmjopen-2019-033500)
Supplement: Supplementary data [file bmjopen-2019-033500supp001.pdf]

Supplementary Appendix

Table of Contents

1.	Extension phase	page 2
2.	Training details	page 3
3.	FlorenceM closed-loop system	page 4
4.	CamAPS FX closed-loop system	page 5
5.	Blood sampling schedule	page 6
6.	Questionnaires	page 7
7.	Cogstate	page 8
8.	Composition of study management groups	page 9
9.	Example Consent Form	page 10
10.	Statistical Analysis Plan	page 14
11.	Multiple comparisons diagram	
12.	Study Protocol	

Boughton C, *et al. BMJ Open* 2020; 10:e033500. doi: 10.1136/bmjopen-2019-033500

1. **Extension Phase**

At 24 months, all participants will be invited to participate in an optional extension phase to continue with their current treatment (closed-loop insulin delivery or standard therapy) for a further 24 months.

The objective of the extension phase is to evaluate the effect of continued intensive metabolic control using closed-loop insulin delivery after diagnosis on preservation of C-peptide residual secretion by comparing the fasting C-peptide and glucose levels conducted at the 36 and 48 month visit in participants receiving closed-loop insulin delivery with those receiving standard therapy.

The extension phase will also examine the efficacy of closed-loop compared with standard therapy on glucose control comparing between group differences in HbA1c, and parameters based on masked continuous glucose monitoring (CGM). The safety of closed-loop will be evaluated in terms of episodes of severe hypoglycaemia and other adverse events. The frequency and duration of use of the closed-loop system will be assessed. Emotional and behavioural characteristics of participating subjects and family members and their response to their diabetes therapy and clinical trial will be evaluated using questionnaires.

Boughton C, *et al. BMJ Open* 2020; 10:e033500. doi: 10.1136/bmjopen-2019-033500

2. Pre Randomisation Training

Participants and their families will be educated in blood glucose monitoring, blood glucose targets and the benefits of maintaining optimal glycaemic control for long term health, use and administration of insulin therapy (including handling of insulin pens and injections), physical activity advice, hypoglycaemia recognition and management, dealing with hyperglycaemia (including correction doses and blood ketone monitoring), sick day rules, and carbohydrate counting and dietetic education.

Post Randomisation training

Closed-loop training

Particular attention will be paid to insulin cartridge and infusion set changes and correct priming procedure, carbohydrate counting and the use of the bolus calculator on the pump, hypo- and hyperglycaemia management using an insulin pump, sensor insertion and calibration and uploading pump data. Participants will be instructed on the use of low-glucose suspend functionality and settings. Hypoglycaemia and hyperglycaemia alarms can be activated and personalised as per participants' requirements

Participants will be trained on connection and disconnection of the closed-loop system and switching between closed-loop and usual pump therapy. During the closed-loop period, meal boluses will be delivered by the insulin pump based on carbohydrate estimation. Specific instructions during closed-loop related to exercise management, sick day rules, hypo- and hyperglycaemia management and technical troubleshooting will also be reviewed with the participants by the study team.

Standard therapy

Particular attention will be paid to carbohydrate counting, understanding insulin to carbohydrate ratios and correction factors, data review, pattern recognition and adjusting insulin doses.

Boughton C, *et al. BMJ Open* 2020; 10:e033500. doi: 10.1136/bmjopen-2019-033500

3. Florence M closed-loop system

The FlorenceM closed-loop system comprises:

- Modified 640G pump (Medtronic, Northridge, CA, USA)
- Guardian 3 sensor (Medtronic)
- A locked down Android smartphone with enclosure (Medtronic) hosting FlorenceM App with the model predictive control algorithm (University of Cambridge, Cambridge, UK) and communicating wirelessly with the insulin pump.

Further details: *The FlorenceM closed-loop system utilises a model predictive control algorithm residing on an Android lockdown smartphone that communicates wirelessly with the modified insulin pump using a proprietary enclosure. Every 10 minutes, the system calculates a new temporary basal insulin infusion rate, which is automatically sent to the insulin pump. The control algorithm is initialised using pre-programmed basal insulin delivery from the pump. Information about participants' weight and total daily insulin dose will be entered at setup. During closed-loop operation, the algorithm learns and adapts to a particular participant. The treat-to-target control algorithm aims to achieve a default glucose level of 5.8 mmol/L, adjusting the actual level depending on fasting versus postprandial status and accuracy of model-based glucose predictions. No remote monitoring is instigated. The pump comprises a CGM receiver and provides hypo/hyperglycaemia alarms which can be activated and personalised by participants. The pump provides standard insulin pump alarms, and the smartphone alerts the user about aspects related to closed-loop operations.*

Safety precautions during closed-loop

Participants will be instructed to perform calibration checks before breakfast and evening meals. If sensor glucose differs from finger-stick glucose by >3.0mmol/L, the glucose sensor will be recalibrated. If sensor glucose becomes unavailable, pre-programmed insulin delivery will be automatically started within 30 min, limiting the risk of insulin under- or over-delivery. Safety rules limit maximum insulin infusion and suspend insulin delivery at sensor glucose ≤4.3mmol/L or when sensor glucose is rapidly decreasing. If closed-loop becomes unavailable due to loss of sensor glucose input or other system failures, insulin delivery will be suspended by the low glucose suspend feature on the insulin pump, provided sensor glucose is available. Resumption of insulin delivery will be in accordance with the pump low glucose suspend feature.

Boughton C, *et al. BMJ Open* 2020; 10:e033500. doi: 10.1136/bmjopen-2019-033500

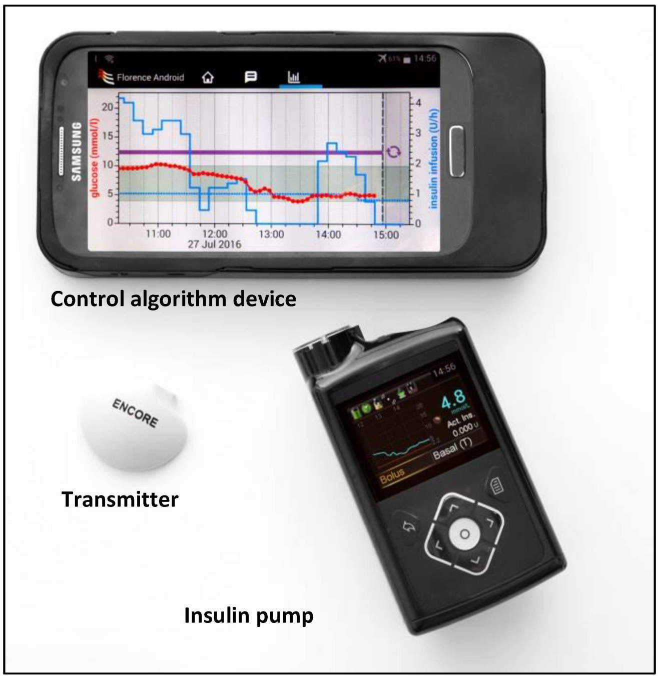
Control algorithm device
Transmitter
Insulin pump

4. CamAPS FX closed-loop system

The CamAPS FX closed-loop system comprises:

- Dana RS insulin pump (Diabecare, Sooil, Seoul, South Korea)
- Factory calibrated Dexcom G6 real-time CGM sensor (Dexcom, San Diego, CA, USA)
- An Android smartphone hosting CamAPS FX App with the model predictive control algorithm (University of Cambridge, Cambridge, UK) and communicating wirelessly with the insulin pump and CGM transmitter
- Real-time upload capabilities to diabetes management system Diasend (Glooko/Diasend, Göteborg, Sweden).

Further details: *Apart from connecting to a different pump and a different CGM device compared to FlorenceM, CamAPS FX App resides on an unlocked Android phone and is capable of uploading data to Diasend. The control algorithm, setup procedure, and target glucose levels are identical between FlorenceM and CamAPS FX systems.*

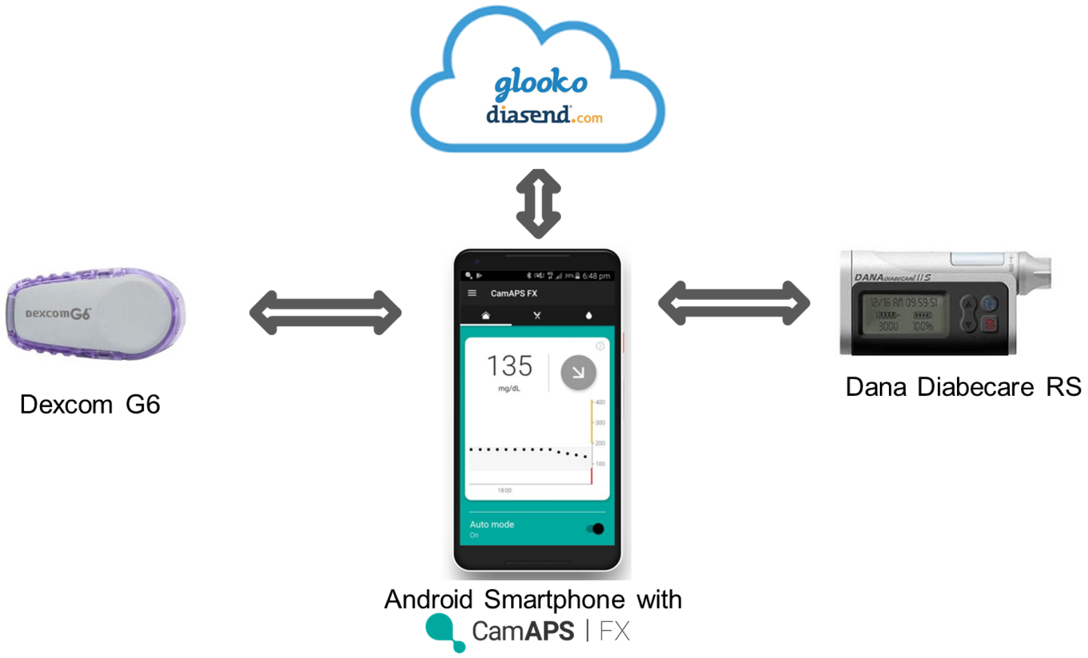

Boughton C, *et al. BMJ Open* 2020; 10:e033500. doi: 10.1136/bmjopen-2019-033500

5. Blood sampling schedule

Test	Visit 2 baseline	Visit 6 3 months	Visit 7 6 months	Visit 8 9 months	Visit 9 12 months	Visit 10 15 months	Visit 11 18 months	Visit 12 21 months	Visit 14 24 months	Visit 15 36 months	Visit 17 48 months
C-peptide	7x		7x		7x				7x	1x	1x
Glucose	7x		7x		7x				7x	1x	1x
Lipid profile	1x		1x		1x				1x	1x	1x
HbA1c	1x	1x	1x	1x	1x	1x	1x	1x	1x	1x	1x
Immunology	3x		2x		2x				2x		

Boughton C, *et al. BMJ Open* 2020; 10:e033500. doi: 10.1136/bmjopen-2019-033500

6. Questionnaires

Measure	*Respondent*	*Construct Measured / Relevant Points*	*Time-point*
Paediatric Quality of Life Inventory (PedsQL) Diabetes	All youth and all parents	All youth ages 10-18 will complete age appropriate PedsQL Diabetes module. There are 28 items. Parents will also complete a proxy version.	Baseline, 12, 24, 36 and 48 months
Strengths and Difficulties Questionnaire (SDQ)	All youth and all parents	This is a widely used 25 item self-report inventory behavioural screening questionnaire for children and adolescents. The same 25 items are included in questionnaires for completion by the parents.	Baseline, 12, 24, 36 and 48 months
Hypoglycaemia Fear Survey (HFS)	All youth and all parents	Validated questionnaires (HFS child version, HFS parent version) to measure several dimensions of fear of hypoglycaemia. They consist of a 10-item “Behaviour subscale” that measures behaviours involved in avoidance and over-treatment of hypoglycaemia and a 13-item “Worry subscale” that measures anxiety and fear surrounding hypoglycaemia.	12, 24, 36 and 48 months
Pittsburgh Sleep Quality Index (PSQI)	All youth and all parents	The PSQI is a validated 19 item questionnaire that holistically assesses sleep quality and sleep duration.	6, 12, 24, 36 and 48 months
INSPIRE	All youth and parents	Measures the psychological side of automated insulin delivery. Child (6-12) and Adolescent versions (13-18) have 18 items; Parent version has 21 items.	12, 24, 36 and 48 months
PAID-Teen	All youth	Measures 26 items related to the daily hassles of managing type 1 diabetes, and the degree of diabetes distress that arises from diabetes management.	12, 24, 36 and 48 months

Boughton C, *et al. BMJ Open* 2020; 10:e033500. doi: 10.1136/bmjopen-2019-033500

7. Cogstate

Using various stimuli, CogState measures a range of cognitive domains to give objective information regarding changes to a subject's cognitive function. The subtests include

- Groton Maze Learning Task - measures executive function using a maze learning paradigm. A 28-step pathway is hidden among a 10x10 grid of tiles. A blue tile indicates the start and a tile with red circles indicates the finish. The participant must move one step at a time from the start to the end by touching a tile next to their current location. If the correct move is made a green checkmark appears and if the move is incorrect a red cross is revealed. Once completed, they are returned to the start to repeat the test and must try to remember the pathway they have just completed.
- Detection Task - measures processing speed using a simple reaction time paradigm. A playing card is presented face down in the centre of the screen. As soon as the card flips over the participant must press "Yes". The participant is encouraged to work as quickly as they can and be as accurate as possible.
- Identification Task - measures attention using a choice reaction time paradigm. A playing card is presented face down in the centre of the screen. As soon as it flips over the participant must decide whether the card is red or not. The participant is encouraged to work as quickly as they can and be as accurate as possible.
- Two Back Task - measures working memory using an n-back paradigm. A playing card is presented face-up in the centre of the screen. The participant must decide whether the card is the same as the card shown two cards previously. The participant is encouraged to work as quickly as they can and be as accurate as possible.
- Continuous Paired Associate Learning Task - measures visual memory using a paired associative learning paradigm. The participant must learn and remember the pictures hidden beneath different locations on the screen. A picture is presented in the centre of the screen. The participant taps the peripheral location of the picture and must remember its location. During the second stage of the test the same pictures are presented in the centre of the screen, however the peripheral location of each picture is hidden. The participant must tap on the peripheral location where the picture previously appeared. Only participants aged 12 or over complete this test.
- Groton Maze Learning Task: Delayed Recall - measures visual memory

Boughton C, *et al. BMJ Open* 2020; 10:e033500. doi: 10.1136/bmjopen-2019-033500

8. Composition of trial management groups

Data Monitoring and Ethics Committee (DMEC) will comprise a chairperson and two experts.

Trial steering committee (TSC) will consist of an independent chairperson, two other independent experts in the field, a public and patient involvement (PPI) representative, and the Chief Investigator. Representatives of the Trial Sponsor and the Trial Funder will be invited to TSC meetings.

Trial management group (TMG) will consist of the Chief Investigator, Study Coordinators, and Study Data Manager. The Principal Clinical Investigators may also participate in the meetings of the TMG.

Boughton C, *et al. BMJ Open* 2020; 10:e033500. doi: 10.1136/bmjopen-2019-033500

9. Example Consent Form (Youth aged 16)

CLOuD Closed Loop from Onset in Type 1 Diabetes

Institutional logo

Patient ID Label:

Centre NRES number: ....

Patient identification number for this trial: ……………………..

CONSENT FORM
(Youth aged 16)

Title of Project: Closed Loop from Onset in Type 1 Diabetes (CLOuD)

Name of Researcher: ....

Please initial box

1. I confirm that I have read and understood the information sheet version .... dated .... for the above study and have had the opportunity to consider the information and I am satisfied with the answers I have been given. ☐

2. I understand that my participation is voluntary and that I am free to withdraw at any time, without giving any reason, without my medical care or legal rights being affected in any way. ☐

3. I agree to take part in the study, and to follow the study team's instructions for the purposes of the study. ☐

4. I give consent for the taking of blood and urine samples. I understand that these samples will be stored for up to 5 years after study completion, in an approved licensed laboratory and may be used for future research. ☐

5. I agree that the research may include analysis of DNA and that any genetic findings will not be shared with me or my doctor(s). ☐

6. If I want to change my mind in the future and withdraw my consent for this study, then I understand that, if I request it, the samples will be destroyed. ☐

7. If I choose to communicate with the research team via email, I acknowledge the associated risks as outlined in the information sheet. ☐

8. I give consent to my GP being informed of my participation in this study. ☐

205761_Consent Form Youth Aged 16_Version 3.0_ 24 January 2019 Page 1 of 3

Boughton C, *et al. BMJ Open* 2020; 10:e033500. doi: 10.1136/bmjopen-2019-033500

9. I give consent for the collecting of my medical data related to the study as outlined in the information sheet. ☐

10. I give consent to the sharing of anonymised research data arising from this study with other study partners, researchers and for publication purposes in Europe and outside Europe. This sharing of data may continue after the research study is completed. ☐

11. I understand that relevant sections of my medical notes and data collected during the study may be looked at by individuals from the NHS Trust and regulatory bodies for audit or monitoring purposes. ☐

12. I agree if selected to participate in the qualitative interview. ☐

    a. I agree for my contact details to be given to the study psychologist. ☐

    b. I agree for my family member/partner's contact details to be given to the study psychologist and the research team. ☐

    c. I agree for my interview to be digitally recorded. ☐

    d. I understand that anonymised quotes may be used in reports and publications. ☐

13. I agree to participate in the focus group discussion and for it to be digitally recorded. ☐

    a. I agree for my family member/partner's contact details to be given to the research team. ☐

    b. I agree for the focus group discussion to be digitally recorded. ☐

    c. I understand that anonymised quotes may be used in reports and publications. ☐

14. I agree to complete the online cognitive tests. ☐

15. I agree to perform the sleep study wearing the watch and completing the sleep diary and questionnaire. ☐

16. If I decide to take part in the peer support system, I agree that all information shared is strictly confidential. ☐

17. I agree to return the study devices at the end of the study or earlier if consent is withdrawn. ☐

18. I understand that in the unlikely event that study devices are not returned, legal measures may be undertaken, as a last resort after all other options have been exhausted, to recover the study devices. ☐

19. I give consent for my personal data to be kept by the clinical research team for up to 5 years after termination of the study so that they may contact me in the future with results of this and other similar studies. ☐

20. I agree to the research team collecting data about my diabetes treatment/management from my diabetes clinic for up to 9 years after study completion. Data will be anonymised and may be used for further research. ☐

21. I give consent for the collecting of my medical data related to the study and transfer to a data controller and/or processor located in USA, as outlined in the information sheet. ☐

Name of participant	Date	Signature
Name of Person taking consent (if different from researcher)	Date	Signature
Researcher	Date	Signature

Copies: 1 for volunteer; 1 for researcher; 1 to be kept with hospital notes

Boughton C, *et al. BMJ Open* 2020; 10:e033500. doi: 10.1136/bmjopen-2019-033500

10. Statistical Analysis Plan

Boughton C, *et al. BMJ Open* 2020; 10:e033500. doi: 10.1136/bmjopen-2019-033500

Closed Loop from Onset in type 1 Diabetes (CLOuD)

Statistical Analysis Plan

Version 2.0

April 4, 2019 (Based on Protocol Version 5.0)

Boughton C, *et al. BMJ Open* 2020; 10:e033500. doi: 10.1136/bmjopen-2019-033500

Revision History

The following table outlines changes made to the Statistical Analysis Plan.

Version Number	Author	Approver	Effective Date	Study Stage
1.0	Nathan Cohen	Craig Kollman	02/09/2018	Enrollment started
1.1	Nathan Cohen	Craig Kollman	05/07/2018	Enrollment started
2.0	Nathan Cohen	Peter Calhoun	04/04/2019	Enrollment started

Version Number	Revision Description
1.1	Added the INSPIRE and PAID-Teen questionnaires. Also added clarifications on how we will handle venous and capillary HbA1c. Reorganized calculation of secondary outcomes.
2.0	Added the Cogstate questionnaire and analyses for the extension phase.

Author: ________________________________

Senior Statistician: ________________________________

Jaeb Principal Investigator:______________________________

Boughton C, *et al. BMJ Open* 2020; 10:e033500. doi: 10.1136/bmjopen-2019-033500

1 Statistical and Analytical Plans

This document outlines the statistical analyses to be performed for the CLOuD study. The approach to sample size and statistical analyses for this study are detailed below.

The following analyses to be conducted for this study will not be performed at the Jaeb Center for Health Research and are only briefly mentioned in this document:

- Exploratory analysis of the relationship between beta-cell function and immune markers
- Human factors analyses
- Health economic evaluations

2 Statistical Hypotheses

- *Null Hypothesis:* There is no difference in the population mean area under the meal stimulated C-peptide curve at 12 months after diagnosis of T1D between the two treatments.
- *Alternative Hypothesis:* There is a nonzero difference in the population mean area under the meal stimulated C-peptide curve at 12 months after diagnosis of T1D between the two treatments.

3 Sample Size

Assuming a mean area under the meal stimulated C-peptide curve of 0.37 pmol/ml for the control group based on the lower 90% confidence limit from previous data, a 50% increase in the intervention group gives 0.37*1.50 = 0.555 pmol/ml. After a ln(x+1) transformation, the mean values in the control and treatment groups are 0.315 and 0.441, respectively, giving a treatment effect of 0.441-0.315=0.126. The treatment effect of 0.126 with a standard deviation of 0.18 requires 44 subjects per group at 90% power for a two sided-test at the 0.05 level. Allowing for 10% loss to follow up means we would need a total of 96 randomized participants (48 per group).

4 Outcome Measures

Primary Efficacy Endpoint:

Mean stimulated C-peptide AUC at 12 months post diagnosis

Key Secondary Endpoints:

- Percent time in the target range (3.9-10.0 mmol/L) at 12 months
- HbA1c at 12 months
- Percent time below 3.9 mmol/L at 12 months

Secondary Efficacy Endpoints*:

C-Peptide

1) Mean stimulated C-peptide AUC at at 6 and 24 months post diagnosis
2) Fasting C-peptide divided by fasting glucose at 6, 12, 24, 36 and 48 months

HbA1c Outcomes

2) HbA1c levels at 24, 36, and 48 months

Boughton C, *et al. BMJ Open* 2020; 10:e033500. doi: 10.1136/bmjopen-2019-033500

3) Percentage of patients in each group with HbA1c <7.5% (58 mmol/mol) at 12, 24, 36, and 48 months

CGM Outcome Metrics

Overall Glucose Control

4) Percentage of time spent with sensor glucose in the target range (3.9 to 10 mmol/L) at 24, 36, and 48 months
5) Mean of glucose levels
6) Standard deviation of glucose levels
7) Coefficient of variation of glucose levels

Hyperglycemia

8) Percent Time spent with glucose levels above 10.0 mmol/L
9) Percent Time spent with glucose levels above 16.7 mmol/L

Hypoglycemia

9) Percent Time spent with glucose levels below 3.9 mmol/L at 24, 36, and 48 months
10) Percent Time spent with glucose levels below 3.5 mmol/L
11) Percent Time spent with glucose levels below 3.0 mmol/L
12) Percent Time spent with glucose levels below 2.8 mmol/L
13) Area under the curve of glucose levels below 3.9 mmol/L
14) Area under the curve of glucose levels below 3.5 mmol/L

Insulin Delivery

15) Total insulin dose (U/day/kg)
16) Basal insulin dose (U/day/kg)
17) Bolus insulin dose (U/day/kg)

Weight

18) Change in body mass index (BMI) standard deviation score

Blood Pressure

19) Systolic blood pressure
20) Diastolic blood pressure

Lipid Profile

21) Total cholesterol
22) Triglycerides
23) HDL cholesterol
24) LDL cholesterol

*All secondary endpoints will be compared between treatment groups at 12 and 24 months post diagnosis of T1D. Additionally, secondary endpoints will be compared at 36 and 48 months for subjects who opt to enroll in the extension phase of the study.

4.1 Calculation of the Primary Outcome

For each mixed meal tolerance test (MMTT), the baseline C-peptide value will be defined as the mean of measurements taken at -10 and 0 minutes. If one of these measurements is missing, then the other one will be used as baseline. In the calculation, the starting time of the MMTT will be defined as t=0 if the C-peptide value is available at that time point. Otherwise, it will be set to t=-10.

The stimulated C-peptide AUC will only be calculated for MMTTs with an available baseline measurement and at least three other C-peptide measurements inclusive of the 90 or 120 minute measurements. In other words, the primary outcome will be calculated for all MMTTs with all the following conditions satisfied:

- At least one C-peptide measurement taken at -10 or 0 minutes
- At least three C-peptide measurements from those taken at 15 minutes, 30 minutes, 60 minutes, 90 minutes, and 120 minutes
- At least one C-peptide measurement taken at 90 minutes or 120 minutes

For all MMTTs with enough available C-peptide data, the trapezoidal rule will be used to calculate the stimulated C-peptide AUC. This involves computing a weighted sum of the C-peptide measurements. Throughout the calculation, target times for C-peptide measurements will be used (as opposed to the actual measurement times).

If the C-peptide measurement at 120 minutes is not available, the following steps will be used to calculate AUC:

- Calculate AUC_{90} from C-peptide measurements taken between baseline and 90 minutes
- Calculate AUC as $AUC_{90} \times (120/90)$

4.2 Calculation of Secondary Outcomes

CGM Metrics

Each of the sensor glucose metrics listed above will be calculated over 24 hours unless otherwise noted. The metrics will be calculated based on up to 14 days of blinded CGM at 3-month intervals. Specifically, participants in both trial arms will be fitted with a blinded glucose sensor at study visit 2, which will be used to calculate baseline CGM metrics for each participant. Thereafter, participants in both arms will be fitted with blinded glucose sensors at 3, 6, 9, 12, 15, 18, 21, and 24 months post diagnosis of T1D. Additionally, subjects who opt to participate in the extension phase of the study will be fitted with a blinded sensor at months 36 and 48. A single percentage will be calculated for each subject at each of these visits by pooling all blinded CGM readings corresponding to the 14 day period. Similarly, baseline CGM metrics will be calculated using up to two weeks of CGM data fitted at study visit 2.

HbA1c

In all secondary analyses involving HbA1c, the venous HbA1c value will be used at baseline, 6 months, 12 months, and 24 months. At all other time points, the capillary HbA1c value will be used instead.

During the extension phase, HbA1c will be measured by the central lab at 36 and 48 months. At all other time points, local HbA1c will be measured instead.

CL System Use (in the CL arm only)

CL system use will be calculated using logs generated by the control algorithm. Any temporary basal insulin rate lasting less than 30 minutes will be considered to indicate system being in CL.

Boughton C, *et al. BMJ Open* 2020; 10:e033500. doi: 10.1136/bmjopen-2019-033500

5 Description of Statistical Methods

5.1 General Approach

This is an open-label, multi-center, single-period, randomized, parallel group design study of participants aged 10 to <17 years. It is expected that a total of up to 190 subjects will be recruited within ten working days of diagnosis of type 1 diabetes (T1D) through pediatric diabetes centers in the UK. Half of the participants will be treated by conventional multiple daily injection therapy (MDI) and the other half by closed loop insulin delivery system (CL). Initiation of treatment arm occurs at study visit 5 and will last 24 months. Additionally, subjects will have the option to participate in an extension phase that will go until 48 months.

The study includes up to 14 visits and 1 telephone/email contact for subjects completing the study. For subjects completing the extension phase, it will include an additional 3 visits and 6 contacts. After initiation of the treatment arm, visits will be conducted every 3 months in both arms during the main study and the optional extension phase. Beta-cell function will be assessed by serial measurement of C-peptide in response to a standardized MMTT. MMTTs will be conducted at baseline, 6, 12, and 24 months post diagnosis.

All analyses will compare the CL arm with the MDI arm and will follow the intention-to-treat principle with each participant analyzed according to the treatment assigned by randomization regardless of actual CL utilization. All randomized participants who have at least one CGM reading during the 24 month study period will be included in the secondary analyses of CGM metrics. For secondary analyses of CGM metrics after 24 months, only subjects with at least one reading occurring during the extension phase (i.e. after the date of the 24 month visit) will be included. The primary analysis will include all randomized participants.

All covariates obtained on a continuous scale will be entered into the models as continuous variables, unless it is determined that a variable does not have a linear relationship with the outcome. In such a case, categorization and/or transformation will be explored. All p-values will be two-sided.

Standard residual diagnostics will be performed for all analyses. If values are highly skewed, then an alternate transformation or nonparametric methods will be used instead for the primary outcome. Previous experience suggests that a transformation or nonparametric analyses may be necessary for the hypoglycemic and hyperglycemic outcomes. We do not expect that a transformation or nonparametric analyses will be necessary for HbA1c or some CGM metrics for assessing overall glucose control (such as mean glucose or percent time-in-range).

5.2 Analysis Cohorts

- All randomized participants will be analyzed for the Intention-to-Treat (ITT) Analysis. All randomized subjects with at least one CGM reading will be included in CGM analyses.
- Safety outcomes will be reported for all enrolled participants, irrespective of whether the study was completed.
- A per-protocol analysis restricted to participants:

Boughton C, *et al. BMJ Open* 2020; 10:e033500. doi: 10.1136/bmjopen-2019-033500

- in the CL group who used the CL system at least 60% of the time
- in the MDI group who do not start insulin pump therapy

will be conducted at 12 and 24 months post diagnosis for:

- the primary outcome (C-peptide AUC)
- HbA1c
- percentage of time with sensor glucose levels in the target range (3.9-10.0 mmol/L).

Additionally, per-protocol analyses also will be conducted for HbA1c and the percentage of time spent in the target range at 36 and 48 months.

The per-protocol analyses for the percentage of time in the target range will be done separately at each time point, so the analyses at 24, 36, and 48 months will not involve pooling CGM data at 12 months too. The per-protocol analyses will only be performed if at least 5% of the participants do not meet the criteria above to be included in the analysis.

6 Primary Analysis

6.1 Out Of Window Visits

All MMTTs occurring between 300 and 420 days after the diagnosis date will be included in the primary analysis. Any MMTTs occurring outside this window will be excluded.

6.2 Statistical Methods

The values obtained at 12-months post-randomization for log(C-peptide AUC+1) for the CL and MDI arms will be compared using a linear model adjusting for baseline log(C-peptide AUC+1), gender, presence or absence of DKA at diagnosis, and age as fixed effects and clinical site as a random effect. The mean adjusted difference between treatment groups and the corresponding 95% confidence interval from the linear model will be reported. If residual values from the regression model have a skewed distribution then an appropriate alternate transformation or a nonparametric analysis based on ranks will be performed.

Per the protocol, any of the following variables may also be included in the model as fixed effects to assess for the presence of confounding:

- HbA1c levels
- BMI z-score
- Race/ethnicity

Imbalances between groups in other important covariates are not expected to be of sufficient magnitude to produce confounding. However, the presence of confounding will be evaluated in the primary analysis by including any other factors potentially associated with the outcome for which there is an imbalance between groups.

The primary analysis will be a single comparison and no attempt will be formally made to control the overall type I error rate. A 5% significance level will be used to declare statistical significance for the primary comparison.

6.3 Missing Data

6.3.1 Direct Likelihood

In the event that some C-peptide AUC values are not available at 12 months, then the primary analysis will use the method of direct likelihood to incorporate information from previous measurements to calculate the maximum likelihood estimate at 12 months.

It is worth noting that all statistical methods for handling missing data rely on untestable assumptions and there is no one correct way to handle missing data. Our goal is to minimize the amount of missing data so that the results will not be sensitive to which statistical method is used.

6.3.2 Sensitivity Analyses

Sensitivity analyses will also be performed to assess the effects of the statistical method for missing data on the primary outcome. Analyses will be performed using the following two methods:

- Available cases only
- Rubin's multiple imputation based on previous C-peptide AUC measurements at baseline or 6 months

7 Analysis of the Secondary Endpoints

The following table displays the times at which each type of outcome metric listed in Section 4 is measured:

Outcome Metric	Baseline	3 Months	6 Months	9 Months	12 Months	15 Months	18 Months	21 Months	24 Months
C-Peptide AUC	X		X		X*				X
Fasting C-peptide/fasting glucose	X		X		X				X
HbA1c Metrics	X	X	X	X	X	X	X	X	X
CGM Metrics	X	X	X	X	X	X	X	X	X
Insulin Metrics	X	X	X	X	X	X	X	X	X
Height/Weight (to calculate BMI)	X		X		X				X
Blood Pressure	X		X		X				X
Lipid Profile Metrics	X				X				X

* Primary outcome

In the table above, each metric is collected at the time points marked with an "X".

Additionally, the following table displays the times at which each type of outcome metric is measured during the extension phase:

Outcome Metric	27 Months	30 Months	33 Months	36 Months	39 Months	42 Months	45 Months	48 Months
C-Peptide				X				X
Fasting C-peptide/fasting glucose				X				X
Fasting Blood Glucose				X				X
HbA1c Metrics	X	X	X	X	X	X	X	X
CGM Metrics				X				X
Insulin Metrics	X	X	X	X	X	X	X	X
Height/Weight (to calculate BMI)				X				X

Boughton C, *et al. BMJ Open* 2020; 10:e033500. doi: 10.1136/bmjopen-2019-033500

Blood Pressure								
Lipid Profile Metrics				X				X

As shown above, blood pressure will not be measured during the extension phase. There also will not be any MMTTs occurring during the extension phase. Only C-peptide and fasting blood glucose will be measured during the extension phase. Values for those two metrics will be compared between treatment groups at 36 and 48 months as described below.

Per the protocol, lipid profile metrics are not considered to be secondary endpoints during the extension phase, so they will not be compared between treatment groups at 36 and 48 months. Instead, only summary statistics will be reported at those time points.

7.1 Out Of Window Visits

As shown in the table above, each of the following variables are collected at study follow-up visits:

- C-peptide
- HbA1c
- Height and weight to calculate BMI
- Blood pressure
- Lipids

For each of these metrics, analysis windows will be as follows:

Visit	Window
3 months	Days 60 to 120
6 months	Days 150 to 210
9 months	Days 240 to 299
12 months	Days 300 to 420
15 months	Days 421 to 480
18 months	Days 510 to 570
21 months	Days 600 to 660
24 months	Days 661 to 780
27 months	Days 781 to 840
30 months	Days 870 to 930
33 months	Days 960 to 1020
36 months	Days 1021 to 1140
39 months	Days 1141 to 1200
42 months	Days 1230 to 1290
45 months	Days 1320 to 1380
48 months	Days 1381 to 1500

These windows do not apply to the CGM metrics, which are calculated as described above.

Boughton C, *et al. BMJ Open* 2020; 10:e033500. doi: 10.1136/bmjopen-2019-033500

7.2 Baseline BMI Standard Deviation Scores and Blood Pressure

Calculation

Baseline BMI will be calculated using height and weight measurements taken at the recruitment visit.

Baseline systolic and diastolic blood pressure measurements will be taken to be the values recorded at the baseline MMTT (if both are known) because it is closer to randomization than the recruitment visit. If either of these values are missing at the baseline MMTT, then the values obtained during the recruitment visit will be used instead (if both are known). If at least one of these values is missing at the baseline MMTT and the recruitment visit, then the values may be taken from different visits. The value at the baseline MMTT will be used if it is known. Otherwise, the value at recruitment will be used instead.

Inclusion in Statistical Models

These baseline values will be included in the models as covariates when comparing outcomes 18)-20) listed above between treatment arms.

7.3 Regression Modeling

At 12 months post diagnosis, a longitudinal analysis will be conducted for the primary outcome and each continuous secondary outcome using a linear mixed model with a random intercept and slope. For binary outcomes, a repeated measures logistic regression model will be used. All data collected up to and including the 12 month visit will be used to fit the models. The models will account for repeated measures coming from the same subject using an unstructured covariance matrix. They will adjust for baseline value, gender, presence or absence of DKA at diagnosis, and age as fixed effects, in addition to site as a random effect. If necessary, they may also adjust for HbA1c levels, BMI z-score, or race/ethnicity to assess for the presence of confounding. Residual values will be examined for an approximate normal distribution. If values are highly skewed, then a transformation or non-parametric methods will be used instead.

Using these longitudinal models, one treatment arm comparison will be performed specifically at 12 months for each metric and another treatment arm comparison will be performed combining data over all follow-up visits. The latter will be calculated using a linear contrast giving equal weight to each follow-up visit.

A composite analysis will not be performed for the lipid profile metrics, because they are not collected at any time points between the baseline visit and the 12 month visit (see table above).

An analogous longitudinal model will also be run at 24 months for each outcome metric, and at 36 and 48 months for subjects who participated in the extension phase.

In addition to the longitudinal models, boxplots will be used to display the distributions of the primary and secondary outcomes over time. If the longitudinal model produces a significant composite p-value for a particular outcome, then the boxplots will be used to explore the time at which the outcome began to differ between treatment arms.

Boughton C, *et al. BMJ Open* 2020; 10:e033500. doi: 10.1136/bmjopen-2019-033500

7.4 Analysis of C-Peptide AUC at 6 Months

If the primary outcome is declared significant, then it will also be compared between treatment groups at six months using the same approach described above.

7.5 Missing Data

In the event that a particular outcome metric is unavailable at 12, 24, 36, or 48 months but data is available at previous time points, it will be estimated using the method of direct likelihood described in section 6.3.1. This method will be applied to all applicable secondary outcomes.

In the HbA1c analyses, the primary outcome will be venous HbA1c, and capillary HbA1c will be treated as an auxillary variable.

Missing CGM data will not be imputed in this study.

7.6 Human Factors Evaluations

7.6.1 Questionnaires

Associations between questionnaire scores and T1D variables, such as residual C-peptide secretion, HbA1c, episodes of serious hypoglycemia, glycemic variability, and DKA at each time point and over time will be examined. The analysis will be performed by Lis Northam.

7.6.2 Quality of Sleep Assessment

The Pittsburgh Sleep Quality Index (PSQI) and actigraphy data will be used to calculate mean total sleep quality score, sleep duration, time in bed, sleep disturbance (including wake after sleep onset and number of awakenings), latency, efficiency, quality, and daytime dysfunction. The analysis will be conducted by Eleanor Scott.

7.6.3 Qualitative Interviews

The qualitative interviews will be analyzed thematically using the method of constant comparison. The analysis will be conducted by Julia Lawton.

7.6.4 Focus Groups

Focus groups will take place at 24 months with participants and their parents (8-12 participants per group, from both study arms) at each of the study sites. A script of open ended questions will be used to gather feedback and reactions to study recruitment and randomization, study support systems, and the closed loop system. There will also be time for discussion of content raised by participants. The analysis will be conducted by Conor Farrington.

7.7 Health Economic Evaluation

A health economic evaluation based on short term clinical results and costs of interventions will be conducted by Stéphane Roze. The analysis will project costs and clinical benefits into longer term endpoints.

8 Safety Analyses

A reportable adverse event for this protocol is any untoward medical occurrence that meets criteria for a serious adverse event or any unanticipated medical occurrence in a study subject that is study or device-related. Device deficiencies that could have led to a serious adverse device effect will also be reported.

Boughton C, *et al. BMJ Open* 2020; 10:e033500. doi: 10.1136/bmjopen-2019-033500

Safety data including severe hypoglycemic events and ketone-positive hyperglycemia will be tabulated for all subjects, including drop-outs and withdrawals, irrespective of whether CGM data are available and irrespective of whether closed loop was operational. For each event, the following information will be reported:

- Onset date of the event
- Resolution date of the event
- Duration of the event (in days)
- Description of the event
- Intensity of the event
- Seriousness of the event
- Whether the event was related to the study treatment
- Whether the event was related to the study procedure
- Whether the event was related to the study device
- Whether the event required treatment
- Outcome of the event

Separate listings will be provided for pre-randomization and post-randomization adverse events. For post-randomization events, the subject's treatment group will be included in the tabulation.

For the purpose of analysis, a severe hypoglycemic event will be defined as an event requiring assistance of another person actively to administer carbohydrate, glucagon, or other resuscitative actions. These episodes may be associated with sufficient neuroglycopenia including temporary impairment of cognition, incoherent, disoriented, and/or combative behavior, seizure, or coma. If plasma glucose measurements are not available during such an event, neurological recovery attributable to the restoration of plasma glucose to normal is considered sufficient evidence that the event was induced by a low plasma glucose concentration.

Summary statistics and analysis of safety outcomes will consist of the following:

1. Number of episodes of severe hypoglycemic events per subject and incidence rate per 100-person years
2. Number of subjects with severe hypoglycemic events
3. Number of episodes of DKA events per subject and incidence rate per 100-person years
4. Number of subjects with DKA events
5. Number of any other adverse events reported per subject
6. Number of any other serious adverse events reported per subject

Comparison of safety outcomes between the two treatment groups only include those events occurring on or after randomization until the last visit date or the last event date (whichever is later). Comparisons will be performed at 12 and 24 months post diagnosis of T1D. For subjects who continue into the extension phase, comparisons also will be done at 36 and 48 months.

For the binary safety outcomes listed above, a repeated measures logistic regression model will be used to compare treatment arms. For the count outcomes and the incidence rates, a Poisson regression model will be used. All models will adjust for site as a random effect. Additionally,

Boughton C, *et al. BMJ Open* 2020; 10:e033500. doi: 10.1136/bmjopen-2019-033500

the models for the DKA outcomes will adjust for presence of a DKA event at diagnosis. A p-value <0.05 will be used to declare statistical significance.

Any other adverse events will be listed by treatment group.

9 Adherence and Retention Analyses

The following tabulations and analyses will be performed by treatment group at 12 and 24 months post diagnosis (in addition to 36 and 48 months for subjects who continue into the extension phase) to assess protocol adherence for the study:

- Number of protocol and procedural deviations per subject along with the number and percentage of subjects with each number of deviations
- Number of protocol and procedural deviations by severity with brief descriptions listed
- Flow chart accounting for all subjects at all visits post treatment initiation to assess visit completion rates
- Number of and reasons for unscheduled visits

10 Baseline Descriptive Statistics

Baseline demographic and clinical characteristics of the cohort of all randomized participants will be summarized in a table using summary statistics appropriate to the distribution of each variable. Descriptive statistics will be displayed overall and by treatment group.

11 Planned Interim Analyses

No formal interim analyses or stopping guidelines are planned for this study.

The DSMB will review data collected for the study every six months (unmasked to assigned treatment arms). The data to be reviewed will include information regarding all of the following:

- Status of randomized participants
- Recruitment rates by month and by site
- Baseline demographic and clinical characteristics
- Dropped participants and reasons for discontinuing
- Reportable adverse events

12 Subgroup Analyses

A random center by treatment interaction effect will also be explored for the primary outcome. However, the study is not expected to have sufficient statistical power to formally assess for the presence of interaction. Interpretation of the center by treatment interaction will depend on whether the overall analysis demonstrates a significant treatment effect. In the absence of a significant treatment effect in the primary analysis, interpretation of the center by treatment interaction will be considered exploratory and used to suggest hypotheses for further investigation in future studies.

Summary statistics for the primary outcome will be reported by site in addition to the p-value for the interaction.

Boughton C, *et al. BMJ Open* 2020; 10:e033500. doi: 10.1136/bmjopen-2019-033500

13 Multiple Comparisons/Multiplicity

In this study, the primary analysis will be a single comparison of C-peptide AUC between the two treatment arms. The primary outcome will be tested at α=0.04.

For the secondary analyses, the following outcomes will be considered key endpoints:

- Percent time in the target range (3.9-10.0 mmol/L)
- HbA1c
- Percent time below 3.9 mmol/L

These three endpoints will be tested in a hierarchical fashion in the order listed above. If C-peptide AUC is significant at α=0.04, then they will be tested at α=0.05. Otherwise, they will be tested at α=0.01.

For all other secondary endpoints, the false discovery rate (FDR) will be controlled using the adaptive Benjamini-Hochberg procedure. The FDR will be calculated separately within each of the following categories:

- Secondary CGM Metrics
- Secondary Insulin Metrics
- Secondary HbA1c Metrics
- Secondary Clinical Metrics
- Subgroup Analyses and Sensitivity Analyses
- Per-Protocol Analyses
- Questionnaires

Due to the small number of subgroup analyses and sensitivity analyses, these will be combined into a single category. No adjustments for multiple comparisons will be done for safety outcomes.

14 Exploratory Analyses

14.1 Trends in Glucose Control and Insulin Delivery

Trends in overall glucose control and insulin delivery over time will be summarized in a table. For each of the following outcome metrics, summary statistics appropriate to the distribution will be reported by treatment arm every three months:

- Percentage of time spent with sensor glucose in the target range (3.9 to 10 mmol/L)
- Percentage of time spent with sensor glucose less than 3.0 mmol/L
- Mean of glucose levels
- Standard deviation of glucose levels
- Total insulin dose (U/day/kg)

Additionally, trends in CGM and insulin data collected within intervention arms will be evaluated on a 3-monthly basis. CGM and insulin data collected during intervention arms will also be compared to baseline data. This will be done for all secondary outcomes using boxplots displaying the distributions over time.

Boughton C, *et al. BMJ Open* 2020; 10:e033500. doi: 10.1136/bmjopen-2019-033500

14.2 Overall Glucose Control by Time of Day

Overall glucose control will also be summarized at daytime (8:00-23:59) and nighttime (0:00-7:59) at baseline, 12 months, and 24 months. Additionally, this will be done at 36 and 48 months for subjects who participate in the extension phase. For each of the following CGM metrics, summary statistics appropriate to the distribution will be reported by treatment arm and time of day:

- Percentage of time spent with sensor glucose in the target range (3.9 to 10 mmol/L)
- Mean of glucose levels
- Standard deviation of glucose levels
- Percentage of time spent with sensor glucose less than 3.0 mmol/L

14.3 Relationship between CL Compliance and Glucose Outcomes

The relationship between CL compliance and glucose outcomes will be assessed in the intervention arm at 12 and 24 months. Compliance also will be assessed at 36 and 48 months for subjects who continue into the extension phase. For each of the following glucose outcomes, a scatterplot of the glucose metric vs. the percentage of time using the CL system will be made:

- Percentage of time spent with sensor glucose in the target range (3.9 to 10 mmol/L)
- Mean of glucose levels
- Standard deviation of glucose levels
- Coefficient of variation of glucose levels
- Percent Time spent with glucose levels above 10.0 mmol/L
- Percent Time spent with glucose levels above 16.7 mmol/L
- Percent Time spent with glucose levels below 3.9 mmol/L
- Percent Time spent with glucose levels below 3.5 mmol/L
- Percent Time spent with glucose levels below 3.0 mmol/L
- Percent Time spent with glucose levels below 2.8 mmol/L
- Area under the curve of glucose levels below 3.9 mmol/L
- Area under the curve of glucose levels below 3.5 mmol/L

Results from these analyses will be interpreted with caution, as the potential pitfalls of compliance based analyses have been well documented. Subjects in the CL group who tend to use the CL system more frequently may inherently differ from subjects who use it less often. This may introduce bias into the analyses.

14.4 C-Peptide Correlations

The correlation matrix between fasting C-peptide, C-peptide AUC, and C-peptide at 90 minutes as assessed during the MMTT will be reported at baseline, 6 months, 12 months, and 24 months. The fasting C-peptide measurement will be set to be the baseline measurement as defined in section 4.1 above. Partial Spearman correlations controlling for treatment group will be reported at 6, 12, and 24 months. Baseline Spearman correlations will not control for treatment arm.

14.5 Relationship between Beta-Cell Function and Immune Markers

The analyses will be conducted by John Todd's group at Oxford University, UK, who will apply the latest phenotyping technologies to test for correlations between C-peptide decline and

Boughton C, *et al. BMJ Open* 2020; 10:e033500. doi: 10.1136/bmjopen-2019-033500

immunological parameters in PBMCs and in plasma. These methods include mass spectrometry, flow cytometry, single-cell transcriptome profiling and plasma proteomics and metabolomics.

15 Additional Analyses

15.1 Questionnaires

The following questionnaires will be completed at baseline, 12, and 24 months, in addition to 36 and 48 months for subjects who continue into the extension phase:

- Pediatric Quality of Life Inventory (PedsQL; participants and parents)
  - Diabetes Subscale
  - Treatment I Subscale
  - Treatment II Subscale
  - Worry Subscale
  - Communication Subscale
- Strengths and Difficulties Questionnaire (SDQ; participants and parents)
  - Emotional Symptoms Subscale
  - Conduct Problems Subscale
  - Hyperactivity/Inattention Subscale
  - Peer Relationship Problems Subscale
  - Total Difficulties Score
  - Prosocial Behavior Subscale
  - Internalizing Problems Subscale (Emotional Symptoms and Peer Relationship Problems Combined)
  - Externalizing Problems Subscale (Conduct Problems and Hyperactivity/Inattention Combined)
  - Impact Supplement
- Cognitive testing (CogState; participants only; not administered during the extension phase)
  - Identification Test Score
  - Detection Test Score
  - Two Back Test Score
  - Paired Associate Learning Test Score
  - Groton Maze Test Score

The following questionnaires will also be completed at 12 and 24 months, in addition to 36 and 48 months for subjects who continue into the extension phase:

- Hypoglycemia Fear Survey (HFS; participants and parents)
  - Behavior Subscale
  - Worry Subscale
- INSPIRE Survey (participants and parents; CL arm only)
- PAID-Teen (participants only)

The following questionnaires will also be completed at 6, 12, and 24 months, in addition to 36 and 48 months for subjects who continue into the extension phase:

- Pittsburgh Sleep Quality Index (PSQI; participants and parents)

Boughton C, *et al. BMJ Open* 2020; 10:e033500. doi: 10.1136/bmjopen-2019-033500

- Subjective Sleep Quality Subscale
- Sleep Latency Subscale
- Sleep Duration Subscale
- Habitual Sleep Efficiency Subscale
- Sleep Disturbances Subscale
- Use of Sleeping Medication Subscale
- Daytime Dysfunction Subscale

Missing Data

All participants are required to complete all questionnaires. Surveys cannot be submitted until all questions are answered.

Analysis

For each questionnaire, total scores will be tabulated by treatment group, in addition to the distribution of responses for each individual question. For the PedsQL survey, mean scores will be calculated instead of total scores. Results will be given separately for subject (for those old enough to fill out the questionnaire) and parent responses. Total scores (or mean scores for PedsQL) will also be provided for the various subscales and supplements within each questionnaire. No overall total score will be computed for the SDQ that involves every question (only a total difficulties score is computed from the questions comprising the Emotional Symptoms, Conduct Problems, Hyperactivity/Inattention, and Peer Problems Subscales).

For each full questionnaire, subscales, and supplements, a linear model will be used to compare total scores (or mean scores for PedsQL) between treatment groups. The Cogstate, PedsQL, SDQ, HFS, PAID-Teen, and PSQI surveys will be compared at 12, 24, 36, and 48 months. The PSQI survey will also be compared at 6 months. All models will adjust for site as a random effect and age, gender, and presence of DKA at diagnosis as fixed effects (in addition to any potential confounders). They will also adjust for baseline score as a fixed effect if the questionnaire is administered at baseline.

For the Cogstate survey, the distribution of responses for the individual questions will not be tabulated, because the electronic testing system does not provide them. For this questionnaire, only summary statistics for each of the five test scores and the total score for the entire survey will be provided.

Treatment arm comparisons will not be performed for the INSPIRE survey, because it is only administered to the CL arm. For this questionnaire, summary statistics at 12, 24, 36, and 48 months for the parent and participant versions in addition to the distribution of responses for each question will be reported. Separate versions of the questionnaire will be provided to participants aged 8-12 and those aged 13-18.

In the PSQI survey, some of the questions are open ended. For these questions, the participants may give ambiguous responses that make it impossible to score certain subscales. In these instances, it will also not be possible to calculate the overall total score for the entire questionnaire, because it depends on the scores for each subscale. Therefore, if a participant

provides an ambiguous response to an open ended question, the corresponding subscale will not be scored, and the total score for the whole questionnaire will not be calculated.

All analyses described above for questionnaires will include the same subjects that are included in the primary analysis.

Boughton C, *et al. BMJ Open* 2020; 10:e033500. doi: 10.1136/bmjopen-2019-033500

11. Multiple comparisons diagram

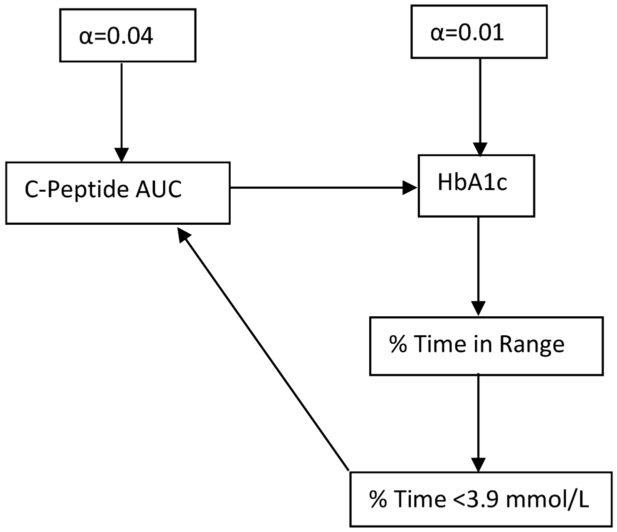

Boughton C, *et al. BMJ Open* 2020; 10:e033500. doi: 10.1136/bmjopen-2019-033500

12. Protocol

Clinical Study Protocol

Study Title: An open-label, multicentre, randomised, single-period, parallel design study to assess the effect of closed loop insulin delivery from onset of type 1 diabetes in youth on residual beta cell function compared to standard insulin therapy (CLOuD)

Short Title: **C**losed **L**oop from **O**nset in type 1 **D**iabetes (**CLO**u**D**)

Protocol Version: 8.0 10 September 2019

**Chief Investigator**	Prof Roman Hovorka<br>University of Cambridge Metabolic Research Laboratories<br>Level 4, Wellcome Trust-MRC Institute of Metabolic Science<br>Box 289, Addenbrooke's Hospital, Hills Rd<br>Cambridge CB2 0QQ<br>UK Tel: +44 (0)1223 762 862<br>Fax: +44 (0)1223 330 598<br>E-mail: rh347@cam.ac.uk

This protocol has been written in accordance with current ISO 14155:2011 standard

Boughton C, *et al. BMJ Open* 2020; 10:e033500. doi: 10.1136/bmjopen-2019-033500

**Principal Clinical Investigators**	Dr Ajay Thankamony<br>Consultant in Paediatric Endocrinology & Diabetes<br>Addenbrooke's Hospital<br>Cambridge University Hospitals NHS Foundation Trust<br>Children's Services, Box 267<br>Hills Road<br>Cambridge, CB2 0QQ<br>UK<br>Tel: +44 (0)1223 274 311<br>E-mail: ajay.thankamony@addenbrookes.nhs.uk<br><br>Dr Fiona Campbell<br>Consultant Paediatrician and Diabetologist<br>St James's University Hospital<br>Beckett Street, Leeds, LS9 7TF<br>UK<br>Tel: +44 (0)113 218 5908/9<br>E-mail: fiona.campbell26@nhs.net<br><br>Dr Atrayee Ghatak<br>Consultant Paediatric Endocrinologist and Diabetes Specialist<br>Alder Hey Children's NHS Foundation Trust<br>Eaton Road<br>West Derby<br>Liverpool, L12 2AP<br>UK<br>Tel: +44 (0) 151 228 4811<br>E-mail: Atrayee.Ghatak@alderhey.nhs.uk<br><br>Dr Tabitha Randell<br>Consultant in Paediatric Endocrinology and Diabetes<br>Deputy Head of Service,<br>Nottingham Children's Hospital<br>Dundee House<br>Hucknall Road<br>Nottingham, NG5 1PB<br>UK<br>Tel: +44 115 924 9924 ext 62336<br>E-mail: Tabitha.Randell@nuh.nhs.uk<br><br>Dr Rachel Besser<br>Clinical Lead in Paediatric Diabetes<br>John Radcliffe Hospital, Headley Way<br>Headington, Oxford, OX3 9DU

Boughton C, *et al. BMJ Open* 2020; 10:e033500. doi: 10.1136/bmjopen-2019-033500

	UK<br>Tel: +44 1865 234181<br>E-mail: rachel.besser@ouh.nhs.uk<br><br>Dr Nicola Trevelyan<br>Consultant Paediatrician and Diabetes Specialist<br>Southampton Children's Hospital<br>Mailpoint 43, G Level,<br>Southampton General Hospital,<br>Tremona Road,<br>Southampton, SO16 6YD<br>UK<br>Tel: +44 023 81 20 8518<br>E-mail: Nicola.Trevelyan@uhs.nhs.uk<br><br>Dr Daniela Elleri<br>Endocrine/Diabetes Department<br>Royal Hospital for Sick Children<br>9 Sciennes Road<br>Edinburgh, EH9 1LF<br>UK<br>Tel: +44 (0)131 536 0163<br>E-mail: Daniela.Elleri@nhslothian.scot.nhs.uk
**Clinical Investigators**	Dr Martin Tauschmann<br>Wellcome Trust-MRC Institute of Metabolic Science<br>Box 289, Level 4<br>Addenbrooke's Hospital<br>Hills Road<br>Cambridge CB2 0QQ<br>UK<br>Tel: +44 (0)1223 769 066<br>Fax: +44 (0)1223 330 598<br>E-mail: mt641@medschl.cam.ac.uk<br><br>Dr Gianluca Musolino<br>Wellcome Trust-MRC Institute of Metabolic Science<br>Box 289, Level 4<br>Addenbrooke's Hospital<br>Hills Road<br>Cambridge CB2 0QQ<br>UK<br>Tel: 01223 769069<br>Fax: +44 (0)1223 330 598<br><br>Dr Charlotte Boughton<br>Wellcome Trust-MRC Institute of Metabolic Science<br>University of Cambridge<br>Box 289, Level 4

	Addenbrooke's Hospital<br>Hills Road, Cambridge<br>CB2 0QQ<br>UK<br>Tel: +44 (0) 1223 769 066
**Other Investigators**	Professor David Dunger<br>Department of Paediatrics<br>University of Cambridge<br>Box 116, Level 8<br>Addenbrooke's Hospital<br>Hills Road<br>Cambridge , CB2 0QQ<br>UK<br>Tel: +44 (0)1223 336 886<br>Fax: +44 (0)1223 336 996<br>E-mail: dbd25@cam.ac.uk
**Study Co-ordinators**	Cambridge Clinical Trials Unit (CCTU)<br>Box 401, Coton House, Level 6<br>Cambridge University Hospitals NHS<br>Foundation Trust<br>Hills Road<br>Cambridge, CB2 0QQ<br>UK<br>Tel: + 44 (0)1223 596 474<br>Fax: + 44 (0)1223 256 763<br><br>Mrs Nicole Ashcroft<br>Wellcome Trust-MRC Institute of Metabolic Science<br>Box 289, Level 4<br>Addenbrooke's Hospital<br>Hills Road<br>Cambridge CB2 0QQ<br>UK<br>Tel: +44 (0)1223 769 068<br>Fax: +44 (0)1223 330 598<br>E-mail: nlb39@medschl.cam.ac.uk
**Support Co-ordinator**	Dr Malgorzata E Wilinska<br>Wellcome Trust-MRC Institute of Metabolic Science<br>Box 289, Level 4<br>Addenbrooke's Hospital<br>Hills Road, Cambridge CB2 0QQ<br>UK<br>Tel: +44 (0)1223 769 065<br>Fax: +44 (0)1223 330 598<br>E-mail: mew37@cam.ac.uk

**Human Factor Assessment**	Professor Fergus Cameron<br>Department of Endocrinology and Diabetes and Centre for Hormone Research,<br>Royal Children's Hospital and Murdoch Childrens Research Institute,<br>Department of Paediatrics,<br>University of Melbourne<br>Flemington Rd.,<br>Parkville, Victoria 3052<br>Australia<br>E-mail: Fergus.Cameron@rch.org.au<br><br>Associated Professor Lis Northam<br>Department of Psychology,<br>Royal Children's Hospital and Murdoch Children's Hospital<br>50 Flemington Rd<br>Parkville Victoria 3052<br>Australia<br>Tel: +61 (0) 3 9345 6523<br>Fax: +61 (0) 3 9345 5544<br>E-mail: lis.northam@rch.org.au<br><br>Professor Korey K. Hood<br>Stanford University School of Medicine<br>291 Campus Drive<br>Li Ka Shing Building<br>Stanford, CA 94305-5101<br>USA<br>Tel:+1 (650) 497-6899<br>E-mail: kkhood@stanford.edu
**Sleep Quality Assessment**	Dr Eleanor M Scott<br>Senior Lecturer in Medicine & Consultant in Diabetes and Endocrinology<br>Leeds Institute of Cardiovascular and Metabolic Medicine<br>University of Leeds<br>Leeds, LS2 9JT<br>UK<br>Tel: +44 (0)113 343 7762<br>E-mail: E.M.Scott@leeds.ac.uk
**Medical Sociologist**	Professor Julia Lawton<br>Usher Institute of Population Health Sciences & Informatics<br>The University of Edinburgh<br>Teviot Place<br>Edinburgh, EH8 9AG<br>UK<br>Tel: +44 (0)131 650 6197<br>Fax: +44 (0)131 650 6902<br>E-mail: J.Lawton@ed.ac.uk
**Social Scientist**	Dr Conor Farrington<br>THIS Institute (The Healthcare Improvement Studies Institute)<br>University of Cambridge

	Cambridge Biomedical Campus, Clifford Allbutt Building,<br>Cambridge CB2 0AH,<br>UK<br>Tel: 01223 762513<br>Email: conor.farrington@thisinstitute.cam.ac.uk
**Health Economist**	Stéphane Roze<br>Expert Health-Economist<br>HEVA HEOR Sarl<br>186 avenue Thiers<br>69006 Lyon<br>France<br>Tel: +33 (0)4 72 74 25 60<br>E-mail: SROZE@heva-heor.com
**Study Statistician**	Dr Craig Kollman<br>Jaeb Center for Health Research<br>15310 Amberly Drive, Suite 350<br>Tampa, FL 33647<br>USA<br>Tel: +1 (813) 975-8690<br>Fax: +1 (813) 903-8227<br>E-mail: ckollman@Jaeb.org
**Study Data Manager**	Mr Chris McCarthy<br>Jaeb Center for Health Research<br>15310 Amberly Drive, Suite 350<br>Tampa, FL 33647<br>USA<br>Tel: +1 (813) 975-8690<br>Fax: +1 (813) 903-8227<br>E-mail: cmccarthy@jaeb.org
**Central Clinical Laboratory**	Professor Stephen Luzio<br>Swansea University<br>Diabetes Research Unit Cymru<br>Institute of Life Science<br>3rd Floor<br>Singleton Park<br>Swansea , SA2 8PP<br>UK<br>Tel: +44 (0) 1792 602223<br>Fax: +44 (0) 1792 602225<br>E-mail: s.luzio@swansea.ac.uk

Boughton C, et al. BMJ Open 2020; 10:e033500. doi: 10.1136/bmjopen-2019-033500

**Immunological Assessment**	Professor John A. Todd<br>Professor of Medical Genetics,<br>Cambridge Institute for Medical Research,<br>JDRF/Wellcome Trust Diabetes and Inflammation Laboratory,<br>The Wellcome Centre for Human Genetics,<br>Roosevelt Drive,<br>Oxford<br>OX3 7BN<br>Tel: 01865 287854<br>E-mail: John.todd@well.ox.ac.uk	
**Study Sponsor**	Cambridge University Hospitals NHS Foundation Trust, jointly with University of Cambridge	
	Ms Carolyn Read<br>Research Governance Office<br>University of Cambridge<br>School of Clinical Medicine<br>Box 111, Cambridge Biomedical Campus<br>Cambridge, CB2 0SP<br>UK<br>Tel: +44 (0)1223 769 291<br>E-mail: researchgovernance@medschl.cam.ac.uk	Mr Stephen Kelleher<br>Cambridge University Hospitals NHS Foundation Trust<br>Box 277, Addenbrooke's Hospital<br>Hills Road<br>Cambridge, CB2 0QQ, UK<br>Tel: +44 (0)1223 217 418<br>Fax: +44 (0)1223 348 494<br>E-mail:<br>enquiries@addenbrookes.nhs.uk
**Collaborating Organization**	Medtronic MiniMed, Inc.<br>(“Medtronic”)<br>18000 Devonshire St<br>Northridge, CA 91325<br>USA	
**Funders**	Efficacy and Mechanism Evaluation Programme<br>National Institute for Health Research<br>Evaluation, Trials and Studies Coordinating Centre<br>University of Southampton<br>Alpha House, Enterprise Road<br>Southampton SO16 7NS<br>UK<br><br>The Leona M. and Harry B. Helmsley Charitable Trust<br>230 Park Avenue, Suite 659<br>New York, NY 10169<br>USA	

Data Monitoring and Ethics Committee

**Chair**<br>**Prof Timothy Jones**	Department for Endocrinology and Diabetes,<br>Princess Margaret Hospital for Children,<br>PO Box D184<br>Perth, WA 6840<br>Australia<br>Fax: +61 (0) 8 9340 8605<br>E-mail: tim.jones@health.wa.cov.au
**Expert trial statistician**<br>**Prof Chris Patterson**	Centre for Public Health,<br>Queen's University Belfast,<br>Room 3.014, ICS Block B,<br>Grosvenor Road<br>Belfast, BT12 6BJ<br>UK<br>Tel: +44 (0)28 9097 8963<br>Fax: +44 (0)28 9023 5900<br>E-mail: C.Patterson@qub.ac.uk
**Medical Expert**<br>**Dr Peter Adolfsson**	Sjukstugegatan 16,<br>målpunkt C, plan 4,<br>43480 Kungsbacka<br>Sweden<br>Tel: +46 (0)300 56 50 78<br>Email: Peter.Adolfsson@regionhalland.se

Trial Steering Committee

**Chair**<br>**Prof John Gregory**	Email: GregoryJW@cardiff.ac.uk

Boughton C, *et al. BMJ Open* 2020; 10:e033500. doi: 10.1136/bmjopen-2019-033500

PROTOCOL SIGNATURE PAGE

The signature below documents the approval of the protocol entitled “An open-label, multicentre, randomised, single-period, parallel design study to assess the effect of closed loop insulin delivery from onset of type 1 diabetes in youth on residual beta cell function compared to standard insulin therapy” version ___dated ___________and provides the necessary assurances that this study will be conducted according to all stipulations of the protocol, the principles of GCP and the appropriate reporting requirements.

Signature ……………………………………. Date……………………….

Prof Roman Hovorka, Chief Investigator

Signature ……………………………………. Date……………………….

…………………………………, **Principal Investigator**

Table of Contents

1 LIST OF ABBREVIATIONS AND RELEVANT DEFINITIONS 15
2 STUDY SYNOPSIS 18
3 SUMMARY 26
4 BACKGROUND 27
4.1 EXISTING RESEARCH 27
4.1.1 Preservation of C-peptide – evidence and approach 27
4.1.2 Closed loop technology 28
4.2 ARTIFICIAL PANCREAS RESEARCH AT CAMBRIDGE 29
4.2.1 Preclinical testing of Cambridge closed loop algorithm 29
4.2.2 Studies of closed loop in children and adolescents with type 1 diabetes in the clinical research facility 29
4.2.3 Studies of closed loop in adults with type 1 diabetes in the clinical research facility 29
4.2.4 Overnight closed loop study in children and adolescents with type 1 diabetes in home setting ... 30
4.2.5 Overnight closed loop studies in adults with type 1 diabetes in home setting 31
4.2.6 Day-and-night closed loop studies in adolescents with type 1 diabetes in home setting..... 31
4.2.7 Day and night closed loop studies in adults with type 1 diabetes in home setting 31
4.3 RISK AND BENEFITS 32
4.4 RATIONALE FOR THE CURRENT STUDY 32
4.5 AUTOMATED CLOSED LOOP SYSTEM (FLORENCEM) TO BE USED IN THE PRESENT STUDY 33
4.6 FOLLOW UP CLOSED-LOOP PLATFORM (CAMAPS FX) TO BE USED IN THE PRESENT STUDY 34
5 OBJECTIVES 35
5.1 PRIMARY OBJECTIVE 35
5.2 SECONDARY OBJECTIVES 35
5.2.1 Glucose control 35
5.2.2 Safety 35
5.2.3 Utility 35
5.2.4 Human factors 35
5.2.5 Health economics 36
5.3 EXTENSION PHASE OBJECTIVES 36
6 STUDY DESIGN 36
6.1 INTERNAL PILOT PHASE 37
6.2 FULL STUDY 37
6.3 EXTENSION PHASE 37
7 STUDY SUBJECTS 37
7.1 STUDY POPULATION 37
7.2 INCLUSION CRITERIA 41
7.3 EXCLUSION CRITERIA 41

8 METHODS UNDER INVESTIGATION 43
8.1 INTRODUCTION 43
8.2 EDUCATION FOR BOTH ARMS 43
8.3 MULTIPLE DAILY INJECTION THERAPY 43
8.4 CLOSED LOOP INTERVENTION 44
8.4.1 Name and description of the method of investigation 44
8.4.2 Intended purpose 44
8.4.3 Method of administration 44
8.4.4 Required training 44
8.4.5 Precautions 44
8.4.6 Accountability of the method under investigation 45
9 STUDY SCHEDULE 46
9.1 OVERVIEW 46
9.2 RECRUITMENT VISIT AND SCREENING ASSESSMENT (VISIT 1) 49
9.2.1 Screening and reference blood sampling 50
9.3 BASELINE VISIT (VISIT 2) 50
9.4 RUN IN PERIOD 50
9.5 RANDOMISATION 51
9.6 POST-RANDOMISATION TRAINING (VISITS 3 AND 4) 51
9.6.1 Closed loop intervention 51
9.6.2 Standard therapy (control intervention) 51
9.7 INITIATION OF TREATMENT ARM (VISIT 5) 52
9.7.1 Closed loop intervention 52
9.7.2 Standard therapy (control intervention) 52
9.8 TELEPHONE/EMAIL CONTACT AFTER INITIATION OF TREATMENT ARM 53
9.9 ROUTINE FOLLOW UP VISITS (VISIT 6, 8, 10, 11, 12) 53
9.10 FOLLOW UP VISITS INCLUDING MMTT (VISIT 7, 9) 53
9.11 PENULTIMATE VISIT 13 54
9.12 END OF STUDY VISIT (VISIT 14) 55
9.13 TRANSITION TO USUAL CARE 55
9.14 OPTIONAL EXTENSION PHASE 55
9.14.1 Routine follow up contacts 55
9.14.2 Study visits 56
9.15 PARTICIPANT WITHDRAWAL CRITERIA 56
9.16 PARTICIPANT TRANSFER 57
9.17 STUDY STOPPING CRITERIA 58
9.18 CO-ENROLMENT GUIDELINES 58
9.19 SUPPORT TELEPHONE LINE 58
9.20 STUDY PEER SUPPORT SYSTEM 58
9.21 SUBJECT REIMBURSEMENT 59

9.22 RETENTION STRATEGIES 59
10 **ENDPOINTS** 59
10.1 PRIMARY ENDPOINT 59
10.2 SECONDARY ENDPOINTS 59
10.3 SAFETY EVALUATION 61
10.4 UTILITY EVALUATION 61
10.5 HUMAN FACTORS EVALUATION 61
10.6 HEALTH ECONOMIC ASSESSMENT 61
10.7 EXTENSION PHASE OUTCOMES 61
11 **ASSESSMENT AND REPORTING OF ADVERSE EVENTS** 62
11.1 DEFINITIONS 62
11.1.1 Reportable Adverse Events 62
11.1.2 Adverse Events 62
11.1.3 Adverse Device Effect 63
11.1.4 Serious Adverse Event 63
11.1.5 Serious Adverse Device Effect 64
11.1.6 Unanticipated Serious Adverse Device Effect 64
11.1.7 Device Deficiencies 64
11.1.8 Adverse event intensity 65
11.1.9 Adverse event causality 65
11.2 RECORDING AND REPORTING OF ADVERSE EVENTS, SERIOUS ADVERSE EVENTS AND DEVICE DEFICIENCIES 66
11.2.1 Monitoring period of adverse events 66
11.2.2 Recording and reporting of adverse events 66
11.2.3 Severe hypoglycaemia 67
11.2.4 Hyperglycaemia, ketonaemia and diabetic ketoacidosis 67
11.2.5 Reporting of serious adverse events and serious adverse device effects 68
11.2.6 Recording and reporting of device deficiencies 69
11.2.7 Reporting of Pregnancy 70
11.2.8 Healthcare arrangements and compensation for adverse events 70
11.3 ANTICIPATED ADVERSE EVENTS, RISKS AND BENEFITS 70
11.3.1 Risks and anticipated adverse events 70
11.3.2 Hypoglycaemia and hyperglycaemia 70
11.3.3 Blood sampling 71
11.3.4 Finger-prick blood glucose measurements 71
11.3.5 Insulin injection therapy 71
11.3.6 Insulin pump therapy 71
11.3.7 Continuous glucose monitoring 72
11.3.8 Questionnaires, interviews and focus groups 72
11.3.9 Risk Analysis and residual risk associated with the investigational device 73

11.4 BENEFITS 73
11.5 DATA MONITORING AND ETHICS COMMITTEE (DMEC) 73
12 METHODS AND ASSESSMENTS 74
12.1 PROCEDURES 74
12.1.1 Height, weight and blood pressure 74
12.1.2 Mixed Meal Tolerance Test (MMTT) 74
12.1.3 Continuous subcutaneous glucose monitoring 74
12.1.4 Insulin pump data 75
12.2 HUMAN FACTORS ASSESSMENT 75
12.2.1 Questionnaires 75
12.2.2 Computerized cognitive testing 78
12.2.3 Measures of sleep quality 78
12.2.4 Qualitative assessment 78
12.3 HEALTH ECONOMIC EVALUATION 79
12.3.1 Simulation cohort and treatment effects 80
12.3.2 Costs and utilities 80
12.4 LABORATORY METHODS 80
12.4.1 Screening and reference sample 80
12.4.2 Lipid profile 80
12.4.3 C-peptide 81
12.4.4 Plasma glucose 81
12.4.5 HbA1c 81
12.4.6 Immunological assessment 81
12.5 BLOOD LOSS 82
13 STUDY MATERIALS AND PRODUCTS 83
13.1 INSULIN 83
13.2 MULTIPLE DAILY INSULIN INJECTIONS DURING RUN-IN AND CONTROL INTERVENTION 83
13.3 INSULIN PUMP WITH PUMP SUSPEND FEATURE 83
13.4 CONTINUOUS SUBCUTANEOUS GLUCOSE MONITOR 83
13.4.1 Blinded Continuous Subcutaneous Glucose Monitor 83
13.4.2 Real-time Continuous Subcutaneous Glucose Monitor 84
13.5 CARELINK USB LINK 84
13.6 SMARTPHONE 84
13.7 BAYER CONTOUR™ NEXT LINK BLOOD GLUCOSE METER 84
13.8 COMPUTER-BASED ALGORITHM 84
13.9 ACTIWATCH 85
14 DATA ANALYSIS 85
14.1 PRIMARY ANALYSIS 85
14.2 SECONDARY ANALYSIS 85
14.2.1 Biochemical evaluation 85

Boughton C, *et al. BMJ Open* 2020; 10:e033500. doi: 10.1136/bmjopen-2019-033500

14.2.2 Safety evaluation 85
14.2.3 Human factors evaluation 86
14.2.4 Health economics assessment 87
14.3 EVALUATIVE PERIODS 87
14.4 INTERIM MONITORING AND ANALYSES 87
14.5 STATISTICAL METHODS 87
14.6 ADHERENCE AND RETENTION 88
14.7 SAMPLE SIZE AND POWER CALCULATIONS 88
14.8 DEVIATIONS FROM THE STATISTICAL PLAN 89
15 CASE REPORT FORMS 89
16 DATA HANDLING 89
17 STUDY MANAGEMENT 90
17.1 TRIAL STEERING COMMITTEE (TSC) 90
17.2 DATA MONITORING AND ETHICS COMMITTEE (DMEC) 91
17.3 TRIAL MANAGEMENT GROUP (TMG) 91
17.4 STUDY MONITORING 91
18 RESPONSIBILITIES 91
18.1 CHIEF INVESTIGATOR 91
18.2 PRINCIPAL CLINICAL INVESTIGATORS 91
18.3 STUDY COORDINATORS 91
19 ETHICS 92
19.1 RESEARCH ETHICS COMMITTEE AND INSTITUTIONAL REVIEW BOARD 92
19.2 INFORMED CONSENT OF STUDY SUBJECTS 92
20 AMENDMENTS TO THE PROTOCOL 93
21 DEVIATIONS FROM THE PROTOCOL 93
22 TIMETABLE 93
23 REPORTS AND PUBLICATIONS 94
24 RETENTION OF STUDY DOCUMENTATION 94
25 INDEMNITY STATEMENTS 94
REFERENCES 95
26 DOCUMENT AMENDMENT HISTORY 100

1 List of abbreviations and relevant definitions

ADA	American Diabetes Association
ADE	Adverse Device Effect
ASADE	Anticipated Serious Adverse Device Effect
AE	Adverse Event
ANCOVA	Analysis of Covariance
AP	Artificial Pancreas
AR	Adverse Reaction
AUC	Area Under the Curve
BMI	Body Mass Index
CCTU	Cambridge Clinical Trials Unit
CDM	Core Diabetes Model
CE	Conformité Européenne (CE-mark)
CGM	Continuous Glucose Monitoring
CI	Chief Investigator *or* Confidence Interval
CL	Closed Loop
CRF	Case Report Form
CSII	Continuous Subcutaneous Insulin Infusion
DCCT	Diabetes Control and Complications Trial
DKA	Diabetic Ketoacidosis
DMEC	Data Monitoring and Ethics Committee
DNA	Deoxyribonucleic Acid

eCRF	Electronic Case Report Form
EudraCT	European Clinical Trial Database
FDA	US Food and Drug Administration
GCP	Good Clinical Practice
HbA1c	Glycated haemoglobin A1c
HFS	Hypoglycaemia Fear Survey
IDE	US Investigational Device Exemption
IRB	Institutional Review Board
ISPAD	International Society for Pediatric and Adolescent Diabetes
i.v.	Intravenous
MDI	Multiple Daily Injection therapy
MHRA	Medicine and Healthcare products Regulatory Agency
MMTT	Mixed Meal Tolerance Test
MPC	Model-Predictive-Control
NGP	Next Generation insulin Pump (Medtronic)
NHS	National Health Service
NICE	National Institute for Health and Care Excellence
PBMC	Peripheral Blood Mononuclear Cell
PedsQL	Pediatric Quality of Life Inventory
PI	Principal Investigator
PPI	Public and Patient Involvement
PSQI	Pittsburgh Sleep Quality Index

QALY	Quality-Adjusted Life Years
R & D	Research and Development
RCT	Randomised Controlled Trial
REC	Research Ethics Committee
RF	Radio Frequency
s.c.	Subcutaneous
SADE	Serious Adverse Device Effect
SAE	Serious Adverse Event
SAP	Sensor Augmented Pump Therapy
SD	Standard Deviation
SDQ	Strengths and Difficulties Questionnaire
T1D	Type 1 Diabetes Mellitus
TMG	Trial Management Group
TSC	Trial Steering Committee
UCPCR	Urine C-peptide/Creatinine Ratio
USADE	Unanticipated Serious Adverse Device Effect
WHO	World Health Organisation

Boughton C, *et al. BMJ Open* 2020; 10:e033500. doi: 10.1136/bmjopen-2019-033500

2 Study synopsis

**Title of clinical trial**	An open-label, multicentre, randomised, single-period, parallel design study to assess the effect of closed loop insulin delivery from onset of type 1 diabetes in youth on residual beta cell function compared to standard insulin therapy
**Short title**	Closed Loop from Onset in type 1 Diabetes (CLOuD)
**Sponsors name**	Cambridge University Hospitals NHS Foundation Trust and University of Cambridge, Cambridge, UK
**Medical condition or disease under investigation**	Type 1 diabetes
**Purpose of clinical trial**	To determine whether continued intensive metabolic control using closed loop insulin delivery (CL) following diagnosis of type 1 diabetes can preserve C-peptide secretion as a marker of residual beta cell function compared to standard multiple daily injections (MDI) therapy
**Study objectives**	<u>Primary objective:</u><br>• To assess residual C-peptide secretion 12 months after diagnosis of type 1 diabetes in participants receiving either CL insulin delivery or standard MDI therapy<br><u>Secondary Objectives:</u><br>• Biochemical:<br>o To compare effects of study interventions on residual C-peptide secretion over 24 months following diagnosis<br>o To examine how intensive diabetes management using CL insulin delivery affects glucose control in terms of safety and efficacy over 24 months<br>• Human Factors: To assess cognitive, emotional, and behavioural characteristics of participating subjects and family members and their response to closed loop insulin delivery and clinical trial

	• Health economics: To perform cost utility analysis and inform reimbursement decision-making<br>Optional extension phase will assess:<br>• Biochemical measures: residual C-peptide and glucose control in terms of safety and efficacy over a further 24 months<br>• Human Factors: retention and questionnaires
**Study design**	An open-label, multi-centre, randomised, single period, two-arm parallel group study with internal pilot, contrasting closed loop with MDI with an optional 24 month extension phase.
**Primary endpoint**	Area under the meal stimulated C-peptide curve (AUC) during a mixed meal tolerance test (MMTT) at 12 months post diagnosis
**Secondary endpoint(s)**	• Mean stimulated C-peptide AUC at baseline, 6 and 24 months<br>• Overall glucose control and glucose variability<br>o HbA1c levels<br>o Percentage of patients in each group with HbA1c <7.5% (58 mmol/mol)<br>o Percentage of time spent with sensor glucose readings in the target range (3.9 to 10mmol/l)<br>o Average, standard deviation, and coefficient of variation of sensor glucose levels<br>• Hypoglycaemia<br>o Percentage of time spent below target glucose (3.9mmol/l)*<br>o Percentage of time with sensor glucose levels <3.5 mmol/l, <3.0 mmol/l and <2.8 mmol/l<br>o AUC of sensor glucose below 3.9 mmol/l and 3.5 mmol/l<br>• Hyperglycaemia<br>o Time spent with sensor glucose above target (10.0 mmol/l)<br>o Time with sensor glucose levels in significant hyperglycaemia (glucose levels > 16.7 mmol/l)<br>• Insulin requirements

	◦ Total, basal and bolus insulin dose (U/kg)<br>• Weight<br>◦ Change in body mass index (BMI) standard deviation score<br><u>Extension Phase:</u><br>• Fasting C-peptide and glucose at 36 and 48 months<br>• Overall glucose control and glucose variability (as previously)<br>• Hypoglycaemia and hyperglycaemia (as previously)<br>• Insulin requirements (as previously)<br>• Weight (as previously)
**Exploratory endpoint(s)**	Trends in glucose control and insulin delivery, Daytime vs. overnight glucose control; relationships between CL compliance and glucose outcomes; correlation between fasting C-peptide, stimulated C-peptide and C-peptide at 90 minutes during MMTT, relationship between beta-cell function and immune markers
**Safety evaluation**	• Frequency of severe hypoglycaemic episodes as defined by International Society for Pediatric and Adolescent Diabetes (ISPAD).<br>• Frequency of diabetic ketoacidosis as defined by ISPAD<br>• Number, nature and severity of other adverse events
**Utility evaluation**	Assessment of the frequency and duration of use of the closed loop system
**Human factors assessment**	Cognitive, emotional, and behavioural characteristics of participating subjects and family members and their response to the closed loop system and clinical trial will be assessed gathering both quantitative (validated surveys and tests) and qualitative data (interviews and focus groups).
**Health economic evaluation**	Cost utility analysis on the benefits of closed loop insulin delivery to inform reimbursement decision-making
**Sample size**	96 participants randomised (48 per group); each clinical site will aim to recruit between 15 and 20 participants.

	All participants will be invited at 24 months to participate in an optional extension phase for a further 24 months.
**Summary of eligibility criteria**	Key inclusion criteria:<br>1. Diagnosis of type 1 diabetes using standard diagnostic practice within previous 21 days<br>2. Age 10 to 16.9 years<br>3. Willingness to monitor blood glucose four or more times daily<br>4. Literate in English<br>5. Willingness to wear study devices<br><br>Key exclusion criteria:<br>1. Physical or psychological condition likely to interfere with the normal conduct of the study and interpretation of the study results as judged by the investigator<br>2. Current treatment with drugs known to interfere with glucose metabolism, e.g. systemic corticosteroids, non-selective beta-blockers and MAO inhibitors<br>3. Known or suspected allergy to insulin<br>4. Regular use of acetaminophen<br>5. Lack of reliable telephone facility for contact<br>6. Pregnancy, planned pregnancy, or breast feeding<br>7. Living alone<br>8. Severe visual impairment<br>9. Severe hearing impairment<br>10. Medically documented allergy towards the adhesive (glue) of plasters<br>11. Serious skin diseases located at places of the body, which potentially are possible to be used for localisation of the glucose sensor<br>12. Illicit drugs abuse<br>13. Prescription drugs abuse<br>14. Alcohol abuse<br>15. Sickle cell disease or haemoglobinopathy<br>16. Eating disorder such as anorexia or bulimia<br>17. Milk protein allergy

**Maximum duration of study for a subject**	24 months<br>48 months if opting to participate in extension phase
**Recruitment**	Recruitment will take place at Addenbrooke's Hospital, Cambridge, Leeds Teaching Hospital, Leeds, Alder Hey Children's Hospital, Liverpool, Nottingham Hospital, Nottingham, Oxford Children's Hospital, Oxford, Southampton Children's Hospital, Southampton and Royal Hospital for Sick Children, Edinburgh.
**Consent**	Written consent/assent will be obtained from participants and/or guardians according to Research Ethics Committee (REC) requirements.<br>Additional written consent/assent will be obtained for the extension phase from participants and/or guardians according to Research Ethics Committee (REC) requirements.
**Screening and baseline assessment**	Eligible participants will undergo a screening evaluation including the following activities:<br>• medical (diabetes) history<br>• body weight, height and blood pressure measurement<br>• record of current insulin therapy<br>• screening and baseline blood sampling<br>During a baseline visit, the following assessments/ interventions will be carried out at the clinical research facility:<br>• mixed meal tolerance test (MMTT)<br>• blood sampling for lipid profile, centrally measured HbA1c, and subsequent immunological analyses<br>• questionnaires<br>• computerised cognitive testing<br>• initiating blinded CGM to assess baseline glycaemic control
**Run in period**	Following consent/screening and baseline assessment, multiple daily injection therapy will be continued in all participants. All participants will receive non study related core diabetes training as per usual clinical practice for a period of up to three weeks.

Boughton C, *et al. BMJ Open* 2020; 10:e033500. doi: 10.1136/bmjopen-2019-033500

	All subjects will be provided with 24 hour telephone helpline and will also be given written instructions about when to contact clinical team.
**Randomisation**	Eligible participants will be randomised in a 1:1 ratio using central randomisation software to either closed loop or standard therapy i.e. MDI.<br><br>Participants who opted to take part in the extension phase will continue with the study arm allocated at randomisation.
**1. Closed loop (interventional arm)**	Following randomisation, participants in the closed loop group will receive additional training sessions to cover key aspects of insulin pump use and CGM, prior to starting closed loop insulin delivery.<br><br>Once competent in the use of the study pump and CGM system, participants will receive training required for safe and effective use of the closed loop system. During a 2-4 hour session participants will operate the system under the supervision of the clinical team. Competency on the use of closed loop system will be evaluated. Thereafter, participants are expected to use closed loop for 24 months without supervision or remote monitoring. The 24 hour support helpline will be available in case of problems.
**2. Multiple daily injections (control arm)**	Participants in the control group will receive additional training sessions following randomisation including a refresher on carbohydrate counting skills, and insulin dose adjustments.<br><br>Standard therapy (i.e. MDI) will be applied for 24 months. Participants will be allowed to switch to insulin pump therapy if clinically indicated.
**Follow up assessments**<br><br>**(3-, 6-, 9-, 12-, 15-, 18-, 21- months)**	*Both arms*. Follow up study visits will be conducted 3 monthly including data downloads/recording of insulin requirements, adverse event recording, and blood sampling (HbA1c).<br><br>Participants will be fitted with blinded CGM systems at the end of each follow up visit. The sensors will be worn at home for up to 14 days and will be sent back to the research team.<br><br>MMTTs will be performed at 6 month and 12 month follow up visits.<br><br>Sleep will be assessed using a wristwatch device for 7 days following study visits at 6 and 12 months post diagnosis. Concomitantly, a sleep diary and sleep quality questionnaire will be distributed.<br><br>Validated questionnaires evaluating the impact of the technology on quality of life, life change,

	diabetes management and fear of hypoglycaemia will be completed at the 12 month visit.<br>At 12 months, participants will repeat the computerised cognitive tests first administered at baseline.<br>Qualitative interviews will be conducted at month 12 in a subset of subjects and parents in the closed loop arm.
**End of study assessments (24 months)**	A MMTT will be performed.<br>A blood sample will be taken for measurement of HbA1c, lipids and immunological analyses.<br>Validated questionnaires evaluating the impact of the technology on quality of life, life change, diabetes management and fear of hypoglycaemia will be completed.<br>Participants will repeat the computerised cognitive tests first administered at baseline.<br>Sleep will be assessed using a wristwatch device for 7 days within the last month of the trial. Concomitantly, a sleep diary and sleep quality questionnaire will be distributed.<br>Participants and families will be invited to attend focus group discussions.
**Extension Phase**	*Both arms.* Follow up contacts will be conducted 3 monthly including recording of adverse events, medical history and insulin requirements.<br>At 36 and 48 month follow-up visits, blood sampling for fasting C-peptide and glucose and HbA1c will be undertaken and participants will be fitted with blinded CGM sensors at the end of the visit. The sensors will be worn at home for up to 14 days and sent back to the research team.<br>Validated questionnaires evaluating the impact of the technology on quality of life, life change, diabetes management, sleep quality and fear of hypoglycaemia will be completed at the 36 and 48 months.
**24-hour telephone helpline**	In case of any technical device or problems related to diabetes management such as hypo- or hyperglycaemia, subjects will be able to contact a 24-hour telephone helpline to the local clinical and research team at any time. The local research team will have access to central 24 hour advice on technical issues.

**Procedures for safety monitoring during trial**	Standard operating procedures for monitoring and reporting of all adverse events (AE) will be in place, including serious adverse events (SAE), serious adverse device effects (SADE) and specific adverse events such as severe hypoglycaemia.<br>Subjects will be asked to test and record blood or urine ketones if their finger prick glucose is above 14.0 mmol/l, as part of the safety assessment for hyperglycaemia.<br>A data monitoring and ethics committee (DMEC) will be informed of all serious adverse events and any unanticipated serious adverse device effects that occur during the study and will review compiled adverse event data at periodic intervals.
**Criteria for withdrawal of patients on safety grounds**	A subject, parent, or guardian may terminate participation in the study at any time without necessarily giving a reason and without any personal disadvantage. An investigator can stop the participation of a subject after consideration of the benefit/risk ratio. Possible reasons are:<br>1. Serious adverse events<br>2. Significant protocol violation or non-compliance<br>3. Failure to satisfy competency assessment<br>4. Decision by the investigator, or the Sponsor, that termination is in the subject's best medical interest<br>5. Pregnancy, planned pregnancy, or breast feeding<br>6. Allergic reaction to insulin<br>Efforts will be made to retain subjects in follow up for the final primary outcome assessment even if the intervention is discontinued, unless the investigator believes that it will be harmful for the subject to continue in the trial.

Boughton C, *et al. BMJ Open* 2020; 10:e033500. doi: 10.1136/bmjopen-2019-033500

3 Summary

The purpose of the study is to use a novel treatment approach, the artificial pancreas, after diagnosis of type 1 diabetes (T1D) to improve glucose control with the anticipated improvements of residual C-peptide secretion.

This is an open-label, multi-centre, single-period, randomised, parallel group design study. It is expected that a total of up to 190 subjects (aiming for 96 randomised subjects) will be recruited within 21 days of diagnosis of type 1 diabetes through paediatric diabetes centres in the UK. Half of the participants aged 10 to 16.9 years will be treated by conventional insulin injections and the other half by the artificial pancreas (closed loop insulin delivery system). Each treatment will last 24 months. All participants completing the 24 month study period will be invited to continue in an optional extension phase with the treatment allocated at randomisation for a further 24 months.

Subjects in the intervention group will receive additional training on components of the artificial pancreas, i.e. insulin pump and continuous glucose monitoring (CGM), prior to starting closed loop insulin delivery. Subjects in the control intervention group will continue with standard therapy, i.e. multiple daily injection therapy. The study includes up to 14 visits and 1 telephone/email contact for subjects completing the study. After run-in and randomisation, visits will be conducted every 3 months in both arms. Beta-cell function will be assessed by serial measurement of C-peptide in response to a standardised mixed meal tolerance test (MMTT). MMTTs will be conducted at baseline, 6-,12- and 24 months post diagnosis.

The primary outcome is the between group difference in the area under the stimulated C-peptide curve (AUC) of the MMTT at 12 month post diagnosis. Secondary outcomes include between group differences in stimulated C-peptide AUC over 24 months, differences in glycaemic control as assessed by HbA1c, time spent in glucose target range, glucose variability, hypo- and hyperglycaemia as recorded by periodically applied CGM, as well as insulin requirements and change in bodyweight. Additionally, cognitive, emotional and behavioural characteristics of participating subjects and parents will be assessed, and a cost utility analysis on the benefits of closed loop insulin delivery will be performed. Safety evaluation comprises assessment of the frequency of severe hypoglycaemic episodes, diabetic ketoacidosis (DKA) and number, nature and severity of other adverse events.

During the extension phase, participants in both arms will have follow-up contacts every 3 months. Beta-cell function will be assessed by measurement of fasting C-peptide and glucose at 36 and 48 months post-diagnosis along with measures of glucose control and safety and utility evaluation.

4 Background

4.1 Existing research

Management of newly diagnosed T1D in children and adolescents is challenging for patients, families, carers, and health care professionals. Glucose is the dominant metabolic substrate for brain function (1) and the glycaemic instability inherent in TID is known to affect brain structure and function in those with poorly controlled disease (2). Severe hypoglycaemia, particularly nocturnal episodes, is more common in children (3) and has a negative impact on the developing brain (2; 4). Fear of hypoglycaemia is common (5), impacts quality of life and psychological well-being of the young and their families (6), and leads to suboptimal glucose control (6). Glycaemic control usually deteriorates during adolescence. The Diabetes Control and Complications Trial (DCCT) revealed both higher HbA1c levels and a 50% increase in the rate of severe hypoglycaemia in intensively treated adolescents compared to adults (7). Teenagers with T1D face the burden of diabetes management in addition to major physiological and psychological changes accompanying puberty.

4.1.1 Preservation of C-peptide – evidence and approach

At the clinical diagnosis of diabetes most patients have residual pancreatic islet cells which can continue to secrete insulin for several additional years. In the DCCT (7), 35% of participants with diabetes duration of 1-5 years had persistent islet cell function (meal stimulated C-peptide levels of 0.2 to 0.5 pmol/ml). Assignment to the intensively managed group reduced the risk for loss of C-peptide by 57% over the mean 6.5 years of study. This was very clear proof that metabolic control had a significant effect on preservation of islet cell function. However, intensification of insulin therapy inevitably hits the barrier of hypoglycaemia (8). Four in five youth aged 13 to 18 years fails to meet the International Society for Pediatric and Adolescent Diabetes (ISPAD) glycaemic control target of HbA1c below 7.5% (58.5 mmol/mol) (9).

In the DCCT, those who had greater or equal 0.20 pmol/ml C-peptide initially or sustained over a year had markedly less complications – a 79% decrease in the relative risk of retinopathy (10). Importantly, these benefits were seen in the face of less hypoglycaemia events. Individuals in the intensive treated group with ≥0.20 pmol/ml C-peptide had about the same frequency of severe hypoglycaemia as those in the standard care group; a 30% reduction as compared to those in intensive therapy without this level of C-peptide. A more recent analysis found a linear relationship between frequency of retinopathy progression and C-peptide as low as 0.03 pmol/ml (11).

Boughton C, *et al. BMJ Open* 2020; 10:e033500. doi: 10.1136/bmjopen-2019-033500

Islet transplant studies have also shown that even small amounts of residual beta-cell function are clinically important. Vantyghem et al. showed that while significant beta-cell function was required to improve mean glucose, lower glucose excursions, and result in insulin independence, participants who maintained minimal beta-cell function experienced almost no severe hypoglycaemic events (12).

Metabolic control following the onset of type 1 diabetes can have a major impact on preserving residual islet cell function. Two weeks of islet cell rest after clinical diagnosis of diabetes resulted in stimulated C-peptide levels 1 year post diagnosis of 0.51 pmol/ml (13), greater than that seen after a year of cyclosporine treatment (peak C-peptide of 0.45 pmol/ml (14). However, a short four day burst of tight glucose control failed to improve meal stimulated C-peptide secretion 12 months post diagnosis (15).

4.1.2 Closed loop technology

The emergence of new technologies including continuous glucose monitoring (16), sensor augmented pump therapy (SAP) (17), and threshold pump suspend (18; 19) provides new opportunities to improve outcomes. The most promising approach is closed loop insulin therapy (20) which combines real-time continuous glucose monitoring with insulin pump therapy to achieve glucose responsive subcutaneous insulin delivery mimicking beta-cell function. The vital component of such a system, also known as an artificial pancreas (AP), is a computer-based algorithm. The role of the control algorithm is to translate, in real-time, the information it receives from the CGM and to compute the amount of insulin to be delivered by the pump. The other components include a real-time continuous glucose monitor and an infusion pump to titrate and deliver insulin. Timely insulin delivery is expected to rest the beta-cells and lessen the immune attack.

The closed loop approach has been successfully evaluated in children and adolescents in controlled laboratory studies (21-23) and in home settings (24-27). Investigations in adults have also been conducted (25; 28; 29). The results demonstrated improved glucose control and reduced risk of hypoglycaemia events. Psychosocial assessments supported acceptability and positive impact of this novel therapeutic approach among children/adolescents and carers (30), although the potential benefit in preserving cognitive function is, as yet, unknown. The closed loop approach promises to transform management of type 1 providing a tangible option to improve residual beta-cell function.

Boughton C, *et al. BMJ Open* 2020; 10:e033500. doi: 10.1136/bmjopen-2019-033500

4.2 Artificial Pancreas research at Cambridge

4.2.1 Preclinical testing of Cambridge closed loop algorithm

The research conducted at the University of Cambridge focused on developing a closed loop system for overnight glucose (initial approach) and day-and-night control (more recent applications; see below) in subjects with T1D. Studies that have been performed employed model predictive control (MPC) – this algorithm estimates user-specific parameters from CGM measurements taken every 1 to 15 minutes and makes predictions of glucose excursions, which are then used to direct insulin infusion between meals and overnight whilst standard bolus calculator is used to deliver prandial insulin (31).

The MPC algorithm has been studied extensively using *in silico* testing utilising a simulator developed by members of the study team (32). The simulations suggested a reduced risk of nocturnal hypoglycaemia and hyperglycaemia with the use of the MPC algorithm (33).

4.2.2 Studies of closed loop in children and adolescents with type 1 diabetes in the clinical research facility

To date around sixty children and adolescents with type 1 diabetes have been studied at the clinical research facility. Closed loop insulin delivery was maintained on more than 100 nights. No episodes of significant hypoglycaemia (plasma glucose concentration less than 2.8 mmol/l) have been observed thus far during closed loop blood glucose control. Results from these studies were published in The Lancet (21) and showed that overnight closed loop therapy increased the time spent euglycaemic by 37% and reduced the risk of overnight hypoglycaemia eight-fold, as compared to conventional pump treatment. Different real-life scenarios predisposing to nocturnal hypoglycaemia, such as afternoon exercise, were explored and closed loop therapy reduced the risk of overnight hypoglycaemia as compared to conventional insulin pump therapy in a randomised, cross-over design.

4.2.3 Studies of closed loop in adults with type 1 diabetes in the clinical research facility

We have completed two randomised overnight closed loop studies in 24 adults with T1D, testing a similar closed loop system comprising CGM and pump devices and the MPC algorithm. The first study (n=12) assessed the feasibility and efficacy of overnight closed loop insulin delivery following a moderate-sized (60g carbohydrate) evening meal compared with conventional pump therapy. We demonstrated that overnight closed loop insulin delivery, compared with usual continuous subcutaneous insulin infusion (CSII), significantly increased time in target plasma glucose range

(3.9-8 mmol/l) by 24% and reduced glycaemic variability as measured by standard deviation of plasma glucose. The improvements in glucose control seen on closed loop were even greater after midnight, when time in target increased by 41%. In the second study we tested the efficacy of overnight closed loop following a common situation such as consuming a large (100g carbohydrate) evening meal and drinking alcohol (0.75g ethanol/kg body weight of 13%abv white wine). We showed that overnight closed loop insulin delivery, compared with conventional CSII, similarly increased time in target plasma glucose between 3.9 and 8.0 mmol/l by 24% and reduced time spent above target by 11%, even following such challenges. Importantly these improvements during closed loop were achieved with no increased requirement in the average rate of insulin infusion overnight. These results have been published in the British Medical Journal (34).

4.2.4 Overnight closed loop study in children and adolescents with type 1 diabetes in home setting

Following successful demonstration of safety and efficacy of closed loop insulin delivery in the research facility, overnight closed loop studies under free living conditions were commenced in July 2012. The first study compared the efficacy and safety of closed loop with sensor augmented pump therapy in 16 adolescents over a three week duration (24). Closed loop was activated over at least 4 hours on 269 nights (80%); sensor data were collected over at least 4 hours on 282 control nights (84%). Closed loop increased the time when glucose was in target range by a median 15% (interquartile range −9 to +43), P<0.001. Mean overnight glucose was reduced by a mean 0.8±3.2 mmol/l, P<0.001. Time when glucose was below 3.9 mmol/l was low in both groups but nights with glucose below 3.5mmol/l for at least 20min were less frequent during closed loop (10% vs. 17%, P=0.01). Despite lower total daily insulin doses by a median 2.3 (interquartile range -4.7 to +9.3) units, P=0.009, overall 24h glucose was reduced by a mean 0.5 (standard deviation 2.3 mmol/l (P=0.006) during closed loop.

In a second multicentre, crossover, randomised, controlled study, we compared 12 week use of an overnight closed loop insulin delivery system with sensor augmented pump therapy in children and adolescents aged 6 to 18 years (25). The proportion of time with the night-time glucose level in the target range (3.9 to 8.0 mmol/l) was higher during the closed loop phase than during the control phase (by 24.7 percentage points; 95% CI, 20.6 to 28.7; P<0.001), and the mean night-time glucose level was lower (difference, −1.6 mmol/l; 95% CI,−2.2 to −1.1; P<0.001). The area under the curve for the period in which the day-and-night glucose levels were less than 3.5 mmol/l was lower by 42% (95% CI, 4 to 65; P = 0.03). Two severe hypoglycaemic episodes occurred during the closed loop phase when the closed loop system was not in use.

4.2.5 Overnight closed loop studies in adults with type 1 diabetes in home setting

A four week overnight closed loop study under free living conditions in 24 adults with type 1 diabetes on insulin pump therapy in a multicentre crossover study design was completed in 2014 (29). Closed loop was utilised over median 8.3 (interquartile range 6.0, 9.6) hours on 555 nights (86%). The proportion of time when overnight glucose was in the overnight target range between 3.9 and 8.0 mmol/l from midnight to 07:00 was significantly higher during closed loop compared to sensor augmented pump therapy (52.6%±10.6 vs. 39.1%±12.8, mean±SD; p<0.001). Mean overnight glucose (8.2±0.9 vs. 9.0±1.3 mmol/l, p=0.005) and time spent above target (44.3%±11.9 vs. 57.1%±15.6, p=0.001) were significantly lower during closed loop. Time spent below target was low and comparable between interventions [1.8%(0.6, 3.6) vs. 2.1%(0.7, 3.9), p=0.28].

4.2.6 Day-and-night closed loop studies in adolescents with type 1 diabetes in home setting

We completed a randomised, crossover design study in adolescents aged 10 to 18 years who underwent two 7-day home periods of sensor-augmented insulin pump therapy or closed loop insulin delivery without supervision or remote monitoring (27). The proportion of time when the sensor glucose level was in the target range (3.9–10 mmol/L) was increased during closed loop insulin delivery compared with sensor-augmented pump therapy (72% vs. 53%, P < 0.001; primary end point), the mean glucose concentration was lowered (8.7 vs. 10.1 mmol/L, P = 0.028), and the time spent above the target level was reduced (P = 0.005) without changing the total daily insulin amount (P = 0.55). The time spent in the hypoglycaemic range was low and comparable between interventions. A three week single centre study in children and adolescents has also been completed (N = 12).

4.2.7 Day and night closed loop studies in adults with type 1 diabetes in home setting

In 2014, we completed a first study testing a day and night home system over a seven day period in 17 adults. This randomised clinical trial adopted a multicentre, multi-national, crossover design. During the home phase, the percentage time when glucose was in target range (3.9 to 10.0 mmol/l) was significantly higher during closed loop compared to sensor augmented pump therapy (75 [61, 79] vs. 62 [53, 70]%, median [IQR], p=0.005). Mean glucose (8.1 vs. 8.8 mmol/l, p=0.027) and time spent above target (p=0.013) were lower during closed loop while time spent below target was comparable (p=0.339). Increased time in target was observed during both day-time (p=0.017) and night-time (p=0.013).

We completed a multicentre, multinational, crossover, randomised, controlled study under free living home conditions comparing 24/7 closed loop insulin delivery with sensor augmented pump therapy

(control intervention) in 33 adults with type 1 diabetes (25). The proportion of time that the glucose level was in the target range (3.9 to 10.0 mmol/l) was 11.0 percentage points (95% confidence interval [CI], 8.1 to 13.8) greater with the use of the closed loop system day and night than with control therapy (P<0.001). The mean glucose level was lower during the closed loop phase than during the control phase (difference, −0.6 mmol/l; 95% CI, −0.9 to −0.3; P<0.001), as were the area under the curve for the period when the glucose level was less than 3.5 mmol/l (39% lower; 95% CI, 24 to 51; P<0.001) and the mean glycated hemoglobin level (difference, −0.3%; 95% CI, −0.5 to −0.1; P = 0.002).

4.3 Risk and benefits

A potential key benefit of closed loop insulin delivery is the retention of residual C-peptide secretion which has been shown to decrease the risk of microvascular complications and a lower risk of severe hypoglycaemia by 65% when compared to intensively treated participants without residual beta-cell function in the DCCT trial (7). Thus the most important long term impact of improved glucose control and residual islet function may be reduced rates of diabetes complications and improved quality of life.

Any potential risks presented by this investigation have been minimized and adequate testing, safeguards, and safety monitoring will be incorporated into the investigation to further minimize and mitigate these risks. A detailed Risk Management File adopting risk management processes complying with EN ISO 14971:2012 Medical Devices – Application of Risk Management to Medical Devices, will be submitted as part of the regulatory submission to the MHRA.

4.4 Rationale for the current study

The study builds on recent technological advances of closed loop insulin delivery (artificial pancreas). The purpose of this study is to test the impact of continued intensive metabolic control using closed loop insulin delivery after diagnosis on preservation of C-peptide residual secretion. The study enrols children aged 10 and older, as they are characterised by higher residual C-peptide secretion at diagnosis compared to younger children. The present study will also test the feasibility and acceptance of this therapy so that it could be considered as a standard treatment modality in the future.

Boughton C, *et al. BMJ Open* 2020; 10:e033500. doi: 10.1136/bmjopen-2019-033500

We propose an internal pilot to test the feasibility of recruiting a patient cohort within a predetermined time period.

The extension phase will allow ongoing assessment of the impact of continued intensive metabolic control using closed-loop insulin delivery on residual C-peptide and will test acceptability of this therapy over a longer duration.

4.5 Automated closed loop system (FlorenceM) to be used in the present study

The automated closed loop system (FlorenceM) will consist of:

- Next generation sensor augmented Medtronic insulin pump 640G (Medtronic Minimed, CA, USA) incorporating the Medtronic Enlite 3 family real time CGM and glucose suspend feature

- An Android smartphone containing the Cambridge model predictive algorithm and communicating wirelessly with the insulin pump using a proprietary translator device.

An overview of this proposed automated closed loop system is given in Figure 1.

Figure 1: Representative design of the proposed FlorenceM automated closed loop system

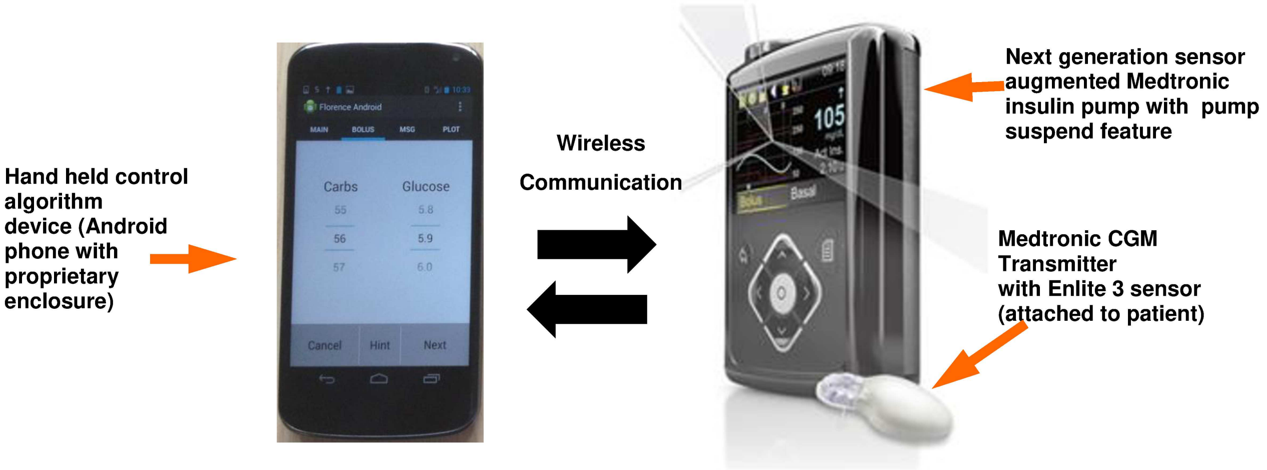

Boughton C, *et al. BMJ Open* 2020; 10:e033500. doi: 10.1136/bmjopen-2019-033500

4.6 Follow up closed-loop platform (CamAPS FX) to be used in the present study

Participants in the closed-loop arm can use the follow up closed-loop platform any time after recruitment into the study. The automated closed-loop platform (CamAPS FX) will consist of:

- Dana insulin pump (Diabecare, Sooil, Seoul, South Korea)
- Dexcom G6 real-time CGM sensor (Dexcom, Northridge, CA, USA)
- An Android smartphone hosting CamAPS FX Application with the Cambridge model predictive control algorithm and communicating wirelessly with the insulin pump
- Cloud upload system to monitor CGM/insulin data.

An overview of this proposed automated closed loop platform is given in Figure 1A.

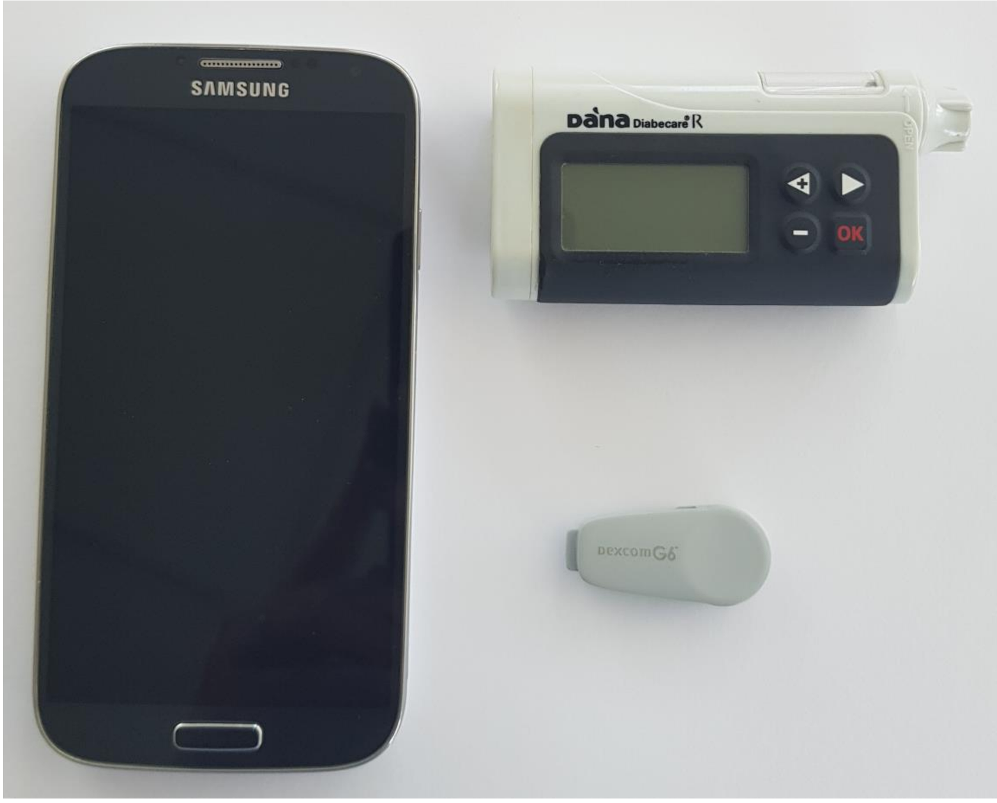

Figure 1A CamAPS FX comprises Samsung Galaxy phone (or similar) running Cambridge control algorithm, Dana insulin pump (Sooil), G6 real-time CGM sensor (Dexcom).

5 Objectives

5.1 Primary objective

The primary objective is to evaluate the effect of continued intensive metabolic control using closed loop insulin delivery after diagnosis on preservation of C-peptide residual secretion by comparing the area under the stimulated C-peptide curve (AUC) of a mixed meal glucose tolerance test conducted at the 12 month visit in participants receiving closed loop insulin delivery with those receiving standard therapy, i.e. multiple daily injections applying basal bolus regimen.

The objective of an internal pilot phase is to carry out preliminary evaluation of recruitment, randomisation, treatment and follow-up assessments at the five participating sites.

5.2 Secondary objectives

5.2.1 Glucose control

The objective is to examine the efficacy of day-and-night closed loop compared with standard basal bolus regimen as far as glucose control is concerned. We will compare between group differences in HbA1c, and parameters based on subcutaneous continuous glucose monitoring (CGM), such as the percentage of time spent within, below and above the target range from 3.9 to 10.0 mmol/l.

5.2.2 Safety

The objective is to evaluate the safety of day and night automated closed loop glucose control in terms of episodes of severe hypoglycaemia and other adverse events.

5.2.3 Utility

The objective is to determine the frequency and duration of use of the automated closed loop system.

5.2.4 Human factors

The objective is to assess cognitive, emotional, and behavioural characteristics of participating subjects and family members and their response to the closed loop system and clinical trial in order to aid interpretation of trial results and inform recommendations for future use of closed loop systems.

Boughton C, *et al. BMJ Open* 2020; 10:e033500. doi: 10.1136/bmjopen-2019-033500

5.2.5 Health economics

A cost utility analysis on the benefits of closed loop insulin delivery will be conducted to inform reimbursement decision-making.

5.3 Extension Phase objectives

The objective of the extension phase is to evaluate the effect of continued intensive metabolic control using closed loop insulin delivery after diagnosis on preservation of C-peptide residual secretion by comparing the fasting C-peptide and glucose levels conducted at the 36 and 48 month visit in participants receiving closed loop insulin delivery with those receiving standard therapy, i.e. multiple daily injections.

The extension phase will also examine the efficacy of day-and-night closed loop compared with standard basal bolus regimen on glucose control comparing between group differences in HbA1c, and parameters based on subcutaneous continuous glucose monitoring (CGM).

The safety of day and night automated closed loop glucose control will be evaluated in terms of episodes of severe hypoglycaemia and other adverse events.

The frequency and duration of use of the automated closed loop system will be assessed.

Emotional and behavioural characteristics of participating subjects and family members and their response to the closed loop system and clinical trial will be evaluated using questionnaires.

6 Study design

This will be an open-label, multi-centre, randomised, single-period, two-arm parallel group study with internal pilot phase, contrasting automated closed loop glucose control (CL) with multiple daily injections (MDI).

At diagnosis of T1D young people and families will be invited to participate in the study. Eligible participants will be randomised in a 1:1 ratio using central randomisation software to either closed loop (CL) or multiple daily injection therapy (MDI). The study will aim for a total of 96 (15-20 per site) completed participants. Recruitment will target up to 190 to allow for dropouts. Subjects who have signed the consent/assent but drop out during run-in may be replaced. The study flow chart is outlined in Figure 2.

There will be an optional 24 month extension phase for participants in both study arms to continue with the treatment allocated at randomisation.

6.1 Internal pilot phase

The purpose of the internal pilot study is to estimate the rate of recruitment, and to pilot randomisation, treatment and follow-up assessments at the five participating sites. During the pilot phase, we aim to recruit at least 10 subjects i.e. 2 per site. All participants recruited during the pilot phase will proceed to the full study.

6.2 Full study

Following the internal pilot phase and consecutive re-evaluation of recruitment procedures and follow-up assessments, recruitment for the study will be resumed at full rate, and all 96 randomised subjects will be followed up until study completion.

6.3 Extension phase

At 24 months, all participants will be invited to participate in an optional extension phase to continue with their current treatment (automated closed loop glucose control or multiple daily injections (MDI) for a further 24 months.

Permission will be sought from participants and their carers for ongoing submission of routine clinical data to the research team for a further nine years to enable long term outcomes to be reported.

7 Study subjects

7.1 Study population

This is a multicentre study and recruitment will take place at the following centres:

1. Addenbrooke’s Hospital, Cambridge, UK
2. Leeds Teaching Hospital, Leeds, UK
3. Alder Hey Children’s Hospital, Liverpool, UK
4. Nottingham Hospital, Nottingham, UK
5. Oxford Children's Hospital, Oxford, UK
6. Southampton Children’s Hospital, Southampton, UK
7. Royal Hospital for Children and Yong People, Edinburgh, UK

Boughton C, *et al. BMJ Open* 2020; 10:e033500. doi: 10.1136/bmjopen-2019-033500

Up to a total of 96 youths aged 10 to 16.9 years will be recruited within 21 days of diagnosis of type 1 diabetes. Each site will aim to recruit between 15 to 20 participants. Participants may also be recruited from other diabetes centres (Patient Identification Centres) in the East Anglia region and London for the Addenbrooke's site, from the Thames Valley region for the Oxford Children's Hospital site and from the Wessex Paediatric Network for the Southampton Children's Hospital site.

Potential participants will be identified by their treating clinicians and a contact with the research team will be established if agreed. Study information leaflets and/or similar recruitment material will be handed out or sent to participants by the research team including an invitation to join the study. Written informed consent will be obtained from all participants aged 16 years and parents/guardians of participants aged 15 years and younger, before any study related activities. Participants aged 15 years and younger will be asked to provide evidence of their assent to the study procedures.

At 24 months, all participants opting to continue with the extension phase of the study will be asked to re-consent. Written informed consent will be obtained from participants aged 16 years and parents/guardians of participants aged 15 years and younger, before any study related activities. Participants aged 15 years and younger will be asked to provide evidence of their assent to the study procedures.

Boughton C, *et al. BMJ Open* 2020; 10:e033500. doi: 10.1136/bmjopen-2019-033500

Figure 2: Study flow chart

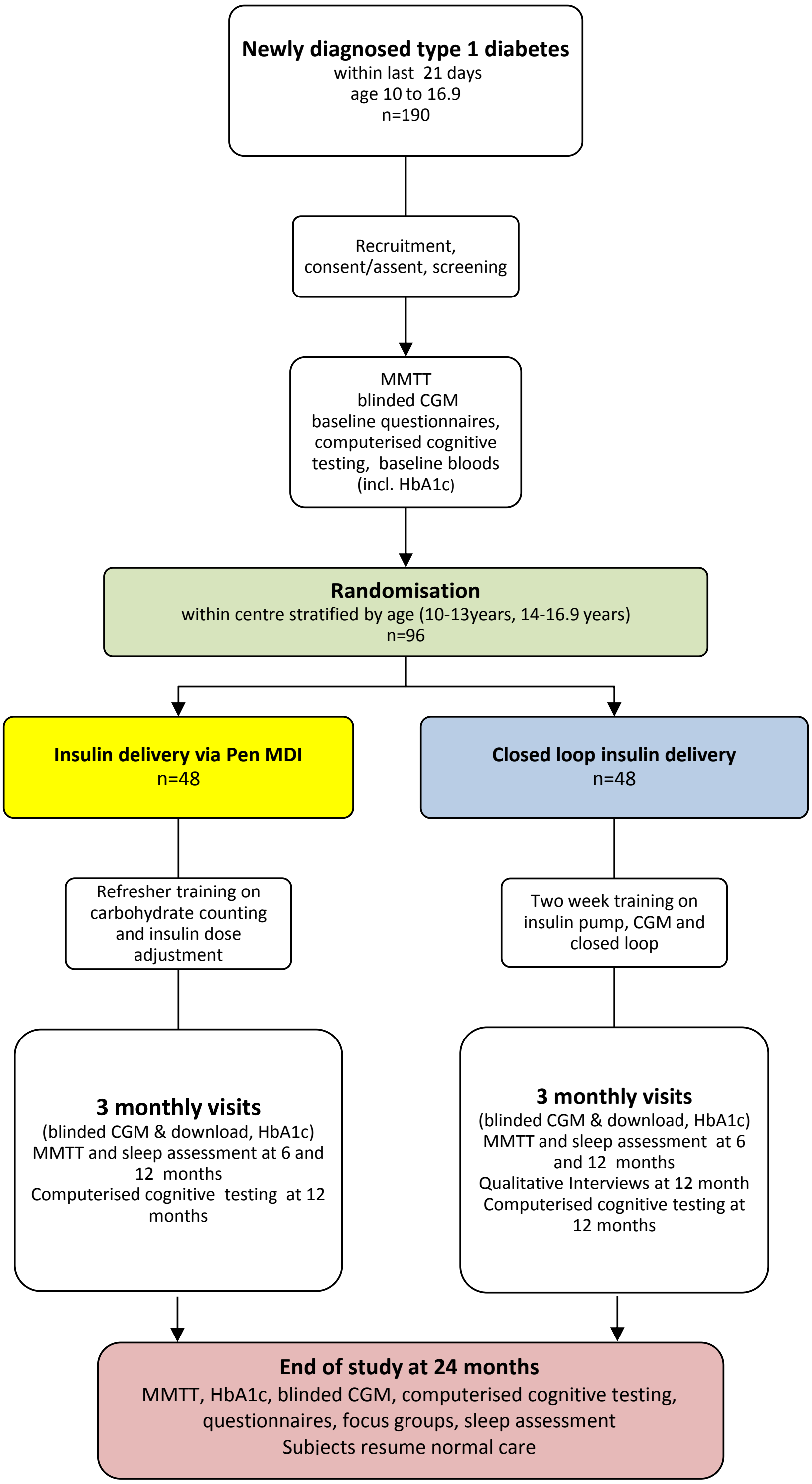

Figure 3: Study flow chart for extension phase

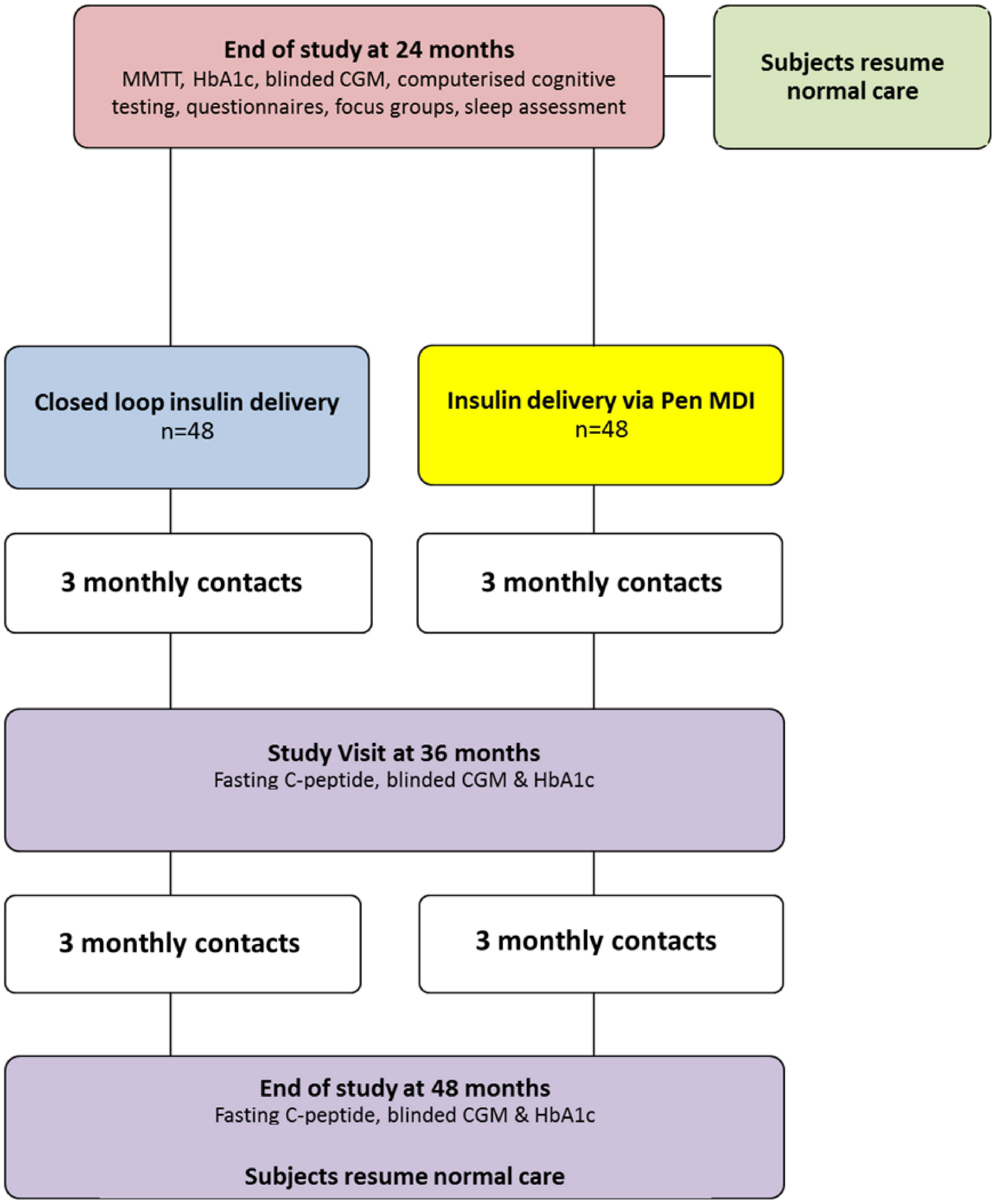

7.2 Inclusion criteria

1. Diagnosis of type 1 diabetes within previous 21 days. Day 1 will be defined as the day insulin was first administered. Type 1 diabetes will be defined according to WHO criteria using standard diagnostic practice.

   [WHO definition: 'The aetiological type named type 1 encompasses the majority of cases with are primarily due to beta-cell destruction, and are prone to ketoacidosis. Type 1 includes those cases attributable to an autoimmune process, as well as those with beta-cell destruction for which neither an aetiology nor a pathogenesis is known (idiopathic). It does not include those forms of beta-cell destruction or failure to which specific causes can be assigned (e.g. cystic fibrosis, mitochondrial defects, etc.).']
2. The subject is at least 10 years and not older than 16.9 years
3. The subject/carer is willing to perform regular capillary blood glucose monitoring, with at least 4 blood glucose measurements taken every day
4. The subject is literate in English
5. The subject is willing to wear glucose sensor
6. The subject is willing to wear closed loop system at home
7. The subject is willing to follow study specific instructions
8. The subject is willing to upload pump and CGM data at regular intervals

7.3 Exclusion criteria

1. Physical or psychological condition likely to interfere with the normal conduct of the study and interpretation of the study results as judged by the investigator
2. Current treatment with drugs known to interfere with glucose metabolism, e.g. systemic corticosteroids, non-selective beta-blockers and MAO inhibitors etc.
3. Known or suspected allergy to insulin
4. Regular use of acetaminophen
5. Lack of reliable telephone facility for contact
6. Pregnancy, planned pregnancy, or breast feeding
7. Living alone
8. Severe visual impairment
9. Severe hearing impairment

10. Medically documented allergy towards the adhesive (glue) of plasters or unable to tolerate tape adhesive in the area of sensor placement
11. Serious skin diseases (e.g. psoriasis vulgaris, bacterial skin diseases) located at places of the body, which potentially are possible to be used for localisation of the glucose sensor
12. Illicit drugs abuse
13. Prescription drugs abuse
14. Alcohol abuse
15. Sickle cell disease, haemoglobinopathy, receiving red blood cell transfusion or erythropoietin within 3 months prior to time of screening
16. Eating disorder such as anorexia or bulimia
17. Milk protein allergy

8 Methods under investigation

8.1 Introduction

This randomised controlled trial (RCT) compares two methods of insulin delivery in children from 10 to 16.9 years of age at diagnosis of type 1 diabetes. During run-in period and control intervention when standard multiple daily injection (MDI) therapy will be applied, CE-marked insulin pens will be used as per usual clinical practice. For participants randomised to closed loop intervention, insulin will be applied by an investigational closed loop medical device, see sections 4.5. and 8.4. During run-in and both interventions, rapid acting insulin analogues will be used (insulin aspart, insulin lispro, insulin glulisine or similar or ultra-rapid insulin analogue). When applying MDI, long acting insulin analogues will be used (insulin glargine, insulin detemir or similar) as described in section 13.1.

8.2 Education for both arms

At entry to the study, all participants will complete a structured educational programme delivered to the participants and their families in accordance with the standards of the International Society for Pediatric and Adolescent Diabetes (35). All participants will be trained on the use of MDI regimen.

Participants and their families will be educated in:

- Type 1 diabetes
- The use and administration of insulin
- Hyperglycaemia and correction doses
- Hypoglycaemia symptoms and treatment
- Exercise
- Sick day rules
- Carbohydrate counting and dietetic education
- The benefits of maintaining optimal glycaemic control for long term health
- Blood glucose monitoring

8.3 Multiple daily injection therapy

Rapid acting insulin analogue and long acting insulin analogue (see 13.1) will be subcutaneously administered using CE-marked insulin pen devices in accordance with the manufacturer's instructions for their intended purposes.

Boughton C, *et al. BMJ Open* 2020; 10:e033500. doi: 10.1136/bmjopen-2019-033500

Participants will be given long acting analogue once or twice daily according to their needs and boluses of rapid acting analogue when carbohydrates are consumed.

Participants will be managed as per local treatment protocols. Guidelines for health care professionals with respect to starting dose calculations and dose modifications will be provided separately in the study manual.

Pens/consumables for participants allocated to MDI will be supplied in line with normal clinical practice.

8.4 Closed loop intervention

8.4.1 Name and description of the method of investigation

The investigational treatment is the FlorenceM, see section 4.5 or follow up prototypes of the automated day and night closed loop system manufactured by the Cambridge University Hospitals NHS Foundation Trust and supported by Medtronic Minimed Inc. Northridge, CA, USA. Component versions will be identified during regulatory submission to the MHRA. CamAPS FX is a follow up closed-loop platform which will be used in the study by participants, see section 4.6.

8.4.2 Intended purpose

The intended purpose of the investigational treatment is automated day and night hybrid closed loop insulin delivery with or without pump suspend feature.

8.4.3 Method of administration

The closed loop system consists of components directly attached to the patient, which are the CGM transmitter and the insulin pump. The component not directly attached to the patient is the handheld smartphone containing closed loop algorithm and communicating wirelessly with the insulin pump.

8.4.4 Required training

Prior to commencement of the study, the research team nurses/clinicians at each of the investigation centres will be trained to use the closed loop system and its components. Prior to the use of study devices, participants will be trained to use the study CGM device, the study pump and where appropriate the closed loop system. Competency assessments of the participants' capability to use study devices and the closed loop system will be made. Additional training visits on new study devices for the follow up closed-loop platform will be provided by the study team as required by the participant and competency assessments on the study devices will be completed.

8.4.5 Precautions

During treatment with insulin there is a risk of hypoglycaemia and hyperglycaemia. In-hospital testing and Hazard Analysis both documented reduced risk of hypoglycaemia and hyperglycaemia during

Boughton C, *et al. BMJ Open* 2020; 10:e033500. doi: 10.1136/bmjopen-2019-033500

day and night closed loop compared to conventional treatment. Addition of pump suspend feature will further increase safety.

8.4.6 Accountability of the method under investigation

The local Investigator will provide training for the study participants and will make every effort, through regular contact, to ascertain that the closed loop system is used for the study purposes only. Devices will be identified using batch/lot/serial numbers and the location of investigational devices and their dates of use by subjects will be documented throughout the study.

9 Study schedule

9.1 Overview

The study will be co-ordinated from the Institute of Metabolic Science, Addenbrooke's Hospital, Cambridge, and performed at the following sites:

1. Addenbrooke's Hospital, Cambridge, UK
2. Leeds Teaching Hospital, Leeds, UK
3. Alder Hey Children's Hospital, Liverpool, UK
4. Nottingham Hospital, Nottingham, UK
5. Oxford Children's Hospital, Oxford, UK
6. Southampton Children's Hospital, Southampton, UK
7. Royal Hospital for Children and Young People, Edinburgh, UK

After recruitment, consent, and run-in period, subjects will be randomised for 24 months home use of real-time CGM combined with automated day and night closed loop insulin delivery or 24 months during which they will apply standard basal bolus therapy.

The study includes up to 14 visits and 1 telephone/email contact for subjects completing the study. After run in and randomisation, visits will be conducted every 3 months. The visit to set up automated closed loop for the first time may take place in the home setting or alternatively in an in-patient facility. All participants will continue to be seen by their clinical team at frequencies as appropriate in line with usual clinical practice. All study visits will be scheduled in addition to routine visits and will be performed by the research team only. For convenience, dates for study visits will be arranged along with routine clinic appointments if possible. Maximum time in study is 24 months.

At 24 months, all participants will have the option to continue with their current treatment for a further 24 months in an extension phase. Contacts will be conducted every 3 months with study visits at 36 and 48 months. All participants will continue to be seen by their clinical team at frequencies as appropriate in line with usual clinical practice.

Table 1 outlines study activities when participant is randomised to day and night closed loop (intervention group).

Table 2 outlines study activities when participant is randomised to standard therapy alone (control group).

Table 1: Schedule of study visits / phone contacts when the participant is randomised to closed loop (intervention group)

	Visit/ contact	Description	Start relative to previous / next Visit / Activity	Duration
Run in period	Visit 1	Recruitment and screening visit: Consent/assent; inclusion, exclusion; screening blood sample	Within 21 days of diagnosis	2 hours
	Visit 2	Baseline visit: HbA1c, MMTT, blinded CGM, questionnaires, computerised cognitive testing, bloods for immunological analyses	7 to 21 days after diagnosis	3-4hours
		Randomisation		
Insulin pump & CGM Training	Visit 3	Insulin pump training, initiation study pump	Within 1 week of Visit 2	3-4 hours
	Visit 4	CGM training, initiation of CGM	Within 0 to 7 days of Visit 3 (Visit 4 may coincide with Visit 3; Training visits can be repeated)	2 hours
Closed loop insulin delivery (24 months)	*Visit 5	CL initiation at clinic/home	Within 6 weeks of diagnosis	3-4 hours
	Contact	Review use of study devices, study update	1 week after Visit 5 (±3 days)	<0.5 hour
	*Visit 6	HbA1c, data download, blinded CGM	After 3 months of diagnosis (±1 week)	<1 hour
	Visit 7	MMTT, HbA1c, bloods for immunological analyses, data download, blinded CGM, sleep quality assessment	After 6 months of diagnosis (±2 weeks)	3-4hours
	*Visit 8	HbA1c, data download, blinded CGM	After 9 months of diagnosis (±2 weeks)	<1 hour
	Visit 9	MMTT, HbA1c, bloods for immunological analyses, data download, blinded CGM, questionnaires, computerised cognitive testing, interviews, sleep quality assessment	After 12 months of diagnosis (±2 weeks)	3-4 hours
	*Visit 10	HbA1c, data download, blinded CGM	After 15 months of diagnosis (±2 weeks)	<1 hour
	*Visit 11	HbA1c, data download, blinded CGM	After 18 months of diagnosis (±2 weeks)	<1 hour
	*Visit 12	HbA1c, data download, blinded CGM	After 21 months of diagnosis (±2 weeks)	<1 hour
	*Visit 13	Blinded CGM, sleep quality assessment	Between Visit 12 and Visit 14 (Visit 13 may coincide with visit 14)	<0.5 hour
	Visit 14	End of closed loop treatment: MMTT, HbA1c, data download, bloods for immunological analyses, questionnaires, computerised cognitive testing, focus groups	After 24 months of diagnosis (±2 weeks)	4-5 hours

Boughton C, *et al. BMJ Open* 2020; 10:e033500. doi: 10.1136/bmjopen-2019-033500

Optional extension phase (24 months)	Contact	Review use of study devices, HbA1c, study update	3 months after Visit 14 (±2 weeks)	<0.5 hour
	Contact	Review use of study devices, HbA1c, study update	6 months after Visit 14 (±2 weeks)	<0.5 hour
	Contact	Review use of study devices, HbA1c, study update	9 months after Visit 14 (±2 weeks)	<0.5 hour
	Visit 15	Fasted C-peptide and glucose, HbA1c, blinded CGM, questionnaires	After 36 months of diagnosis (±2 weeks)	<1 hour
	Contact	Review use of study devices, HbA1c, study update	3 months after Visit 15 (±2 weeks)	<0.5 hour
	Contact	Review use of study devices, HbA1c, study update	6 months after Visit 15 (±2 weeks)	<0.5 hour
	Contact	Review use of study devices, HbA1c, study update	9 months after Visit 15 (±2 weeks)	<0.5 hour
	*Visit 16	Blinded CGM	2 weeks before Visit 17 (±2 weeks)	<0.5 hour
	Visit 17	Fasted C-peptide and glucose, HbA1c, blinded CGM review, questionnaires	After 48 months of diagnosis (±2 weeks)	<1 hour
* could be done at home				

Table 2: Schedule of study visits / phone contacts when the participant is randomised to standard therapy i.e. multiply daily injections (control group)

	Visit/ contact	Description	Start relative to previous / next Visit / Activity	Duration
Run in period	Visit 1	Recruitment and screening visit: Consent/assent; inclusion, exclusion; screening blood sample	Within 21 days of diagnosis	2-hours
	Visit 2	Baseline visit: HbA1c, MMTT, blinded CGM, questionnaires, computerised cognitive testing, bloods for immunological analyses	7 to 21 days after diagnosis	3-4hours
		Randomisation		
Additional Training	Visit 3	Training on carbohydrate counting	Within 1 week of Visit 2	2 hours
	Visit 4	Training on insulin dose adjustment	Within 0 to 7 days of Visit 3 (Visit 4 may coincide with Visit 3; Training visits can be repeated)	2 hours
Multiple daily injection of insulin (24 months)	*Visit 5	MDI arm start visit	Within 6 weeks of diagnosis	<1 hour
	Contact	Study update	1 week after Visit 5 (±3 days)	<0.5 hour
	**Visit 6	HbA1c, blinded CGM	After 3 months of diagnosis (±1 week)	<1 hour
	Visit 7	MMTT, HbA1c, bloods for immunological analyses, blinded CGM, sleep quality assessment	After 6 months of diagnosis (±2 weeks)	3-4 hours

	**Visit 8	HbA1c, blinded CGM	After 9 months of diagnosis (±2 weeks)	<1 hour
	Visit 9	MMTT, HbA1c, bloods for immunological analyses, blinded CGM, questionnaires, computerised cognitive testing, sleep quality assessment	After 12 months of diagnosis (±2 weeks)	3-4 hours
	**Visit 10	HbA1c, blinded CGM	After 15 months of diagnosis (±2 weeks)	<1 hour
	**Visit 11	HbA1c, blinded CGM	After 18 months of diagnosis (±2 weeks)	<1 hour
	**Visit 12	HbA1c, blinded CGM	After 21 months of diagnosis (±2 weeks)	<1 hour
	**Visit 13	Blinded CGM, sleep quality assessment	Between Visit 12 and Visit 14, (may coincide with visit 14)	<1 hour
	Visit 14	End of closed loop treatment: MMTT, HbA1c, bloods for immunological analyses, questionnaires, computerised cognitive testing, focus groups	After 24 months of diagnosis (±2 weeks)	4-5 hours
**Optional extension phase (24 months)**	Contact	Study update, HbA1c	3 months after Visit 14 (±2 weeks)	<0.5 hour
	Contact	Study update, HbA1c	6 months after Visit 14 (±2 weeks)	<0.5 hour
	Contact	Study update, HbA1c	9 months after Visit 14 (±2 weeks)	<0.5 hour
	Visit 15	Fasted C-peptide and glucose, HbA1c, blinded CGM, questionnaires	After 36 months of diagnosis (±2 weeks)	<1 hour
	Contact	Study update, HbA1c	3 months after Visit 15 (±2 weeks)	<0.5 hour
	Contact	Study update, HbA1c	6 months after Visit 15 (±2 weeks)	<0.5 hour
	Contact	Study update, HbA1c	9 months after Visit 15 (±2 weeks)	<0.5 hour
	*Visit 16	Blinded CGM	2 weeks before Visit 17 (±2 weeks)	<0.5 hour
	Visit 17	Fasted C-peptide and glucose, HbA1c, blinded CGM review, questionnaires.	After 48 months of diagnosis (±2 weeks)	<1 hour
* could be done at home or phone/email, ** could be done at home				

9.2 Recruitment visit and screening assessment (Visit 1)

Once the subjects have agreed to participate in the study, they will be invited for the recruitment visit, when the following activities will be performed by the research team:

- written informed consent/assent
- checking inclusion and exclusion criteria
- medical (diabetes) history
- body weight and height measurement; calculation of BMI
- blood pressure measurement

- record of current insulin therapy
- urine pregnancy test (females of child-bearing potential)

9.2.1 Screening and reference blood sampling

If not done at diagnosis, a blood sample will be taken for assessment of full blood count, thyroid function (TSH, fT4), anti-transglutaminase antibodies and IgA (all measured at a local laboratory). Less than 15 ml of whole blood will be taken from each participant if need be.

9.3 Baseline visit (Visit 2)

Subject will arrive at the clinical facility in the morning at the agreed time. This session will include the following activities:

- Body weight measurement (see 12.1.1)
- Cognitive function will be assessed using validated computerized cognitive tests (see 12.2.2)
- A mixed meal tolerance test (MMTT) to assess residual beta cell function (see 12.1.2).
- In addition to measurements of C-peptide and blood glucose (MMTT), blood samples for analysis of HbA1c, lipid profile and immunological parameters will be taken (see 12.4)
- Validated questionnaires will be distributed to assess quality of life and diabetes management (see 12.2.1).
- At the end of this session, participants will be fitted with a blinded continuous glucose monitoring (CGM) device to assess baseline glycaemic control. Instructions on how to safely remove and send back the device will be provided (see 12.1.3.1)

9.4 Run in period

All participants will receive core diabetes training as per usual clinical practice during an up to three week run-in period following diagnosis, study consent/assent and baseline assessment. These non-study-specific training sessions should include introduction to blood glucose monitoring, blood glucose targets, insulin therapy (including handling of insulin pens and injections), physical activity advice, hypoglycaemia management, dealing with hyperglycaemia (incl. blood ketone monitoring), sick day rules, and carbohydrate counting (for details see section 8.2).

All subjects will be provided with 24 hour telephone helpline and will also be given written instructions about when to contact clinical team.

Boughton C, *et al. BMJ Open* 2020; 10:e033500. doi: 10.1136/bmjopen-2019-033500

9.5 Randomisation

On completion of Visit 2, eligible subjects will be randomised in a 1:1 ratio using central randomisation software to the use of day and night closed loop or to standard therapy. The randomisation will be stratified by site, age, and possibly gender.

9.6 Post-randomisation training (Visits 3 and 4)

9.6.1 Closed loop intervention

Participants randomised to the closed loop group will receive additional training sessions following randomisation to cover key aspects of insulin pump use and CGM, prior to starting closed loop insulin delivery. Particular attention will be paid to:

- Insulin cartridge and infusion set changes and correct priming procedure
- Carbohydrate counting and the use of the bolus calculator on the pump
- Hypo- and hyperglycaemia management using an insulin pump
- Sensor insertion and calibration
- Uploading pump data

Written easy to use guidelines for the operation of insulin pump and CGM will be provided. This session will be conducted by a professional pump educator and/or member of the study team. Device manual guides will be provided. Competency on the use of study pump and CGM will be assessed.

Guidelines for health care professional with respect to initiation of insulin pump therapy and continuous glucose monitoring will be provided separately in the study manual.

9.6.2 Standard therapy (control intervention)

Following randomisation participants in the control group will receive additional training sessions to complement the core training as provided during the run-in: Particular attention will be paid to:

- Carbohydrate counting
- Understanding insulin to carb ratios and correction factors
- Data review, pattern recognition
- Adjusting insulin doses

Written easy to use guidelines will be provided. These sessions will be conducted by an experienced educator. Guidelines for health care professional with respect to multiple daily injection therapy will be provided separately in the study manual.

9.7 Initiation of treatment arm (Visit 5)

For participants competent in the accomplishment of their respective treatment regimen (MDI or pump and CGM), the start of study arm will be initiated within at least two weeks of visit 3, but within 6 weeks of diagnosis at latest. Initiation visits could be either done at the hospital clinic, the clinical facility, subject's home or other suitable meeting place. Current insulin therapy will be recorded.

Subjects will be provided with 24 hour telephone helpline and will also be given written instructions on how to deal with low and high glucose at home and when to contact study team.

9.7.1 Closed loop intervention

Those subjects randomised to closed loop intervention will receive training required for safe and effective use of the closed loop system and pump suspend feature. The visit will include training on connection and disconnection of the closed loop system, switching between closed loop and usual pump therapy and trouble-shooting aspects. Written step by step guidance will also be provided. During the initiation visit, subjects will use closed loop system under supervision by study staff. Subjects will have a meal according to their own choice and will be required to deliver a meal bolus for the given meal. Competency on the use of closed loop system will be assessed by the study team. Only subjects who demonstrate competency on use of the system will be allowed to continue to the home study phase. Participants are expected to use the closed loop at all times during the 24 months intervention period.

9.7.2 Standard therapy (control intervention)

In subjects randomised to control intervention, diabetes management skills will be revisited, data will be reviewed, and dose adjustments will be made if need be. Additionally, study schedule and study related procedures will be discussed again. In the control intervention arm, visit 5 can be done via telephone/email, too. Participants will apply standard insulin therapy using multiple daily injections via insulin pens during the 24 months control period. However, participants will be allowed to switch to insulin pump therapy if clinically indicated applying NICE criteria according to usual clinical practice. As per the intention to treat approach of this study, participants switching to insulin pump treatment during control intervention will continue to be followed up unless consent is withdrawn or other withdrawal criteria apply (see section 9.14).

9.8 Telephone/email contact after initiation of treatment arm

Participants and/or parents will be contacted by email or telephone within one week after initiation of the respective study arm. The purpose of this contact would be to troubleshoot any problems, and to record any adverse events, device deficiencies, and changes in insulin settings, other medical conditions and/or medication. Thereafter, participants will be followed up through study visits at 3-monthly intervals. Throughout the trial, subjects/parents and/or the clinical team are free to adjust insulin therapy as per usual clinical practice, but no active treatment optimisation will be undertaken by the study team.

9.9 Routine follow up visits (Visit 6, 8, 10, 11, 12)

The subjects will be invited to attend the first follow up visit 3 months after diagnosis. Thereafter, visits will take place every 3 months for the remainder of the study. Routine follow up visits can take place at the hospital clinic, home or other suitable meeting place, according to participants' convenience. A routine follow up visit should not take longer than approximately 30 minutes.

The purpose of this visit would be to record any adverse events, device deficiencies, and changes in insulin settings, other medical conditions and/or medication. An HbA1c sample will be taken to monitor overall glucose control. For subjects randomised to closed loop intervention, data from study devices will be downloaded.

At the end of this session, participants of both study arms will be fitted with a blinded continuous glucose monitoring (CGM) sensor. The sensor will be worn at home for up to 14 days. If the sensor fails (i.e. does not provide any data) or sensor function is interrupted prematurely (detached sensor), another sensor may be inserted. The sensor(s) will be sent back to the research team or collected by the research team once the sensor life has expired and/or the sensor has detached. The sensor data may be used to optimise insulin delivery. Sensors can be applied 2 weeks prior to the study visit and data reviewed at the study visit if required.

9.10 Follow up visits including MMTT (Visit 7, 9)

The subject will attend the research centre for the purpose of a mixed meal tolerance test (see section 12.1.2) at 6 months and 12 months after diagnosis. Otherwise, procedures are identical to routine follow up visit (including HbA1c sampling and blinded CGM; see section 9.9). Follow up visits including MMTT should not last longer than 3-4 hours.

Boughton C, *et al. BMJ Open* 2020; 10:e033500. doi: 10.1136/bmjopen-2019-033500

- Body weight, height and blood pressure will be measured
- In addition to measurements of C-peptide and blood glucose (MMTT), blood samples for analysis of immunological parameters will be taken (see section 12.4). At 12 months, blood lipid profile will be additionally assessed

At the end of the session, participants will be fitted with the Actiwatch (a simple wristwatch used to measure sleep non-invasively in the participant's home), and the Pittsburgh Sleep Quality Index and a sleep diary will be handed out (see section 12.2.3). The wristwatch will be worn for up to 7 days. Concomitantly, a sleep diary will be kept, and PSQI questionnaires will be completed by the participants (assisted by their parents/carers if need be), and their parents/carers. The wristwatch, the completed diary and questionnaires, along with the blinded CGM device will be will be sent back to the research team or collected by the research team.

At 12 months, a subset of participants/family members in the closed loop arm will be invited to take part in a qualitative interview with the trained personnel (see 12.2.4.1).

At 12 months, cognitive function will be assessed using validated computerized cognitive tests (see section 12.2.2).

9.11 Penultimate visit 13

For assessment of glycaemic control during the final 3-month period of the trial, participants of both study arms will be asked to attend the clinical facility at least 2 weeks before the end of study visits (Visit 14). Alternatively, this visit could take place in the subject's home. Participants will be fitted with a blinded continuous glucose monitoring (CGM) sensor. The sensor will be worn at home for up to 14 days.

Additionally, participants will be fitted with the Actiwatch wristwatch, and the Pittsburgh Sleep Quality Index (PSQI) and a sleep diary will be handed out (details re sleep assessment see section 12.2.3) for assessment of sleep. The wristwatch will be worn for 7 days. Concomitantly, a sleep diary will be kept, and the PSQI will be completed by the participants (assisted by their parents/carers if need be), and their parents/carers.

All devices (blinded CGM, wristwatch) and the completed sleep diary and questionnaires, along with the blinded CGM device will be returned to the research team once the sensor life has expired and/or the sensor has detached, or at the end of study visit (Visit 14) at latest.

For those participants continuing with the extension phase, this visit can be combined with Visit 14.

9.12 End of study visit (Visit 14)

The subject will be invited to attend the research centre approximately 3 months after visit 12. This would be the end of 24 month study period. The subject will have a MMTT and a blood sample for HbA1c, immunological analyses and lipid profile will be taken. Body weight, height and blood pressure measurements will be made. Subject will be asked to complete questionnaires as outlined in section 12.2.1. Cognitive function will be assessed using validated computerized cognitive tests (see section 12.2.2). Additionally, subjects/family members will be invited to attend focus group discussions at their clinical site (see section 12.2.4.2) following the End of study visit. For participants of the closed loop intervention, who are not continuing with the extension phase, study device data will be downloaded and subjects will start transition to usual care.

9.13 Transition to usual care

Following visit 14, for those not wishing to continue in the extension phase, (or following Visit 17 for those completing the extension phase) subjects randomised to closed loop will revert to conventional insulin therapy by switching back to either insulin pump alone or multiple daily injection therapy as per decision of the clinical team. Refresher training sessions on standard therapy will be provided by the routine clinical team to facilitate transition to usual care over a period of up to 8 weeks. During this period, subjects will be able to continue using the study insulin pump and/or study CGM (but not closed-loop) as required to facilitate transition. Subjects of the control intervention will continue with standard therapy as before.

9.14 Optional extension phase

At 24 months, all subjects will be invited to continue in an extension phase of the study for a further 24 months with the treatment allocated at randomisation. Participants opting to continue with the extension phase will be asked to re-consent.

9.14.1 Routine follow up contacts

Participants and/or parents will be contacted every 3 months during the extension phase. This can be at routine clinic appointments or by email/telephone. The purpose of this contact would be to troubleshoot any problems, and to record any adverse events, device deficiencies, and changes in insulin requirements other medical conditions and/or medication. Local HbA1c values will be recorded at this visit. Throughout the extension phase, subjects/parents and/or the clinical team are

free to adjust insulin therapy as per usual clinical practice, but no active treatment optimisation will be undertaken by the study team.

9.14.2 Study visits

At 36 and 48 months after diagnosis, participants of both study arms will be invited to attend the research centre. The subject will have a fasting blood sample for C-peptide, glucose, HbA1c and lipids. Body weight and height measurements will be made. Subjects will be asked to complete questionnaires as outlined in section 12.2.1. Participants of both study arms will be fitted with a blinded continuous glucose monitoring (CGM) sensor. For the final study visit (Visit 17) the blinded CGM will be applied 2 weeks prior (Visit 16).

Participants in the control intervention arm already using a FreeStyle Libre continuous glucose monitoring sensor will be able to use their own sensor, rather than the study blinded sensor. The sensor will be worn at home for up to 14 days. If the sensor fails (i.e. does not provide any data) or sensor function is interrupted prematurely (detached sensor), another sensor may be inserted. The sensor(s) will be sent back to the research team or collected by the research team once the sensor life has expired and/or the sensor has detached. The sensor data may be used to optimise insulin delivery. Sensors can be applied 2 weeks prior to the study visit and data reviewed at the study visit if required.

At the end of the extension phase (Visit 17) at 48 months after diagnosis, subjects in the closed-loop arm will transition to usual care as described in 9.13.

9.15 Participant withdrawal criteria

The following pre-randomisation withdrawal criterion will apply:

1. Subject/Family is unable to demonstrate safe application of multiple daily injection therapy during run-in period as judged by the investigator

The following pre- and post-randomisation withdrawal criteria will apply:

2. Subject is unable to demonstrate safe use of MDI or study insulin pump and/or CGM during post randomisation training period as judged by the investigator
3. Subject fails to demonstrate compliance as described in sections 9.6.1 and 9.6.2 with MDI therapy or study insulin pump and / or CGM during post randomisation training period
4. Subjects may terminate participation in the study at any time without necessarily giving a reason and without any personal disadvantage

5. Significant protocol violation or non-compliance
6. Recurrent severe hypoglycaemia events not related to the use of the closed loop system
7. Recurrent severe hyperglycaemia event/DKA unrelated to infusion site failure and related to the use of the closed loop system
8. Decision by the investigator or the Sponsor that termination is in the subject's best medical interest
9. Allergic reaction to insulin
10. Allergic reaction to adhesive surface of infusion set or glucose sensor
11. If patient cannot be contacted in 12 weeks subject will be considered lost to follow up

If participant wishes to withdraw from trial treatment, sites should nevertheless explain the importance of remaining on trial follow-up or, failing this, of allowing routine follow-up data to be used for trial purpose. Generally, follow-up will continue unless the participant explicitly also withdraws consent for follow-up.

Subjects who are withdrawn for reasons stated in (5) to (11) will be invited to undergo mixed meal tolerance test and to provide blood sample at the end of the planned study intervention for the assessment of HbA1c.

In consenting to the trial, participants are consenting to trial treatment, follow-up and data collection. If voluntary withdrawal occurs, the participant (or parent/legal representative) should be asked to allow continuation of scheduled evaluations, complete an end-of-study evaluation, and be given appropriate care under medical supervision until symptoms of any adverse event resolved or the participant’s condition becomes stable. Follow-up of these participants will be continued through the research nurse and the lead investigator at each site and, where these are unsuccessful, through child’s local paediatric diabetes service or via their GP.

The research team plans to enable ongoing observation of participants as they move from paediatric to adult health care services. This ongoing observation will be the focus of a separate application for funding.

9.16 Participant transfer

For the participant moving from the area, every effort should be made for the participant to be followed-up at another participating trial site and for this trial site to take over responsibility for the participant or follow-up at the local paediatric diabetes services or via their GP.

The participant (or parent/legal representative) will have to sign a new consent form at the new site, and until this occurs, the participant remains the responsibility of the original site. The Chief Investigator, or the Sponsor or its representative should be notified in writing of participant transfer.

9.17 Study stopping criteria

The study may be stopped if three consecutive participants withdraw on safety grounds or on the advice of an independent Data Monitoring and Ethics Committee (DMEC).

9.18 Co-enrolment guidelines

To avoid potentially confounding issues, ideally participants should not be recruited into other trials. Where recruitment into another study is considered to be appropriate and without having any detrimental effect on the present study, this must first be discussed with the Chief Investigator, or the Sponsor or its representative.

9.19 Support telephone line

There will be a 24-hour telephone helpline to the local clinical and research teams for subjects in case of any technical device or problems related to diabetes management such as hypo- or hyperglycaemia. The local research team will have access to central 24 hour advice on technical issues.

9.20 Study peer support system

A peer support system will be available in this trial in order to enhance trial recruitment, acceptability and retention. Potential participants can freely choose whether to have a peer supporter and whether to share any personal health information.

Peer supporters or buddies will be inside members of the target population with personal experience of both recent diagnosis of type 1 diabetes and participation in this trial, e.g. other trial participants and their families at a later stage of the trial, or former participants/participating families. Peer supporters are neither qualified nor licensed to diagnose, give medical advice, or recommend changes to medications. No payments will be made to peer supporters.

Potential peer supporters will be approached and selected by the study team at a later stage of the trial. Separate consent/assent forms for buddies will be signed, as well as an oath of confidentiality. Guidance and training for buddies will be provided including the following topics: the buddy's role and possible role conflict, what kinds of information the buddy can best provide and when to refer to a health professional, types and amount of contact to expect, potential problems and trouble-shooting, confidentiality.

Contact details of a selected buddy will be forwarded to the potential participant, once the buddy’s availability has been confirmed. The abilities and qualities of the peer supporters will be matched to

the needs of those to be supported. The first contact by phone will be initiated by the potential participant according to availability of the buddy. Further contacts will be arranged independently from the study team if wished. Buddies and participants may terminate participation in the buddy system at any time.

9.21 Subject reimbursement

The study will provide the insulin pump/CGM device, CGM sensors, and closed loop components. Other related consumables (e.g. infusion sets and glucose test strips) will be at least partially covered. As an appreciation for the participants' involvement in the study, vouchers/payment will be provided at each of the visits including a MMTT as specified in the participant information sheet and REC application form. A capped contribution to travel expenses may be offered. After completing the study, subjects will not keep the study devices. They will revert to conventional insulin therapy or insulin pump therapy based on the decision of the local clinical team.

During the extension phase, as an appreciation for the participants' involvement in the study, vouchers/payment will be provided at each of the visits including questionnaires as specified in the participant information sheet and REC application form.

9.22 Retention strategies

As an appreciation for the participants' involvement in the study, certificates may be provided after completion of 24 months in the study and after completing the extension phase. A template of the certificate will be reviewed by the REC. Newsletters with updates about the study will be distributed to study participants and their families up to every 6 months. Online events (e.g. webinars) may be undertaken during the study. Information regarding these events will be circulated to participants. Participation in these events will be entirely voluntary.

10 Endpoints

10.1 Primary endpoint

The primary endpoint is the area under the stimulated C-peptide curve of a mixed meal glucose tolerance test conducted 12 months post diagnosis.

10.2 Secondary endpoints

Secondary endpoints include:

- Mean stimulated C-peptide AUC over time (at baseline, 6 and 24 months)
- Overall glucose control and glucose variability

- HbA1c levels (3-monthly assessed)
- Percentage of patients in each group with HbA1c <7.5% (58 mmol/mol)
- Percentage of time spent in with sensor glucose readings in the target range (3.9 to 10mmol/l)*
- Average, standard deviation, and coefficient of variation of sensor glucose levels*

- Hypoglycaemia
  - Percentage of time spent below target glucose (3.9 mmol/l)*
  - Percentage of time with sensor glucose levels <3.5 mmol/l , 3.0 mmol/l and <2.8 mmol/l*
  - AUC of sensor glucose below 3.9 mmol/l and 3.5 mmol/l*
- Hyperglycaemia
  - Time spent with sensor glucose above target (10.0 mmol/l)*
  - Time with sensor glucose levels in significant hyperglycaemia (glucose levels > 16.7 mmol/l)*
- Insulin requirements
  - Total, basal and bolus insulin dose (U/kg)
- Weight
  - Change in body mass index (BMI) standard deviation score
- Blood pressure
- Lipid profile

**based on 3-monthly data from blinded continuous glucose monitoring (CGM)*

Exploratory endpoints will include trends in glucose control and insulin delivery, daytime vs. overnight glucose control, relationships between CL compliance and glucose outcomes, correlation between fasting C-peptide, stimulated C-peptide and C-peptide at 90 minutes as assessed during MMTT, relationship between beta-cell function and immune markers.

For the assessment of long term outcomes (see section 6.2) routine clinical data will be submitted to the research team for a further nine years after the subject has completed the 24 month trial. This will include:

- HbA1c
- Insulin treatment regimen
- BMI

- Insulin dose (units/kg/day)
- Number of severe hypoglycaemic episodes and DKAs
- Complications of type 1 diabetes

10.3 Safety evaluation

Safety evaluation will comprise the number of episodes of severe hypoglycaemia as well as the number of subjects experiencing severe hypoglycaemia, frequency of diabetic ketoacidosis, and number, nature and severity of any other adverse events.

10.4 Utility evaluation

Utility evaluation is the frequency and duration of use of the closed loop system.

10.5 Human Factors Evaluation

Cognitive, emotional, and behavioural characteristics of participating subjects and family members and their response to the closed loop system and clinical trial will be assessed at distinct time-points throughout the trial using validated surveys (perceptions of quality of life, diabetes management, sleep and fear of hypoglycaemia) cognitive tests, as well as qualitative interviews and focus groups. Additionally, sleep will be objectively assessed using wristwatch devices.

10.6 Health economic assessment

Health economic analysis will be performed contrasting the artificial pancreas (closed loop) and conventional insulin therapy (MDI) using a health economic simulation model: the IMS CORE DIABETES MODEL (CDM). Long-term outcomes derived from the simulation will include total direct costs, life expectancy, quality-adjusted life expectancy and time to onset of complications. Incremental costs versus incremental effectiveness (quality-adjusted life years [QALYs]) for closed loop vs multiple daily injection therapy will be compared.

10.7 Extension phase outcomes

Endpoints include:

- Fasting C-peptide and glucose (at 36 and 48 months)
- Overall glucose control and glucose variability
  - HbA1c levels (12-monthly assessed)
  - Percentage of patients in each group with HbA1c <7.5% (58 mmol/mol)

Boughton C, *et al. BMJ Open* 2020; 10:e033500. doi: 10.1136/bmjopen-2019-033500

  - Percentage of time spent in with sensor glucose readings in the target range (3.9 to 10mmol/l)*
  - Average, standard deviation, and coefficient of variation of sensor glucose levels*
- Hypoglycaemia
  - Percentage of time spent below target glucose (3.9 mmol/l)*
  - Percentage of time with sensor glucose levels <3.5 mmol/l , 3.0 mmol/l and <2.8 mmol/l*
  - AUC of sensor glucose below 3.9 mmol/l and 3.5 mmol/l*
- Hyperglycaemia
  - Time spent with sensor glucose above target (10.0 mmol/l)*
  - Time with sensor glucose levels in significant hyperglycaemia (glucose levels > 16.7 mmol/l)*
- Insulin requirements
  - Total, basal and bolus insulin dose (U/kg)
- Lipid profile
- Weight
  - Change in body mass index (BMI) standard deviation score

*based on 12-monthly data from blinded continuous glucose monitoring (CGM)

Safety, utility and human factor evaluations will be assessed as described above.

11 Assessment and reporting of adverse events

11.1 Definitions

11.1.1 Reportable Adverse Events

A reportable Adverse Event is any untoward medical occurrence that meets criteria for a serious adverse event or any unanticipated medical occurrence in a study subject that is study or device-related. Device deficiencies that could have led to a serious adverse device effect will also be reported.

11.1.2 Adverse Events

An adverse event (AE) is any untoward medical occurrence, unintended disease or injury, or untoward clinical signs (including abnormal laboratory findings) in a subject who has received an investigational device, whether or not related to the investigational medical device. This definition

includes events related to the device under investigation or the comparator or to the study procedures. For users or other persons, this definition is restricted to events related to the investigational device. The following anticipated adverse events will not be recorded:

- Non clinically significant skin reactions as judged by investigator
- Pre-existing medical conditions
- New illnesses or conditions not requiring concomitant medication or medical intervention/procedures
- Non severe hypoglycaemia
- Hyperglycaemia without significant ketonaemia

11.1.3 Adverse Device Effect

An Adverse Device Effect (ADE) is an adverse event related to the use of an investigational medical device. This includes adverse events resulting from insufficient or inadequate instructions for use, deployment, implantation, installation, or operation, or any malfunction of the investigational medical device. This definition also includes any event resulting from use error or from intentional misuse of the device under investigation.

11.1.4 Serious Adverse Event

A serious adverse event (SAE) is an adverse event that:

- led to a death
- led to a serious deterioration in the health of the subject, that either resulted in:
  - a life threatening illness or injury
  - a permanent impairment of a body structure or function
  - in-patient hospitalisation or prolonged hospitalisation
  - medical or surgical intervention to prevent life-threatening illness or injury or permanent impairment to a body structure or a body function
- led to foetal distress, foetal death or a congenital abnormality or birth defect

A planned hospitalisation for pre-existing condition, or a procedure required by the study protocol, without a serious deterioration in health, is not considered to be a serious adverse event.

More than one of the above criteria can be applicable to one event. Life-threatening in the definition of a serious adverse event or serious adverse reaction refers to an event in which the subject was at risk of death at the time of the event; it does not refer to an event which hypothetically might have

caused death if it were more severe. Medical judgement should be exercised in deciding whether an adverse event or reaction is serious in other situations.

Important adverse events or reactions that are not immediately life-threatening or do not result in death or hospitalisation but may jeopardise the subject or may require intervention to prevent one of the other outcomes listed in the definition above, should also be considered serious.

The following serious adverse events are anticipated:

- Severe hypoglycaemia
- DKA

11.1.5 Serious Adverse Device Effect

A Serious Adverse Device Effect (SADE) is an adverse device effect that has resulted in any of the consequences characteristic of a serious adverse event.

11.1.6 Unanticipated Serious Adverse Device Effect

An Unanticipated Serious Adverse Device Effect (USADE) is a serious adverse device effect which by its nature, incidence, severity or outcome has not been identified in the current version of the protocol.

An Anticipated Serious Adverse Device Effect (ASADE) is a serious adverse device effect which by its nature, incidence, severity or outcome has been identified in the protocol.

11.1.7 Device Deficiencies

A device deficiency is an inadequacy of a medical device with respect to its identity, quality, durability, reliability, safety or performance. A device deficiency may lead to an Adverse Device Effect or Serious Adverse Device Effect. The following anticipated device deficiencies and device-related issues will not be recorded:

- Infusion set occlusion/leakage not leading to ketonaemia
- Sensor failure due to miscalibration/detachment
- Premature interruption of sensor-life
- Battery lifespan deficiency due to inadequate charging or extensive wireless communication
- CAD error messages not needing system replacement
- Intermittent device communication failure not leading to system replacement

11.1.8 Adverse event intensity

Intensity	Definition
Mild	Patient is aware of signs and symptoms but they are easily tolerated
Moderate	Signs / symptoms cause sufficient discomfort to interfere with usual activities
Severe	Patient is incapable to work or perform usual activities

NB. The term “severe” is often used to describe the intensity (severity) of a specific event. This is not the same as ‘serious’, which is based on patient/event outcome or action criteria (see definition 11.1.4). For example, itching for several days may be rated as severe, but may not be clinically serious.

11.1.9 Adverse event causality

Intensity	Definition
Not assessable	A report suggesting an adverse event, which cannot be judged because information is insufficient or contradictory, and which cannot be supplemented or verified.
Unlikely	A clinical event, including laboratory test abnormality, with a temporal relationship, which makes a causal relationship improbable, and in which other drugs/treatments, chemicals or underlying disease(s) provide plausible explanations.
Possible	A clinical event, including laboratory test abnormality, with a reasonable time sequence to administration of the treatment/use of investigational treatment/device, but which also could be explained by concomitant diseases or other drugs/treatments or chemicals.
Probable	A clinical event, including laboratory test abnormality, with a reasonable time sequence to administration of the treatment/use of medical method/device, unlikely to be attributable to concomitant

	disease(s) or other drugs/treatments or chemicals, and which follows a clinically reasonable response on withdrawal (dechallenge). Rechallenge information is not required to fulfil this definition.
Definite/certain	A clinical event, including laboratory test abnormality, occurring in a plausible time relationship to study treatment/use of medical method/device and which cannot be explained by concomitant disease(s), other drugs/treatments or chemicals. The response to withdrawal of the treatment (dechallenge) should be clinically plausible. The event must be unambiguous, either pharmacologically or as phenomenon, using satisfactory rechallenge procedures if necessary.

(Reference: WHO-UMC Causality Categories)

11.2 Recording and reporting of adverse events, serious adverse events and device deficiencies

11.2.1 Monitoring period of adverse events

The period during which adverse events will be reported is defined as the period from the beginning of the study (obtaining informed consent) until 3 weeks after the end of the study participation. Adverse events that continue after the subject's discontinuation or completion of the study will be followed until their medical outcome is determined or until no further change in the condition is expected. The follow up of AEs may therefore extend after the end of the clinical investigation; however no new AEs will be reported after the trial reporting period.

11.2.2 Recording and reporting of adverse events

Throughout the course of the study, all efforts will be made to remain alert to possible adverse events or untoward findings. The first concern will be the safety of the subject, and appropriate medical intervention will be taken. The investigator will elicit reports of adverse events from the subject at each visit and complete adverse event forms. All AEs, including those the subject reports spontaneously, those the investigators observe, and those the subject reports in response to questions will be recorded on paper or electronic AE forms at each site within seven days of discovering the event.

The study investigator will assess the relationship of any adverse event to be device-related or unrelated by determining if there is a reasonable possibility that the adverse event may have been caused by the study device or study procedures. The individual investigator at each site will be

responsible for managing all adverse events according to local protocols, and decide if reporting is required.

11.2.3 Severe hypoglycaemia

In line with ISPAD guidelines (36), hypoglycaemic events will be considered severe if the event requires assistance of another person due to altered consciousness to actively administer carbohydrate, glucagon, or other resuscitative actions. This means that the participant is impaired cognitively to the point that he/she is unable to treat his or herself, is unable to verbalize his or her needs, is incoherent, disoriented, and/or combative, or experiences seizure or coma. For children who are developmentally too young to independently recognize and react to hypoglycaemia, hypoglycaemia is only considered severe if there are associated signs or symptoms of neuroglycopenia including temporary impairment of cognition; incoherent, disoriented and/or combative behaviour; seizure or coma. If plasma glucose measurements are not available during such an event, neurological recovery attributable to the restoration of plasma glucose to normal is considered sufficient evidence that the event was induced by a low plasma glucose concentration.

Severe hypoglycaemia will be regarded as a foreseeable adverse event and an adverse event form will be completed. Severe hypoglycaemia is not necessarily a serious adverse event and hence may not require immediate reporting to the Sponsor. Non-severe hypoglycaemia will not be reported or considered an adverse event.

11.2.4 Hyperglycaemia, ketonaemia and diabetic ketoacidosis

As per usual clinical practice, subjects will be asked to test and record blood or urine ketones if their finger prick glucose is above 14.0 mmol/l or if feeling sick.

Hyperglycaemic events are recorded as Adverse Events if the event involved DKA, as defined by ISPAD (37) and described below, or in the absence of DKA if evaluation or treatment was obtained at a health care facility for an acute event involving hyperglycaemia or ketosis.

- Hyperglycemia (blood glucose >11 mmol/L [≈200 mg/dL])
- Venous pH <7.3 or bicarbonate <15 mmol/L
- Ketonemia and ketonuria

Boughton C, *et al. BMJ Open* 2020; 10:e033500. doi: 10.1136/bmjopen-2019-033500

11.2.5 Reporting of serious adverse events and serious adverse device effects

When reporting adverse events, all pertinent data protection legislation must be adhered to.

The serious adverse event report should contain the following information*:

1. Study identifier (EudraCT number if applicable)
2. Participant's unique study number
3. Date of birth
4. Event description
5. Start date of event
6. Laboratory tests used and medical interventions used to treat the SAE
7. Planned actions relating to the event, including whether the study device was discontinued
8. Statement on the patient's current state of health
9. Reason for seriousness (i.e. death, life threatening, hospitalisation, disability/incapacity or other)
10. Evaluation of causality (including grade of relatedness) with the following (more than one may apply):
    a. the investigational treatment/medical device
    b. the clinical study/a study specific procedure
    c. other: e. g. concomitant treatment, underlying disease
11. Reporter's name, date and signature

*In the case of incomplete information at the time of initial reporting, all appropriate information should be provided as soon as this becomes available.

The relationship of the SAE to the investigational treatment / medical device should be assessed by the investigator at site, as should the anticipated or unanticipated nature of any SAEs and SADEs.

All SAEs whether or not deemed investigational method/device related and whether anticipated or unanticipated must be reported to the Sponsor by email or fax within 24 hours (one working day) of the Investigator learning of its occurrence.

SAEs should be reported to:

Stephen Kelleher
Cambridge University Hospitals
NHS Foundation Trust
Box 277, Addenbrooke's Hospital
Hills Road, Cambridge, CB2 0QQ, UK
Phone: +44 (0) 1223 217418
Fax: +44 (0) 1223 348494
E-mail: enquiries@addenbrookes.nhs.uk

A written report must follow within five working days and is to include a full description of the event and sequelae, in the format detailed on the Serious Adverse Event reporting form. If applicable, the Sponsor will notify the competent authority of all Serious Adverse Events in line with pertinent legal requirements.

The Investigator will notify the Research Ethics Committee (REC) in UK of all Serious Adverse Events in line with pertinent legal requirements. The Investigator will inform the Sponsor about all reports sent to the reporting organisation including follow-up information and answers by the reporting organisation. The local investigator is responsible for informing other site principal investigators and the CI of all SAEs.

The regulatory authority (MHRA) will be notified of all SAEs as soon as possible within ten days of the event occurring during the study. The main REC will be notified of all unexpected and related SAEs within 15 days of the occurrence of the event.

11.2.6 Recording and reporting of device deficiencies

All device deficiencies will be documented throughout the study. The investigator at each site will be responsible for managing all device deficiencies and determine and document in writing whether they could have led to a serious adverse device effect.

All device deficiencies that might have led to a serious adverse device effect(s) if: suitable action had not been taken; intervention had not been made, or if circumstances had been less fortunate, must be reported to the Sponsor as for SAEs/SADEs.

Boughton C, *et al. BMJ Open* 2020; 10:e033500. doi: 10.1136/bmjopen-2019-033500

11.2.7 Reporting of Pregnancy

Study participants will not routinely be tested for pregnancy as part of the trial follow up process. Any pregnancy which does occur during the course of the study should be reported to the Sponsor as indicated in section 11.2.5 immediately. It is at the investigator’s discretion to decide whether the individual should be instructed to stop study treatment. All pregnancies that occur during trial treatment, or within seven days of finishing treatment, need to be followed up until completion and reported separately.

11.2.8 Healthcare arrangements and compensation for adverse events

Healthcare arrangements for subjects who suffer an adverse event as a result of participating in the study may include advice from clinical members of the study team or the patient’s treating diabetes team, or use of emergency health services.

If an adverse event occurs, there are no special compensation arrangements unless this was due to the negligence of one of the clinical investigators or due to harm resulting from study protocol design. In this case subjects may have grounds for legal action for compensation. The normal national complaints mechanism will be available. In addition, any harm arising due to study design (both negligent and non-negligent) will be covered under Sponsor’s insurance policy as applicable.

11.3 Anticipated adverse events, risks and benefits

11.3.1 Risks and anticipated adverse events

Known risks represent hazardous situations which may result in anticipated adverse events. In the following text, where appropriate, the term “risk” and “anticipated adverse events” are used interchangeably without affecting meaning.

11.3.2 Hypoglycaemia and hyperglycaemia

Subjects with type 1 diabetes have a pre-existing risk for hypoglycaemia and hyperglycaemia. Potential risks are:

- Risk of mild to moderate hypoglycaemia and associated symptoms such as sweating, trembling, difficulty thinking and dizziness. There is also a rare risk of severe hypoglycaemia when conscious level is altered, needing help from a third party to correct the hypoglycaemia. These risks are pre-existent in any patient with type 1 diabetes and the study objective is to develop systems to minimise these risks

Boughton C, *et al. BMJ Open* 2020; 10:e033500. doi: 10.1136/bmjopen-2019-033500

- Risk of possible mild to moderate hyperglycaemia similar to the risk that a subject with type 1 diabetes experiences on a daily basis
- Risk of hyperglycaemia leading to diabetic ketoacidosis (DKA). This risk is pre-existent in any patient with type 1 diabetes.

11.3.3 Blood sampling

Subjects will be required to have nine blood tests (venepuncture) during the whole study. Those participating in the extension phase will have an additional two fasting blood tests. Venepuncture is required annually as part of the annual review for people with diabetes, and in some places venepuncture is required every 3 to 12 months for assessment of HbA1c. Potential risks include:

- Slight discomfort or bruising at the site (common)
- Excess bleeding at the site (unlikely)
- Infection at the site (rare)

Local anaesthetic cream or spray may be used to minimise the discomfort.

11.3.4 Finger-prick blood glucose measurements

Finger-prick tests may produce pain and/or bruising at the site.

11.3.5 Insulin injection therapy

Potential risks associated with multiple daily injection therapy include:

- Slight discomfort at the time of insulin injection (common)
- Slight bruising at the site of injection (common)
- Bleeding at injection site (rare)
- Infection at the site of injection (rare)
- Insulin pen malfunction and mechanical problems (rare)
- Allergy to insulin (very rare)
- Lipodystrophy / lipoatrophy (very rare)

11.3.6 Insulin pump therapy

Potential risks associated with insulin pump therapy include:

- Slight discomfort at the time of insertion of the insulin delivery cannula (common)
- Slight bruising at the site of insertion (common)

Boughton C, *et al. BMJ Open* 2020; 10:e033500. doi: 10.1136/bmjopen-2019-033500

- Bleeding at insertion site (rare)
- Infection at the site of insertion (rare)
- Allergy to the insulin delivery cannula or adhesive (rare)
- Infusion set and cannula occlusions (rare)
- Insulin pump malfunction and mechanical problems (rare)
- Allergy to insulin (very rare)
- Lipodystrophy / lipoatrophy (very rare)

11.3.7 Continuous glucose monitoring

Potential risks associated with CGM:

- Slight discomfort at the time of insertion of CGM (common)
- Slight bruising at the site of insertion (unlikely)
- Bleeding at insertion site (rare)
- Infection at the site of insertion (rare)
- Allergic reaction to the CGM sensor material (rare)

If a skin reaction is classified as severe (the observation is noticeable and bothersome to subject and may indicate infection or risk of infection or potentially life-threatening allergic reaction), an adverse event form will be completed.

11.3.8 Questionnaires, interviews and focus groups

As part of the study, Participants will complete semi-structured interviews, focus groups and questionnaires which include questions about their private attitudes, feelings and behaviour related to diabetes. It is possible that some people may find these questionnaires to be mildly upsetting. Similar questionnaires have been used in previous research and these reactions are uncommon. If questionnaire or conversational responses indicate serious psychological distress as judged by the investigators, appropriate clinical services will be arranged. Any treatment will be documented in the case-report form.

The study team takes the safeguarding of children very seriously and should any concerns be raised during the course of the study, including during the interview and focus group sessions, these concerns will be dealt with in accordance with local policy. Participants and caregivers will be made aware of this.

Boughton C, *et al. BMJ Open* 2020; 10:e033500. doi: 10.1136/bmjopen-2019-033500

11.3.9 Risk Analysis and residual risk associated with the investigational device

A detailed risk analysis for the closed loop system was conducted, according to Cambridge University Hospitals NHS Foundation Trust's standard Risk Assessment Tool. The risk analysis is presented in a separate risk analysis report

The hazard analysis has identified two hazardous situations in which the residual risk assessed exceeds a predefined score after all practicable control measures have been applied. Risk/benefit analyses concerning these hazardous situations have been conducted by experienced and knowledgeable multidisciplinary members of the research team.

The risks/benefit analysis concluded that day and night closed loop is expected to reduce substantially but not to eliminate the risk of plasma glucose levels below 2.0 mmol/l. This is supported by clinical data recorded over more than 100 nights at the clinical research facility, by more than 9 years of total use of closed loop in home settings, by simulations, and is further enhanced by the requirement for a calibration check to be performed every day before breakfast and before evening meal.

11.4 Benefits

It is expected that day and night closed loop system may have an important role in the management of diabetes. Therefore, the results of this study are likely to be beneficial for subjects with diabetes.

It is possible that subjects will not directly benefit from being a part of this study. However, it is also possible that the blood sugar information from the CGM devices along with the information about insulin dosing during day and night closed loop will be useful for subjects' diabetes self-management.

11.5 Data Monitoring and Ethics Committee (DMEC)

An independent Data Monitoring and Ethics Committee (DMEC) will be informed of all serious adverse events and any unanticipated adverse device effects that occur during the study and will review compiled adverse event data at periodic intervals.

Boughton C, *et al. BMJ Open* 2020; 10:e033500. doi: 10.1136/bmjopen-2019-033500

12 Methods and assessments

12.1 Procedures

12.1.1 Height, weight and blood pressure

These will be recorded during the recruitment visit, at the 12 month and 24 month visits. Weight will be also measured at Visit 2 (Baseline Visit) and Visit 7 (6 months) before conducting the MMTT. Height will be measured in centimetres using a calibrated stadiometer. Weight will be measured in kilograms using a calibrated electronic scale. Blood pressure readings will be obtained using automated calibrated BP monitors with the patient seated comfortably for 5 minutes prior to the measurements.

Those participating in the extension phase will have height and weight measured at 36 and 48 months.

12.1.2 Mixed Meal Tolerance Test (MMTT)

MMTT will commence following an overnight fast (from midnight; water permitted). Long-acting insulin or basal rates (for closed loop participants) will continue as normal. Rapid-acting insulin or bolus can be given up to 2 hours before the MMTT to correct for hyperglycaemia (subjects will use their own correction factors). The MMTT will only be performed if participant's blood glucose level is between 4 and 11.1 mmol/l or otherwise will be rescheduled.

Participants will be given a liquid meal according to bodyweight - Sustacal/Boost (Nestle, Switzerland, 17g carbohydrates, 4 g proteins, 3g fat per 100ml) or similar. Venous blood samples for the measurement of C-peptide and plasma glucose will be collected 10 minutes prior to the meal (-10 min), at the time of ingestion (0 minutes), and at 15, 30, 60, 90 and 120 minutes.

If the glucose level at t=120 minutes is >8 mmol/l, a subcutaneous insulin correction dose may be given, either via injection or pump, according to the subject's own insulin sensitivity factor. If the glucose level at t=120 is >14 mmol/l, ketones will be tested by finger prick. If ketones are >0.6 mmol/l, glucose and ketones will be repeated until ketones have decreased <0.6 mmol/l. Following the MMTT the participant will be offered a meal, before leaving the study facility.

12.1.3 Continuous subcutaneous glucose monitoring

Two different continuous glucose monitoring systems (CGM) will be used throughout this trial: a blinded CGM with retrospective sensor glucose data read out, and a real-time system providing a contemporaneous display of sensor readings.

12.1.3.1 Blinded Continuous Subcutaneous Glucose Monitoring

Blinded CGM will be intermittently applied at various periods throughout the trial. During run-in, blinded CGM will serve to gain knowledge of the specific subject's glucose control characteristics before the beginning of any intervention arm. Post-randomisation, blinded CGM will be periodically used following 3-monthly study visits. Participants of both intervention arms will be fitted with a blinded sensor during the study visits (or 2 weeks prior to the study visit) and will wear the sensors until expiry or detachment. Sensors will be returned to the research team thereafter. Secondary glucose endpoints as outlined in 10.2 will be based on glucose data derived from data captured during these up to 14 day periods. Moreover, this data could be used to facilitate insulin dose optimisation during control intervention.

Subjects in the control intervention arm already using a FreeStyle Libre continuous glucose monitoring system will be able to use their own sensor, rather than the study blinded sensor.

Subjects participating in the extension phase will wear a blinded CGM following 12-monthly study visits.

12.1.3.2 Real-time Continuous Subcutaneous Glucose Monitoring

Real-time CGM will be applied during closed loop intervention only. The control algorithm will use the real-time CGM's continuous stream of glucose data to control insulin titration. Data from the real-time CGM system will be downloaded periodically by the participant and during study visits.

12.1.4 Insulin pump data

Data from the study insulin pump will be downloaded periodically by the participant and during study visits.

12.2 Human factors assessment

We are broadly referring to human factors as the cognitive, emotional, and behavioural characteristics of the participants in the study. The human factors assessment battery is grounded in two principles: 1) it is critical to use evidence-based methods that are reliable, valid, and have parallel forms for youth and their caregivers, and 2) both quantitative (i.e., surveys) and qualitative (i.e., interviews, focus groups) data need to be gathered to provide the richest, most comprehensive characterization of the sample and their response to the closed loop system and clinical trial.

12.2.1 Questionnaires

Surveys and tests used in this trial are listed in Table 3. The Measure column lists the construct of interest in bold. Participants/guardians will complete the questionnaires at time-points as indicated in the table. Additionally, feedback questionnaires on closed loop specific experience will be distributed to participants/guardians who have been randomised to the closed loop intervention arm. All results will be evaluated at the end of the study.

Table 3. Human Factors Assessment.

Measure	***Respondent***	***Construct Measured / Relevant Points***	***Duration***	***Time-point***
Paediatric **Quality of Life** Inventory (PedsQL) Diabetes (38)	All youth and all parents	All youth ages 10-18 will complete age-appropriate PedsQL Diabetes module. There are 28 items total and can take up to 5 minutes to complete. Parents will also complete a proxy version.	5-10 min	Baseline, 12 months, 24 months<br><br>(Extension phase: 36 and 48 months)
**Strengths and Difficulties** Questionnaire (SDQ) (39)	All youth and all parents	This is a widely used 25 item self-report inventory behavioural screening questionnaire for children and adolescents. The same 25 items are included in questionnaires for completion by the parents.	5-10 min	Baseline, 12 months, 24 months<br>(Extension phase: 36 and 48 months)
**Hypoglycaemia Fear** Survey (HFS) (40-42)	All youth and all parents	Validated questionnaires (HFS child version, HFS parent version) to measure several dimensions of fear of hypoglycaemia. They consist of a 10-item "Behaviour subscale" that measures behaviours involved in avoidance and over-treatment of hypoglycaemia and a 13-item "Worry subscale" that measures anxiety and fear surrounding hypoglycaemia.	5- 10 min	12 months, 24 months<br>(Extension phase: 36 and 48 months)

Boughton C, *et al. BMJ Open* 2020; 10:e033500. doi: 10.1136/bmjopen-2019-033500

**Cognitive testing** (CogState) (43)	All youth	Selected subtests from this computerised-cognitive testing battery will be completed electronically. See 12.2.2	15-20 min	Baseline,<br>12 months,<br>24 months
Pittsburgh **Sleep Quality** Index (PSQI) (44)	All youth and all parents	The PSQI is a validated 19 item questionnaire that holistically assesses sleep quality and sleep duration.	5-10 min	6 months,<br>12 months,<br>24 months<br>(Extension phase: 36 and 48 months)
**INSPIRE**	Youth and parents in closed loop arm	Measures the psychological side of automated insulin delivery. Child (6-12) and Adolescent versions (13-18) have 18 items; Parent version has 21 items.	5 – 10 min	12 months,<br>24 months<br>(Extension phase: 36 and 48 months)
**PAID-Teen**	All youth	Measures 26 items related to the daily hassles of managing type 1 diabetes, and the degree of diabetes distress that arises from diabetes management.	5 – 10 min	12 months<br>24 months<br>(Extension phase: 36 and 48 months)

12.2.2 Computerized cognitive testing

Participants will complete selected subtests from a computerized test battery, Cogstate (43), at baseline, 12 and 24 months. Cogstate has demonstrated sensitivity to subtle changes in cognition and is designed to accommodate repeated assessment of a single individual. The subtests selected, Identification Task, Two Back Task, Set-Shifting Task and the Continuous Paired Associate Learning Task measure choice reaction time, working memory, mental flexibility and fluid reasoning, and spatial new learning respectively. These skills are known to be sensitive to dysglycaemia (2; 4). Each task takes between 2 and 5 minutes to complete (total testing time 15-20 minutes). Raw data will be transferred electronically in an anonymised format (i.e using a numerical study identifier) to Cogstate for scoring and collation prior to electronic transfer back to the study investigators for statistical analyses.

12.2.3 Measures of sleep quality

Quality, duration and fragmentation of sleep will be assessed subjectively (using the Pittsburgh Sleep Quality Index (PSQI) or similar validated questionnaire, and a daily sleep diary) and objectively (by actigraphy) in participants, as well as subjectively in parents/carers (using the Pittsburgh Sleep Quality Index (PSQI). These measures will be conducted over 7 days at 6, 12 and 24 months post diagnosis during both intervention arms.

The PSQI is a validated 19 item questionnaire that holistically assesses sleep quality and sleep duration. The sleep diary will record time of going to bed and waking, plus time of, and reason for (e.g. urination or infant feeding) any nocturnal awakenings.

An Actiwatch (Philips Respironics, Bend, Oregon, USA) worn on the non-dominant wrist will provide objective measures of sleep and wakefulness based on motor activity - a low cost, non-invasive and objective method for evaluating sleep in free-living participants. Actiwatches will record time in bed and actual sleep time, as well as changes in sleep quality from measures of sleep maintenance, sleep efficiency, sleep latency, fragmentation index, total nocturnal activity, and percentage moving time. Light exposure will be measured by the Actiwatch's photovoltaic sensor.

12.2.4 Qualitative assessment

12.2.4.1 Interviews

An integrated qualitative sub-study aims to explore parents' and youth's views about using closed loop systems; understand the impact of using closed loop systems on diabetes management practices and everyday family life; identify parents' and youth's information and support needs when using closed loop systems.

In-depth interviews will be undertaken at or after 12 months with a subset of at least 15 – 20 youth and at least 15 – 20 parents in the closed loop arm. Purposive sampling will be used to ensure diversity in terms of (a) youth's age and gender (b) parents' occupation/education and (c) family forms. Where possible, parents and youth from the same family will be interviewed. Participants will be interviewed separately unless a joint interview is requested.

12.2.4.2 Focus groups

Focus groups will be conducted at the end of the study (24 months). We will conduct focus groups at each of the study sites. Recruitment will proceed on a heterogeneous composition basis in terms of both randomisation and adult/children participants, in order to maximise the possibility of exploring relevant topics from different perspectives. Four families will be recruited for each focus group, with each participating family consisting of a child and either one or two parents, thus giving a total of between 8 and 12 participants per focus group. Two families in each group will include children randomised to pen MDI, with the remaining two families including children randomised to the closed loop system. Eligible families will be approached for focus group recruitment as soon as the participating child has participated in the study for 12 months; the focus groups will then take place in each study centre as soon as four families have agreed to participate. Minimal demographic details (gender and age) will be collected from participating individuals. We will work from a script of open-ended questions used to gather feedback and reactions to study recruitment and randomisation, study support systems, and the closed loop system. There will also be time for discussion of content raised by participants. Use of a moderator with advanced training will ensure consistency across groups. The moderator will keep time and manage group logistics. Sessions will be digitally audio-taped and transcribed by a trusted professional transcription service.

12.3 Health economic evaluation

The analysis will be performed using the CORE Diabetes Model (CDM; IMS Health, Basel, Switzerland). The CDM is a validated non-product-specific policy analysis tool for cost-effectiveness analysis in both type 1 and type 2 diabetes; a detailed description of the model architecture (including schematic diagrams) and validation is available in publications by Palmer et al (45; 46) and more recently McEwan et al (47). In summary, the model is based on a series of inter-dependent submodels that simulate both acute and long-term diabetes-related complications (angina, myocardial infarction, congestive heart failure, stroke, peripheral vascular disease, diabetic retinopathy, macula oedema, cataract, hypoglycaemia, ketoacidosis, lactic acidosis, depression, oedema, nephropathy and end-stage renal disease, neuropathy, foot ulcer and amputation, and non-specific mortality). The sub-models have a semi-Markov structure and use time, state, time-in-

state and diabetes type-dependent probabilities derived from published sources to simulate disease progression. Monte Carlo simulation using tracker variables is used to overcome the memory-less properties of the standard Markov model and allows for interconnectivity and interaction between individual sub-models.

12.3.1 Simulation cohort and treatment effects

Baseline characteristics of the simulation cohort will come from the trial. They will include: age, sex ratio, Hb1Ac and other risk factors. Treatment effects will be based on the trial findings at 12 months for both arms: closed loop versus MDI.

12.3.2 Costs and utilities

The base-case analysis will be performed from the perspective of the UK National Health Service. Direct costs will be sourced from published literature and where necessary inflated to the current year costs (48-59).

For treatment costs, only the incremental costs between the two arms will be considered, namely the difference between closed loop therapy versus MDI. Average UK costs for will be sourced from the British National Formulary 63.

Health state utility values will be taken from published literature (60) and references therein.

12.4 Laboratory methods

12.4.1 Screening and reference sample

Thyroid function (TSH, fT4) and anti-transglutaminase antibodies with IgA levels (to exclude diagnosis of coeliac disease) and full blood count will be measured locally if not already done during diagnosis.

12.4.2 Lipid profile

Venous blood samples for the measurement of total cholesterol, triglycerides, HDL, and LDL cholesterol will be taken at baseline, 12 months and 24 months post diagnosis, and will be measured locally.

For the extension phase, venous blood samples for the measurement of total cholesterol, triglycerides, HDL, and LDL cholesterol will be taken at 36 and 48 months post-diagnosis and will be measured locally.

Boughton C, *et al. BMJ Open* 2020; 10:e033500. doi: 10.1136/bmjopen-2019-033500

12.4.3 C-peptide

Venous blood samples for the measurement of plasma C-peptide will be taken during Mixed Meal Tolerance Tests (MMTT) at baseline, 6 months, 12 months and 24 months post-diagnosis (see section 12.1.2). Plasma samples for C-peptide will be processed locally and stored deep frozen (-20°C or below) until analysis at a central laboratory.

For the extension phase, venous blood samples for the measurement of fasting plasma C-peptide will be taken at 36 and 48 months post-diagnosis. Plasma samples for C-peptide will be processed locally and stored deep frozen (-20°C or below) until analysis at a central laboratory.

12.4.4 Plasma glucose

Plasma samples for glucose will be taken during MMTT at baseline, 6 months, 12 months and 24 months post-diagnosis (see 9.3.1 and 12.1.2). Plasma samples for glucose will be processed locally and stored until analysis at a central laboratory.

For the extension phase, plasma samples for the measurement of fasting glucose will be taken at 36 and 48 months post-diagnosis. Plasma samples for glucose will be processed locally and stored until analysis at a central laboratory.

12.4.5 HbA1c

Blood samples for the measurement of HbA1c levels will be taken at baseline, at 3 monthly follow up visits, and at the end of the study.

For the extension phase, blood samples for the measurement of HbA1c levels will be taken at 36 and 48 months post-diagnosis.

HbA1c will be measured at a central laboratory using an International Federation of Clinical Chemistry and Laboratory Medicine (IFCC) aligned method. HbA1c testing will follow National Glycohemoglobin Standardization Program (NGSP) standards. A local laboratory/point-of-care backup measurement of HbA1c will be made.

12.4.6 Immunological assessment

If the treatment group achieves “metabolic rest” for the islet cell, it may dampen the immune response. During MMTT at baseline, 6 12 and 24 months, we will take and store blood/serum/plasma samples for a subsequent analysis to assess changes in immune markers in intensively treated participants as a result of decreased metabolic activity of their islet cells or a direct effect of improved glycaemic control. Immunological assessment may include analysis of peripheral blood mononuclear cells (PBMCs) and DNA.

12.5 Blood loss

Test	Source	Tube type	Tube Volume ml	Visit 2 baseline	Visit 6 3 months	Visit 7 6 months	Visit 8 9 months	Visit 9 12 months	Visit 10 15 months	Visit 11 18 months	Visit 12 21 months	Visit 14 24 months	Visit 15 36 months	Visit 17 48 months
C-peptide	Blood	Lith Heparin	1.2	7x (8.4 ml)		7x (8.4 ml)		7x (8.4 ml)				7x (8.4 ml)	1x (1.2 ml)	1x (1.2ml)
Glucose	Blood	Flouride	1.2	7x (8.4 ml)		7x (8.4 ml)		7x (8.4 ml)				7x (8.4 ml)	1x (1.2 ml)	1x (1.2ml)
Lipid profile	Blood	Serum Gel	1.1	1x (1.1 ml)				1x (1.1ml)				1x (1.1 ml)	1x (1.1 ml)	1x (1.1 ml)
HbA1c	Blood	EDTA	1.2	1x (1.2 ml)	1x (1.2 ml)	1x (1.2 ml)	1x (1.2 ml)	1x (1.2 ml)	1x (1.2 ml)	1x (1.2 ml)	1x (1.2 ml)	1x (1.2 ml)	1x (1.2 ml)	1x (1.2 ml)
Immunology	Blood	Lith Heparin	4.9/7.5	1x (30 ml)		1x (20 ml)		1x (20 ml)				1x (20 ml)		
Immunology	Blood	Serum Gel	1.1	1x (1.1ml)		1x (1.1ml)		1x (1.1ml)				1x (1.1ml)		
				50.2 ml	1.2 ml	40.2 ml	1.2 ml	40.2 ml	1.2 ml	1.2 ml	1.2 ml	40.2 ml	4.7 ml	4.7 ml

The total blood loss over two years will be approximately: 175.7 ml.

For the extension phase, the total blood loss over two years will be approximately: 9.4ml.

13 Study materials and products

13.1 Insulin

During run-in and control intervention, rapid acting insulin analogues (insulin aspart, insulin lispro, insulin glulisine or similar or ultra-rapid insulin analogue) and long acting insulin analogues (insulin glargine, insulin detemir or similar) will be delivered subcutaneously using an insulin pen injection device in accordance with manufacturer instructions for use.

During closed loop intervention, rapid acting insulin analogues (insulin aspart, insulin lispro, insulin glulisine or similar or ultra-rapid insulin analogue) will be administered via an insulin pump as described below (see 13.3).

13.2 Multiple daily insulin injections during run-in and control intervention

During run-in and control intervention when multiple daily injection therapy will be applied, insulin will be administered using CE-marked insulin pen devices as per usual clinical practice.

13.3 Insulin pump with pump suspend feature

During day and night automated closed loop glucose control combined with threshold based pump interruption, the next generation Medtronic subcutaneous insulin infusion pump Medtronic 640G (Medtronic Minimed, Northridge, CA, USA) will be used. Threshold-suspend or predictive low glucose suspend feature will be initially set to suspend insulin delivery at sensor glucose values of 3.3 mmol/l or higher, after which the setting could range from 3.3 to 5.0 mmol/l.

To download insulin pump data during closed loop intervention Medtronic CareLink® Therapy Management Software or similar will be used.

For the follow up closed-loop platform (CamAPS FX), the Dana insulin pump (SOOIL) will be used. Glooko/Diasend® software or similar will be used to download insulin pump data at regular intervals.

13.4 Continuous subcutaneous glucose monitor

13.4.1 Blinded Continuous Subcutaneous Glucose Monitor

FreeStyle Libre Pro sensor (Abbott Diabetes Care, Alameda, CA) or similar blinded CGM devices will be used. Insertions will be done by the research team according to manufacturer's instruction. FreeStyle Libre Pro automatically stores up to 14 days' worth of glucose data and requires no calibration.

To download blinded sensor data during run-in, interventional and control periods, Abbott Diabetes Care proprietary software or similar will be used.

Subjects in the control intervention already using a FreeStyle Libre sensor can use their own sensor instead of the study blinded FreeStyle LibrePro sensor.

13.4.2 Real-time Continuous Subcutaneous Glucose Monitor

The next generation Medtronic Enlite 3 family real-time sensor with Enlite serter (Medtronic Minimed, Northridge, CA, USA) will be used in the study. The sensor will be calibrated according to manufacturer's instructions with additional calibration checks in the morning and evening.

To download real-time sensor data during closed loop intervention Medtronic CareLink® Therapy Management Software or similar will be used.

For the follow up closed-loop platform (CamAPS FX), the Dexcom G6 real-time sensor with sensor applicator (Dexcom, Northridge, CA, USA) will be the study CGM. The sensor will be calibrated according to manufacturer's instructions.

13.5 CareLink USB link

The Medtronic CareLink™ USB is indicated for use commercially by patients at home and for clinicians in a medical office setting as a means of facilitating communication between Medtronic diabetes therapy management devices that use Paradigm-compatible RF telemetry and a personal computer that uses data management application software. The CareLink USB device will enable data from study insulin pumps to be uploaded to CareLink Clinical.

13.6 Smartphone

An Android smartphone hosting CamAPS FX App (see 4.6) will be used.

13.7 Bayer CONTOUR™ Next Link blood glucose meter

A Bayer Contour Next Link RF enabled BG Meter (Study Meter) will be provided to study participants for use throughout the trial. During closed loop, the meter measures will be used to calibrate the study pump. The study pumps use the calibration point in the real-time algorithm which calculates the sensor glucose values that are displayed to the subject.

13.8 Computer-based algorithm

The Cambridge closed loop controller has been used safely and effectively in the closed loop studies in both children and adults with T1D **(study REC Ref. 06/Q0108/350, REC Ref. 07/H0306/116, REC Ref. 08/H0304/75, REC Ref. 08/H0308/297, REC Ref. 09/H0306/44, REC Ref. 10/H0304/87, REC Ref. 12/EE/0155, REC Ref. 12/EE/0034, and REC Ref. 12/EE/0424).**

Boughton C, *et al. BMJ Open* 2020; 10:e033500. doi: 10.1136/bmjopen-2019-033500

13.9 Actiwatch

An Actiwatch (Philips Respironics, Bend, Oregon, USA) will be used to measure sleep over 7 day periods at 6, 12, and 24 months during each intervention arm

14 Data analysis

Analyses of study data will be conducted to address the primary and secondary objectives of the trial. All randomised participants will be included in the analysis according to intention to treat principle.

14.1 Primary analysis

The primary analysis will evaluate between group differences in the levels of the mean area under the stimulated C-peptide curve of mixed meal glucose tolerance test conducted 12 months post diagnosis.

14.2 Secondary analysis

14.2.1 Biochemical evaluation

Secondary endpoints include trends over time in mean stimulated C-peptide AUC, fasting C-peptide, HbA1c levels, percentage of patients in each group with HbA1c <7.5% , insulin dose (U/kg), change in BMI standard deviation scores, change in blood pressure, and change in lipid profile. Based on CGM glucose levels during periods of blinded CGM sensors wear, the between group differences of the following parameter will be assessed: AUC less than 3.9mmol/l and 3.5mmol/l, time spent below target glucose (<3.9 mmol/l), time spent above target glucose (>10.0 mmol/l), time spent within the target glucose range (3.0m mol/l – 10.0 mmol/l), average, standard deviation, and the coefficient of variation of glucose levels, the time with glucose levels < 3.5 mmol/l, 3.0 mmol/l and <2.8 mmol/l, the time with glucose levels in the significant hyperglycaemia (glucose levels > 16.7 mmol/l), total, basal and bolus insulin dose. Trends in CGM and insulin data collected within intervention arms will be evaluated on a 3-monthly basis and on a 12 monthly basis during the extension phase. CGM and insulin data collected during intervention arms will also be compared to pre-intervention baseline CGM readings.

14.2.2 Safety evaluation

Safety data including severe hypoglycaemia events and ketone-positive hyperglycaemia will be tabulated for all subjects, including drop-outs and withdrawals, irrespective of whether CGM data are available and irrespective of whether closed loop was operational. For purposes of analysis, a severe hypoglycaemic event will be defined as an event requiring assistance of another person actively to administer carbohydrate, glucagon, or other resuscitative actions. These episodes may be associated with sufficient neuroglycopaenia to induce seizure or coma. If plasma glucose

measurements are not available during such an event, neurological recovery attributable to the restoration of plasma glucose to normal is considered sufficient evidence that the event was induced by a low plasma glucose concentration.

14.2.3 Human factors evaluation

14.2.3.1 Questionnaires

Descriptive tabulations of questionnaires will be carried out, and scores will be calculated using provided scaling and scoring tools as appropriate.

14.2.3.2 Cognitive evaluation

Change in cognitive test performance from baseline to follow-up will be assessed, as a function of group (intervention or control) and C-peptide level.

14.2.3.3 Qualitative interviews

To maximise rigour at least two experienced qualitative researchers will be involved in data analysis. A thematic analysis will be undertaken by these individuals who will independently review data and write separate reports before attending regular meetings to compare their interpretations and reach agreement on recurrent themes and findings. Interviews will be read through repeatedly and cross-compared in order to identify issues and themes which cut across different people's accounts. A key aspect of the analysis will involve exploringthe experiences and views of participants in the closed loop arm of the trial, to better understand the impact of using a closed loop on diabetes self-management practices and quality of life. Participants accounts of using the closed loop will also be compared with individuals experiences of using MDI to manage Type I Diabetes identified from previously published literature. A final coding frame, reflecting the initial research questions and emergent themes, will be developed once all data have been reviewed and consensus reached on key themes and findings. NVivo9, a qualitative software package, will be used to facilitate data coding/retrieval.

14.2.3.4 Quality of sleep assessment using Actiwatch

Sleep will be automatically scored by Actiware software using previously described and validated algorithms. Sleep duration will be calculated as the sum of all epochs scored as sleep during the time in bed. Variability across nights in a participant's sleep duration will be summarised using the coefficient of variation. Sleep data will be averaged across nights in each participant for each study period.

14.2.3.5 Focus groups

Transcripts of focus group discussions will be thematically analysed using QSR NVivo qualitative analysis software.

14.2.4 Health economics assessment

For each simulation, a simulated cohort of 1,000 patients will be run through the model 1,000 times using first-order Monte Carlo simulation. Long-term outcomes will include total direct costs, life expectancy, quality-adjusted life expectancy and time to onset of complications. Future costs will be discounted at a rate of 3.5% per annum and clinical outcomes discounted at a rate of 1.5% per annum in line with NICE guidance on long-term conditions where treatment effects are sustained over a prolonged period of time (61), if still applicable by the time of analysis. The mean values from the simulation (a total of 1,000 mean values, each from a cohort of 1,000 patients run through the model) will then be used to generate scatterplots of incremental costs versus incremental effectiveness (quality-adjusted life years [QALYs]) for closed loop vs. MDI. Data from the scatterplot will then be used to generate a cost-effectiveness acceptability curve.

Sensitivity analysis

In order to explore the robustness of the base-case findings and establish the key drivers of results, a series of one-way simulations will be performed on those parameters.

14.3 Evaluative periods

Where appropriate, secondary sensor based measures will also be calculated for day and night-time periods. The interval from 8.00 to 24:00 defines day-time period, 00:00 to 08:00 am defines the night-time period.

14.4 Interim monitoring and analyses

Interim analyses of the subset of accumulating data particularly SAEs will be performed at regular intervals (at least annually) for review by the DMEC. The DMEC members will comply with DMEC charter. The DMEC will be asked to give advice on whether the accumulated data from the trial, together with results from other relevant trials, justifies continuing recruitment of further participants or further follow-up.

A detailed interim analysis of 12 month data will be performed.

14.5 Statistical methods

The respective values obtained during the 24-month randomised interventions and the extension phase, contrasting the closed loop against usual care will be compared using an ANCOVA model adjusting for gender, presence or absence of DKA, age and baseline log(C-peptide+1). Primary and secondary analyses will also be conducted to adjust for the baseline C-peptide and HbA1c levels, and by age, BMI, z-score, gender and race/ethnicity, as appropriate. A centre-effect will be explored in the analyses by evaluating for interaction between centre and treatment group on outcome. Secondary per protocol analyses will be conducted. Analyses will be carried out at month 24 and month 48 post-diagnosis.

Primary analysis will be a single comparison and no attempt will be formally made to control the overall type I error rate for the secondary outcomes. For non-normally distributed parameters transformation or nonparametric analyses will be used. A 5% significance level will be used to declare statistical significance for the primary comparison.

Severe hypoglycaemic events and ketone-positive hyperglycaemia will be tabulated in each treatment group, which will be compared using repeated measures logistic regression (generalised estimator equation).

14.6 Adherence and retention

Protocol adherence will be assessed in each treatment group. Tabulations of protocol deviations and unscheduled visits will be included in the analysis. A flow chart will also be used to assess visit completion rates post treatment initiation.

14.7 Sample size and power calculations

The primary analysis will compare the difference between groups in the levels of the 2-hour AUC-mean using the ln(mean C-peptide+1). The residual standard deviation of the ln(x+1) transformed C-peptide AUC analysis of covariance is referred to by TrialNet (Lachin 2011) as the root mean squared error (RMSE). The back-transform, exp(y) – 1, of the mean of the transformed values is referred to by TrialNet as the geometric-like mean. In the DirecNet/TrialNet new onset studies (Lachin 2011), the point estimate for RMSE was 0.18 (transformed scale) and was used in the power calculations. As in the TrialNet sample size calculations, a 50% improvement was assumed in the geometric-like mean C-peptide AUC. The sample size depends on the geometric-like mean value in the control group. The original TrialNet sample size calculations assumed a value of 0.37 pmol/ml for the control group based on the lower 90% confidence limit from previous data (Lachin 2011). The present power calculation applied the same value. A 50% increase in the intervention group of the geometric-like mean C-peptide AUC gives 0.37*1.50 = 0.555pmol/ml. After ln (x+1) transformation, the mean values in the control and treatment groups are 0.315 and 0.441 (transformed scale), respectively, the treatment effect is 0.441-0.315=0.126. The treatment effect of 0.126 with a standard deviation of 0.18 requires 44 subjects per group at 90% power for a two sided-test at the 0.05 level. Allowing for 10% loss to follow up means we would need a total of 96 randomised participants (48 per group).

Boughton C, *et al. BMJ Open* 2020; 10:e033500. doi: 10.1136/bmjopen-2019-033500

14.8 Deviations from the statistical plan

Any deviations from the original statistical plan will be recorded and agreed by the Investigators.

15 Case report forms

The Case Report Form (CRF) is the printed, optical, or electronic document designed to record all the protocol required information to be reported to the Chief Investigator for each study participant.

CRFs will be completed in accordance with GCP and ISO 15197;2013 Guidelines. Corrections to the CRF will be performed by striking through the incorrect entry and by writing the correct value next to the data that has been crossed out; each correction will be initialled and explained (if necessary) by the Investigator or the Investigator's authorised staff.

The electronic CRF system provides an edit feature that records the identity of the person making the change and retains a record of the before and after values of the data field(s) in question. In addition, all eCRF changes require electronic review and signoff by the investigator associated with the visit.

If any amendments to the protocol or other study documents are made, CRFs will be reviewed to determine if an amendment to these forms is also necessary.

16 Data handling

Confidentiality of subject data shall be observed at all times during the study. Personal details including hospital and NHS number for each subject taking part in the research study and linking them to a unique identification number will be held locally on a study screening log in the Trial Site File at each of the study sites. These details will not be revealed at any other stage during the study, and all results will remain anonymous. Electronic case report forms (eCRFs) will be used for recording anonymised study data. eCRFs will be completed in accordance with GCP and ISO 15197: 2013 Guidelines. The study identification number will be used on eCRF and on all the blood /serum/urine samples that are collected throughout the study. Names and addresses will not be used. Collected samples will be stored securely and locked away.

Electronic data will be stored on password-protected computers. All paper records will be kept in locked filing cabinets, in a secure office at each of the study sites. Paper records from transcripts

derived from focus group discussions will be stored without any identifiable information in a research office at the Institute of Public Health in Cambridge.

Only members of the research team and collaborating institutions will have password access to the anonymised electronic data. Only members of the research teams will have access to the filing cabinet. All data will be stored in accordance with the Data Protection Act 1998 and will be archived securely according to Sponsor's archiving policy.

Direct access to the source data will be provided for monitoring, audits, REC review and regulatory authority inspections during and after the study. The fully anonymised data may be shared with third parties (EU or non-EU based) for the purposes of advancing management and treatment of diabetes.

Appropriate procedures agreed by the Chief Investigator and Clinical Principal Investigators will be put in place for data review, database cleaning and issuing and resolving data queries.

17 Study management

The study will be undertaken in accordance with Good Clinical Practice (GCP). All staff will receive appropriate Good Clinical Practice Training. An independent Trial Steering Committee (TSC) and Data Monitoring and Ethics Committee (DMEC) will be appointed for the study. Each recruiting site will have a designated PI. Obtaining consent and recruitment to the study, CGM training, insulin pump training and closed loop system training will be undertaken by appropriately trained members of the local teams. A delegation log will be held at each site listing the responsibilities of staff members

17.1 Trial Steering Committee (TSC)

A trial steering committee consisting of an independent chairperson, two other independent experts in the field of Diabetes and Endocrinology, a public and patient involvement (PPI) representative, and the Chief Investigator (observers: Study Coordinators) will meet bi-annually to provide overall supervision of the trial including progress of the trial, adherence to the protocol, patient safety and the consideration of new information of relevance to the research question. Representatives of the Trial Sponsor and the Trial Funder will be invited to TSC meetings.

17.2 Data Monitoring and Ethics Committee (DMEC)

An independent Data Monitoring and Ethics Committee (DMEC) will comprise a chairperson, and two experts. The DMEC will be informed of all serious adverse events and any unanticipated adverse device effects/events that occur during the study. The DMEC will review compiled adverse event data at periodic intervals. The DMEC will report to the Trial Steering Committee any safety concerns and recommendations for suspension or early termination of the investigation.

17.3 Trial Management Group (TMG)

A trial management group (TMG) consisting of the Chief Investigator, Study Coordinators, and Study Data Manager will be responsible for the day to day management of the trial. Operational aspects of the study will be discussed at least bi-monthly via teleconference. The Principal Clinical Investigators may also participate in the meetings of the TMG.

17.4 Study monitoring

The Study Coordinators on behalf of the Sponsor will ensure that the study is conducted in accordance with GCP standards through site monitoring visits. A monitoring plan will be written and agreed prior to randomisation. Monitoring of the Trial sites will be undertaken by the CCTU according to monitoring plan.

18 Responsibilities

18.1 Chief Investigator

The Chief Investigator (CI) is the person with overall responsibility for the research and all UK ethical applications will be submitted by the CI. The CI is accountable for the conduct of the study and will ensure that all study personnel are adequately qualified and informed about the protocol, any amendments to the protocol, the study treatments and procedures and their study related duties. The CI should maintain a list of appropriately qualified persons to whom he/she has delegated specified significant study-related duties.

18.2 Principal Clinical Investigators

The Principal Clinical Investigators at each investigation centre will be responsible for the day-to-day conduct of the clinical aspects of the study (e.g. overseeing recruitment of eligible subjects and that the study is run according to GCP).

18.3 Study Coordinators

The Study Coordinators will provide day-to-day support for the sites and provide training through Principal Investigator meetings, site initiation and routine monitoring visits.

19 Ethics

The study will be conducted in accordance with the Declaration of Helsinki Ethical Principles for Medical Research involving Human Subjects (October 2000).

19.1 Research Ethics Committee and Institutional Review Board

Prior to commencement of the study, the protocol, any amendments, subject information and informed consent and assent forms, any other written information to be provided to the subject, subject recruitment procedures, current investigator CVs, and any other documents as required by the Research Ethics Committee or Institutional Review Board will be submitted. Written approval will be obtained from the REC prior to the commencement of the study. Any additional requirements imposed by the REC or regulatory authority shall be followed.

19.2 Informed consent of study subjects

In obtaining and documenting informed consent, the investigator will comply with the applicable regulatory requirements and will adhere to GCP standards and to the ethical principles that have their origin in the Declaration of Helsinki. Prior to the start of the study, the Investigator will obtain favourable ethical opinion of the written informed consent form, assent form and any other written information to be provided to subjects.

Subjects will be given full verbal and written information regarding the objectives and procedures of the study and the possible risks involved. The study team will avoid any coercion or undue improper inducement of the subject to participate and subjects will be given ample time to consider participation in the study. Subjects will be informed about their right to withdraw from the study at any time.

The subject and/or their legal representative will be informed in a timely manner should any new information become available during the course of the study that may affect their well-being, safety and willingness to participate in the study.

Written consent/assent will be obtained from participants and/or guardians/family members according to REC requirements. The signed informed consent forms will be photocopied, originals filed in the Investigator’s Site File, a copy placed in the patient’s notes and a copy given to the subjects.

For subjects wishing to continue with the extension phase of the study, further written consent/assent will be obtained from participants and/or guardians/family members according to REC requirements. The signed informed consent forms will be photocopied, originals filed in the Investigator's Site File, a copy placed in the patient's notes and a copy given to the subjects.

20 Amendments to the protocol

Any substantial amendments to the protocol and other documents shall be notified to, and approved by, the Research Ethics Committee or Institutional Review Board, and the regulatory authority, prior to implementation as per nationally agreed guidelines.

21 Deviations from the protocol

Deviations from the protocol should not occur without prior approval of the REC or Sponsor except under emergency circumstances, to protect the rights, safety and well-being of subjects. If deviations do occur, they will be documented, stating the reason and the date, the action taken, and the impact for the subject and for the study. The documentation will be kept in the Investigator's Site File. Deviations will be logged electronically and will require chief investigator or local principal investigator acknowledgement and sign-off.

Deviations affecting the subject's rights, safety and well-being or the scientific integrity of the study will be reported to the REC and Sponsor as soon as possible/ in a timely manner, following nationally agreed guidelines.

22 Timetable

Inclusion of the first subject in the study is planned to take place in August 2016, with an enrolment period of up to 2.5 years. The study will start with an internal pilot phase which will be competed in the last half of 2017. The expected completion of the last subject is June 2021 and the planned completion of the Clinical Study Report is December 2021.

The expected completion of the last subject in the extension phase is June 2023.

Boughton C, *et al. BMJ Open* 2020; 10:e033500. doi: 10.1136/bmjopen-2019-033500

23 Reports and publications

Data will be submitted for publication in internationally peer-reviewed scientific journals; members of the investigator group will all be co-authors. The privacy of each subject and confidentiality of their information shall be preserved in reports and publication of data.

24 Retention of study documentation

Subject notes must be kept for the maximum time period as permitted by each individual site. Other source documents and the Investigator's Site File must be retained for at least 15 years, in line with the Data Protection Act 1998. The Principal Investigator will archive the documentation pertaining to the study after completion or discontinuation of the study.

25 Indemnity statements

Cambridge University Hospitals NHS Foundation Trust, as a member of the NHS Clinical Negligence Scheme for Trusts, will accept full financial liability for harm caused to participants in the clinical trial caused through the negligence of its employees and honorary contract holders. There are no specific arrangements for compensation should a participant be harmed through participation in the trial, but no-one has acted negligently.

The University of Cambridge will arrange insurance for negligent harm caused as a result of protocol design and for non-negligent harm arising through participation in the clinical trial.

References

1. Tomlinson DR, Gardiner NJ: Glucose neurotoxicity. Nat Rev Neurosci 2008;9:36-45

2. Arbelaez AM, Semenkovich K, Hershey T: Glycemic extremes in youth with T1DM: the structural and functional integrity of the developing brain. Pediatr Diabetes 2013;14:541-553

3. Davis EA, Keating B, Byrne GC, Russell M, Jones TW: Hypoglycemia: incidence and clinical predictors in a large population-based sample of children and adolescents with IDDM. Diabetes Care 1997;20:22-25

4. Blasetti A, Chiuri RM, Tocco AM, Di Giulio C, Mattei PA, Ballone E, Chiarelli F, Verrotti A: The effect of recurrent severe hypoglycemia on cognitive performance in children with type 1 diabetes: a meta-analysis. J Child Neurol 2011;26:1383-1391

5. Barnard K, Thomas S, Royle P, Noyes K, Waugh N: Fear of hypoglycaemia in parents of young children with type 1 diabetes: a systematic review. BMC Pediatr 2010;10:50

6. Johnson SR, Cooper MN, Davis EA, Jones TW: Hypoglycaemia, fear of hypoglycaemia and quality of life in children with Type 1 diabetes and their parents. Diabet Med 2013;30:1126-1131

7. Group DCaCTR: The effect of intensive treatment of diabetes on the development and progression of long-term complications in insulin-dependent diabetes mellitus. The Diabetes Control and Complications Trial Research Group. N Engl J Med 1993;329:977-986

8. Cryer PE: The barrier of hypoglycemia in diabetes. Diabetes 2008;57:3169-3176

9. Wood JR, Miller KM, Maahs DM, Beck RW, DiMeglio LA, Libman IM, Quinn M, Tamborlane WV, Woerner SE: Most youth with type 1 diabetes in the T1D Exchange Clinic Registry do not meet American Diabetes Association or International Society for Pediatric and Adolescent Diabetes clinical guidelines. Diabetes Care 2013;36:2035-2037

10. Steffes MW, Sibley S, Jackson M, Thomas W: Beta-cell function and the development of diabetes-related complications in the diabetes control and complications trial. Diabetes Care 2003;26:832-836

11. Lachin JM, McGee P, Palmer JP, Group DER: Impact of C-peptide preservation on metabolic and clinical outcomes in the Diabetes Control and Complications Trial. Diabetes 2014;63:739-748

12. Vantyghem MC, Raverdy V, Balavoine AS, Defrance F, Caiazzo R, Arnalsteen L, Gmyr V, Hazzan M, Noël C, Kerr-Conte J, Pattou F: Continuous glucose monitoring after islet transplantation in type 1 diabetes: an excellent graft function (β-score greater than 7) Is required to abrogate hyperglycemia, whereas a minimal function is necessary to suppress severe hypoglycemia (β-score greater than 3). J Clin Endocrinol Metab 2012;97:E2078-2083

13. Shah SC, Malone JI, Simpson NE: A randomized trial of intensive insulin therapy in newly diagnosed insulin-dependent diabetes mellitus. N Engl J Med 1989;320:550-554

14. Stiller CR, Dupré J, Gent M, Jenner MR, Keown PA, Laupacis A, Martell R, Rodger NW, von Graffenried B, Wolfe BM: Effects of cyclosporine immunosuppression in insulin-dependent diabetes mellitus of recent onset. Science 1984;223:1362-1367

15. Buckingham B, Beck RW, Ruedy KJ, Cheng P, Kollman C, Weinzimer SA, DiMeglio LA, Bremer AA, Slover R, Tamborlane WV: Effectiveness of early intensive therapy on beta-cell preservation in type 1 diabetes. Diabetes Care 2013;36:4030-4035

16. Phillip M, Danne T, Shalitin S, Buckingham B, Laffel L, Tamborlane W, Battelino T: Use of continuous glucose monitoring in children and adolescents (*). Pediatr Diabetes 2012;13:215-228

17. Kordonouri O, Hartmann R, Pankowska E, Rami B, Kapellen T, Coutant R, Lange K, Danne T: Sensor augmented pump therapy from onset of type 1 diabetes: late follow-up results of the Pediatric Onset Study. Pediatr Diabetes 2012;13:515-518

18. Bergenstal RM, Klonoff DC, Garg SK, Bode BW, Meredith M, Slover RH, Ahmann AJ, Welsh JB, Lee SW, Kaufman FR, Group AI-HS: Threshold-based insulin-pump interruption for reduction of hypoglycemia. N Engl J Med 2013;369:224-232

19. Ly TT, Nicholas JA, Retterath A, Lim EM, Davis EA, Jones TW: Effect of sensor-augmented insulin pump therapy and automated insulin suspension vs standard insulin pump therapy on hypoglycemia in patients with type 1 diabetes: a randomized clinical trial. JAMA 2013;310:1240-1247

20. Hovorka R: Closed-loop insulin delivery: from bench to clinical practice. Nat Rev Endocrinol 2011;7:385-395

21. Hovorka R, Allen JM, Elleri D, Chassin LJ, Harris J, Xing D, Kollman C, Hovorka T, Larsen AM, Nodale M, De Palma A, Wilinska ME, Acerini CL, Dunger DB: Manual closed-loop insulin delivery in children and adolescents with type 1 diabetes: a phase 2 randomised crossover trial. Lancet 2010;375:743-751

22. Elleri D, Allen JM, Kumareswaran K, Leelarathna L, Nodale M, Caldwell K, Cheng P, Kollman C, Haidar A, Murphy HR, Wilinska ME, Acerini CL, Dunger DB, Hovorka R: Closed-loop basal insulin delivery over 36 hours in adolescents with type 1 diabetes: randomized clinical trial. Diabetes Care 2013;36:838-844

23. Nimri R, Danne T, Kordonouri O, Atlas E, Bratina N, Biester T, Avbelj M, Miller S, Muller I, Phillip M, Battelino T: The "Glucositter" overnight automated closed loop system for type 1 diabetes: a randomized crossover trial. Pediatr Diabetes 2013;14:159-167

24. Hovorka R, Elleri D, Thabit H, Allen JM, Leelarathna L, El-Khairi R, Kumareswaran K, Caldwell K, Calhoun P, Kollman C, Murphy HR, Acerini CL, Wilinska ME, Nodale M, Dunger DB: Overnight Closed Loop Insulin Delivery in Young People with Type 1 Diabetes: A Free-Living Randomised Clinical Trial. Diabetes Care 2014;37:1204-1211

25. Thabit H, Tauschmann M, Allen JM, Leelarathna L, Hartnell S, Wilinska ME, Acerini CL, Dellweg S, Benesch C, Heinemann L, Mader JK, Holzer M, Kojzar H, Exall J, Yong J,

Pichierri J, Barnard KD, Kollman C, Cheng P, Hindmarsh PC, Campbell FM, Arnolds S, Pieber TR, Evans ML, Dunger DB, Hovorka R, Consortium A, Consortium Ah: Home Use of an Artificial Beta Cell in Type 1 Diabetes. N Engl J Med 2015;373:2129-2140

26. Nimri R, Muller I, Atlas E, Miller S, Kordonouri O, Bratina N, Tsioli C, Stefanija MA, Danne T, Battelino T, Phillip M: Night glucose control with MD-Logic artificial pancreas in home setting: a single blind, randomized crossover trial-interim analysis. Pediatr Diabetes 2014;15:91-99

27. Tauschmann M, Allen JM, Wilinska ME, Thabit H, Stewart Z, Cheng P, Kollman C, Acerini CL, Dunger DB, Hovorka R: Day-and-Night Hybrid Closed-Loop Insulin Delivery in Adolescents With Type 1 Diabetes: A Free-Living, Randomized Clinical Trial. Diabetes Care 2016;Jan 6 [Epub ahead of print]

28. Leelarathna L, Dellweg S, Mader JK, Allen JM, Benesch C, Doll W, Ellmerer M, Hartnell S, Heinemann L, Kojzar H, Michalewski L, Nodale M, Thabit H, Wilinska ME, Pieber TR, Arnolds S, Evans ML, Hovorka R, Consortium Ah: Day and night home closed-loop insulin delivery in adults with type 1 diabetes: three-center randomized crossover study. Diabetes Care 2014;37:1931-1937

29. Thabit H, Lubina-Solomon A, Stadler M, Leelarathna L, Walkinshaw E, Pernet A, Allen JM, Iqbal A, Choudhary P, Kumareswaran K, Nodale M, Nisbet C, Wilinska ME, Barnard KD, Dunger DB, Heller SR, Amiel SA, Evans ML, Hovorka R: Home use of closed-loop insulin delivery for overnight glucose control in adults with type 1 diabetes: a 4-week, multicentre, randomised crossover study. Lancet Diabetes Endocrinol 2014;2:701-709

30. Barnard KD, Wysocki T, Allen JM, Elleri D, Thabit H, Leelarathna L, Gulati A, Nodale M, Dunger DB, Tinati T, Hovorka R: Closing the loop overnight at home setting: psychosocial impact for adolescents with type 1 diabetes and their parents. BMJ Open Diabetes Res Care 2014;2:e000025

31. Hovorka R, Canonico V, Chassin LJ, Haueter U, Massi-Benedetti M, Orsini Federici M, Pieber TR, Schaller HC, Schaupp L, Vering T, Wilinska ME: Nonlinear model predictive control of glucose concentration in subjects with type 1 diabetes. Physiol Meas 2004;25:905-920

32. Wilinska ME, Chassin LJ, Acerini CL, Allen JM, Dunger DB, Hovorka R: Simulation environment to evaluate closed-loop insulin delivery systems in type 1 diabetes. J Diabetes Sci Technol 2010;4:132-144

33. Wilinska ME, Budiman ES, Taub MB, Elleri D, Allen JM, Acerini CL, Dunger DB, Hovorka R: Overnight closed-loop insulin delivery with model predictive control: assessment of hypoglycemia and hyperglycemia risk using simulation studies. J Diabetes Sci Technol 2009;3:1109-1120

34. Hovorka R, Kumareswaran K, Harris J, Allen JM, Elleri D, Xing D, Kollman C, Nodale M, Murphy HR, Dunger DB, Amiel SA, Heller SR, Wilinska ME, Evans ML: Overnight closed loop insulin delivery (artificial pancreas) in adults with type 1 diabetes: crossover randomised controlled studies. Bmj 2011;342:d1855

35. Swift PG, Diabetes ISfPaA: ISPAD clinical practice consensus guidelines 2006-2007. Diabetes education. Pediatr Diabetes 2007;8:103-109

36. Clarke W, Jones T, Rewers A, Dunger D, Klingensmith GJ: Assessment and management of hypoglycemia in children and adolescents with diabetes. Pediatr Diabetes 2009;10 Suppl 12:134-145

37. Wolfsdorf J, Craig ME, Daneman D, Dunger D, Edge J, Lee W, Rosenbloom A, Sperling M, Hanas R: Diabetic ketoacidosis in children and adolescents with diabetes. Pediatr Diabetes 2009;10 Suppl 12:118-133

38. Varni JW, Curtis BH, Abetz LN, Lasch KE, Piault EC, Zeytoonjian AA: Content validity of the PedsQL™ 3.2 Diabetes Module in newly diagnosed patients with Type 1 diabetes mellitus ages 8-45. Qual Life Res 2013;22:2169-2181

39. Goodman R: The Strengths and Difficulties Questionnaire: a research note. J Child Psychol Psychiatry 1997;38:581-586

40. Green LB, Wysocki T, Reineck BM: Fear of hypoglycemia in children and adolescents with diabetes. J Pediatr Psychol 1990;15:633-641

41. Cox DJ, Irvine A, Gonder-Frederick L, Nowacek G, Butterfield J: Fear of hypoglycemia: quantification, validation, and utilization. Diabetes Care 1987;10:617-621

42. Gonder-Frederick LA, Schmidt KM, Vajda KA, Greear ML, Singh H, Shepard JA, Cox DJ: Psychometric properties of the hypoglycemia fear survey-ii for adults with type 1 diabetes. Diabetes Care 2011;34:801-806

43. Maruff P, Thomas E, Cysique L, Brew B, Collie A, Snyder P, Pietrzak RH: Validity of the CogState brief battery: relationship to standardized tests and sensitivity to cognitive impairment in mild traumatic brain injury, schizophrenia, and AIDS dementia complex. Arch Clin Neuropsychol 2009;24:165-178

44. Buysse DJ, Reynolds CF, Monk TH, Berman SR, Kupfer DJ: The Pittsburgh Sleep Quality Index: a new instrument for psychiatric practice and research. Psychiatry Res 1989;28:193-213

45. Palmer AJ, Roze S, Valentine WJ, Minshall ME, Foos V, Lurati FM, Lammert M, Spinas GA: The CORE Diabetes Model: Projecting long-term clinical outcomes, costs and cost-effectiveness of interventions in diabetes mellitus (types 1 and 2) to support clinical and reimbursement decision-making. Curr Med Res Opin 2004;20 Suppl 1:S5-26

46. Palmer AJ, Roze S, Valentine WJ, Minshall ME, Foos V, Lurati FM, Lammert M, Spinas GA: Validation of the CORE Diabetes Model against epidemiological and clinical studies. Curr Med Res Opin 2004;20 Suppl 1:S27-40

47. McEwan P, Foos V, Palmer JL, Lamotte M, Lloyd A, Grant D: Validation of the IMS CORE Diabetes Model. Value Health 2014;17:714-724

48. National Institute for Health and Care Excellence. NICE clinical guideline 48. MI - secondary prevention: secondary prevention in primary and secondary care for patients following a myocardial infarction.

49. Dyer MT, Goldsmith KA, Khan SN, Sharples LD, Freeman C, Hardy I, Buxton MJ, Schofield PM: Clinical and cost-effectiveness analysis of an open label, single-centre, randomised trial of spinal cord stimulation (SCS) versus percutaneous myocardial laser

revascularisation (PMR) in patients with refractory angina pectoris: The SPiRiT trial. Trials 2008;9:40

50. Cameron CG, Bennett HA: Cost-effectiveness of insulin analogues for diabetes mellitus. CMAJ 2009;180:400-407

51. NICE technology appraisal guidance 94. Statins for the prevention of cardiovascular events [article online], Available from http://www.nice.org.uk/guidance.

52. Youman P, Wilson K, Harraf F, Kalra L: The economic burden of stroke in the United Kingdom. Pharmacoeconomics 2003;21 Suppl 1:43-50

53. UK National Institute for Health and Care Excellence 2008. NICE clinical guideline 73. Chronic kidney disease: early identification and management of chronic kidney disease in adults in primary and secondary care [article online], Available from http://www.nice.org.uk/guidance.

54. UK National Institute for Health and Care Excellence 2008. NICE clinical guideline 87. Type 2 diabetes. The management of type 2 diabetes [article online], Available from htpps://www.nice.org.uk/guidance.

55. Clarke P, Gray A, Legood R, Briggs A, Holman R: The impact of diabetes-related complications on healthcare costs: results from the United Kingdom Prospective Diabetes Study (UKPDS Study No. 65). Diabet Med 2003;20:442-450

56. Meads C, Hyde C: What is the cost of blindness? Br J Ophthalmol 2003;87:1201-1204

57. Ghatnekar O, Willis M, Persson U: Cost-effectiveness of treating deep diabetic foot ulcers with Promogran in four European countries. J Wound Care 2002;11:70-74

58. UK National Health Service reference costs 2012-2013 [article online], Available from https://www.gov.uk/government/publications/nhs-reference-costs-2012-to-2013.

59. British National Formulary [article online], Available from htpps://www.medicinescomplete.com/about/publications.htm.

60. Beaudet A, Clegg J, Thuresson PO, Lloyd A, McEwan P: Review of utility values for economic modeling in type 2 diabetes. Value Health 2014;17:462-470

61. National Institute for Health and Clinical Excellence. Discounting of health benefits in special circumstances [article online], Available from http://www.nice.org.uk/media/955/4F/Clarification_to_section_5.6_of_the_Guide_to_Methods_of_Technolgy_Appraisal.pdf.

Boughton C, *et al. BMJ Open* 2020; 10:e033500. doi: 10.1136/bmjopen-2019-033500

26 Document Amendment History

Version Number	Date	Amendment
1.1	29 July 2016	1. Paragraph on safeguarding children added to section 11.3.8.<br>2. Typographical errors in the protocol corrected.
2.0	06 March 2017	1. The age range throughout the document has been changed from 10 – 17.9 years to 10 – 16.9 years.<br>2. The Clinical PI in Oxford has been updated.<br>3. The composition of the DMEC has been updated.<br>4. All reference to the hypoglycaemia fear questionnaire at baseline has been removed.
3.0	16 June 2017	1. Addition of Royal Hospital for Sick Children, Edinburgh as a participating site<br>2. Contact details updated for Prof Greene
4.0	23 February 2018	1. Study personnel information updated<br>2. Additional exclusion criteria added<br>3. PIC centres added for Oxford Children's Hospital and Southampton Children's Hospital<br>4. Statistical analysis information updated<br>5. INSPIRE and PAID questionnaires included at 12 month and 24 month visit
4.1	15 March 2018	1. Error in table 3 corrected
5.0	29 November 2018	1. Addition of 2 year optional extension phase<br>2. Study personnel information updated<br>3. Clarification on use of FreeStyle Libre added<br>4. Retention strategies added<br>5. Provision for closed loop users to continue to use study devices during transition to usual care
6.0	22 January 2019	*1.* TSC chair details updated<br>*2.* Typographical errors in the protocol corrected<br>*3.* Extension of recruitment window from within 10 business days to 21 calendar days

		4. Transition of Closed-Loop participants from the current system FlorenceM onto a new Closed-Loop platform (CamAPS FX) any time after the completion of 12 months in the study
7.0	30 April 2019	1. Participants randomised to closed loop arm to use the new closed loop platform (CamAPS FX) after recruitment into the study.<br>2. Qualitative interviews to be conducted only on participants in the closed loop arm.
8.0	10 September 2019	1. Sponsor contact details updated<br>2. Update to a trial participating site name<br>3. Qualitative interviews to be conducted at or after 12 months in the study<br>3. Indemnity statements have been updated

Boughton C, *et al. BMJ Open* 2020; 10:e033500. doi: 10.1136/bmjopen-2019-033500
